# MiRNA Profiles of Extracellular Vesicles Secreted by Mesenchymal Stromal Cells—Can They Predict Potential Off-Target Effects?

**DOI:** 10.3390/biom10091353

**Published:** 2020-09-22

**Authors:** Timo Z. Nazari-Shafti, Sebastian Neuber, Ana G. Duran, Vasileios Exarchos, Christien M. Beez, Heike Meyborg, Katrin Krüger, Petra Wolint, Johanna Buschmann, Roland Böni, Martina Seifert, Volkmar Falk, Maximilian Y. Emmert

**Affiliations:** 1 Department of Cardiothoracic and Vascular Surgery, German Heart Center Berlin, 13353 Berlin, Germany; neuber@dhzb.de (S.N.); ana.garcia-duran@charite.de (A.G.D.); exarchos@dhzb.de (V.E.); hmeyborg@dhzb.de (H.M.); falk@dhzb.de (V.F.); 2 German Centre for Cardiovascular Research, Partner Site Berlin, 13353 Berlin, Germany; 3 Berlin Institute of Health Center for Regenerative Therapies, Charité—Universitätsmedizin Berlin, 13353 Berlin, Germany; christien.beez@charite.de (C.M.B.); martina.seifert@charite.de (M.S.); 4 Berlin-Brandenburg School for Regenerative Therapies, Charité—Universitätsmedizin Berlin, 13353 Berlin, Germany; 5 Department of Health Sciences and Technology, ETH Zurich, 8093 Zurich, Switzerland; 6 Clinic for Cardiovascular Surgery, Charité—Universitätsmedizin Berlin, 13353 Berlin, Germany; k.krueger@charite.de; 7 Department of Plastic Surgery and Hand Surgery, University Hospital Zurich, 8091 Zurich, Switzerland; petra.wolint@usz.ch (P.W.); johanna.buschmann@usz.ch (J.B.); 8 White House Center for Liposuction, 8044 Zurich, Switzerland; info@whitehousecenter.ch; 9 Institute of Medical Immunology, Charité—Universitätsmedizin Berlin, Corporate Member of Freie Universität Berlin, Humboldt-Universität zu Berlin, and Berlin Institute of Health, 13353 Berlin, Germany; 10 Institute for Regenerative Medicine, University of Zurich, 8044 Zurich, Switzerland; 11 Wyss Zurich, University of Zurich and ETH Zurich, 8092 Zurich, Switzerland

**Keywords:** mesenchymal stromal cells, extracellular vesicles, microRNA, oncomiR, tumor suppressor, cardioprotection, adipose tissue, cord blood

## Abstract

The cardioprotective properties of extracellular vesicles (EVs) derived from mesenchymal stromal cells (MSCs) are currently being investigated in preclinical studies. Although microRNAs (miRNAs) encapsulated in EVs have been identified as one component responsible for the cardioprotective effect of MSCs, their potential off-target effects have not been sufficiently characterized. In the present study, we aimed to investigate the miRNA profile of EVs isolated from MSCs that were derived from cord blood (CB) and adipose tissue (AT). The identified miRNAs were then compared to known targets from the literature to discover possible adverse effects prior to clinical use. Our data show that while many cardioprotective miRNAs such as miR-22-3p, miR-26a-5p, miR-29c-3p, and miR-125b-5p were present in CB- and AT-MSC-derived EVs, a large number of known oncogenic and tumor suppressor miRNAs such as miR-16-5p, miR-23a-3p, and miR-191-5p were also detected. These findings highlight the importance of quality assessment for therapeutically applied EV preparations.

## 1. Introduction

Mesenchymal stromal cells (MSCs) have been extensively studied in preclinical and clinical trials over the past few decades for their promising capabilities in regenerative medicine [1]. There is consensus that MSCs cannot regenerate damaged human heart tissue. However, preclinical studies showed that MSCs may provide cardioprotective effects after myocardial damage by modulating the immune response, promoting neoangiogenesis, and reducing fibrosis in the myocardial scar [2]. The therapeutic efficacy of MSCs is mainly attributed to their paracrine secretion of various growth factors, chemokines, cytokines, and extracellular vehicles (EVs) [3]. Studies in rodents and pigs showed a reduction in scar size after a single injection of MSCs after myocardial injury [4,5]. In clinical trials, the results regarding the therapeutic effect of MSCs after single treatments in patients with myocardial infarction are more inconsistent [6]. Potential issues associated with the use of MSCs include:

(i) the difficulty in generating a consistent source of cells with a stable phenotype,
(ii) a significant first-pass effect due to entrapment of large cells in the lung and liver microvasculature, and
(iii) patient-specific comorbidities in autologous applications [7].

In addition, less than 2% of the injected human cells remain at the target site after 60 min [8]. In a porcine model of acute myocardial ischemia, intramyocardial injections resulted in a retention rate of just over 10% after 60 min [9]. Furthermore, the same study showed that less than 1% of the engrafted cells were still present four weeks after transplantation. This, in turn, means that the release time of the cardioprotective MSC secretome at the site of injury is significantly shorter than the overall process of myocardial remodeling, which prompted scientists to further investigate the secretome of MSCs, specifically MSC-derived EVs. In general, EVs are membranous nanoparticles produced by cells that are divided into three categories based on their biosynthesis: apoptotic bodies, microvesicles, and exosomes [10]. All of them are considered intercellular messengers that, when stimulated, can transmit biological signals through the blood and lymphatic system to neighboring cells and distant tissues. Proteins, messengerRNAs (mRNAs), and microRNAs (miRNAs) partially encapsulated and protected by the lipid membrane of EVs act as the biological mediators between cells. In fact, gain-of-function and loss-of-function assays have demonstrated that miRNAs transported by EVs are primarily responsible for the cardioprotective effect of MSCs [11]. MiRNAs are short nucleotide sequences of 18–22 base pairs that can bind to the 3′ untranslated region of their target mRNAs, either to interfere with their transport to the ribosome or to prevent their translation at the ribosomal site [12]. Because of their short length, miRNAs usually target more than one mRNA, making specific target prediction difficult. To date, more than 150 miRNAs have been identified in MSC-derived EVs [13]. Although there are some differences in the miRNA profile depending on the source of MSCs, a number of cardioprotective miRNAs have been identified that are commonly transported by EVs from various MSC tissue origins [14]. MiRNAs encapsulated in EVs have several functions including regulation of cell physiology, proliferation, cell differentiation, and apoptosis. For example, they can regulate the expression of members of the hypoxia-inducible factor family, which are important for the modulation of vascular sprouting in the setting of hypoxia, via the RNA interference pathway [15]. Furthermore, miRNAs can also target mRNAs that regulate fibrosis and fibroblast activation, such as tissue growth factor-beta (TGF-beta) and members of the SMAD family [16].

Since it was shown that EVs isolated from MSCs can recapitulate the cardioprotective effects of their parent cells, it was hypothesized that the use of EVs may offer significant advantages over their cellular counterparts due to a higher safety profile, lower immunogenicity, and the inability to directly induce tumors [17]. However, whereas many preclinical studies use multiple direct myocardial injections to deliver EVs, this strategy may not be optimal for many patients in clinical practice. Direct access to the heart (i.e., intracoronary or intramyocardial) is achieved either through catheter-based techniques or by cardiovascular surgery, and both methods are associated with a risk of complications. In turn, a single intramyocardial injection may not be sufficient to improve tissue remodeling after a myocardial injury due to the short half-life of EVs and patient-associated comorbidities that can reduce the intrinsic wound healing capacity seen in healthy animal subjects. As a result, several groups are currently investigating methods for intravenous application of EVs that would allow for sufficient titers of therapeutic EVs in myocardial tissues [18,19]. Despite their small size, EVs, like other lipid-based nanoparticles, undergo a significant first-pass effect with accumulation in the liver and lung tissue [19]. While several teams are currently working on targeted delivery strategies for EVs, another pharmacological component must also be considered: application of EVs over long periods translates into the systemic application of a considerable amount of miRNAs, despite their short half-life of less than 24 h [20]. In the field of cancer biology, a multitude of studies describe the role of miRNAs in cancer progression, transformation, and metastasis. In this context, miRNAs are divided into three classes:

(i) oncogenic miRNAs,
(ii) tumor suppressor miRNAs, and
(iii) miRNAs with a dual role in cancer progression.

However, to the best of our knowledge, likely due to the limited number of preclinical trials with systemic EV applications, their miRNA cargo was not analyzed in connection with possible off-target effects. In particular, the presence or absence of pro-oncogenic miRNAs in EV preparations has not been conclusively proven. These potential risks need to be assessed for the clinical use of EVs, especially when treating patients with undetected tumors or predispositions to tumor development. The aim of the present study was therefore to characterize the miRNA cargo of EVs isolated from two clinically relevant MSC sources (i.e., cord blood (CB) and adipose tissue (AT)) and then to compare the EV miRNA cargo to well-known miRNAs involved in cancer biology.

## 2. Materials and Methods 

### 2.1. Cell Isolation and Cell Culture

Human AT-derived MSCs were isolated from patients undergoing liposuction, as described previously [21]. Four donors (three female, one male, mean age 41.8 ± 9.3 years) were included in this study. None of the lipoaspirate donors were obese (body mass index was below 25 for all donors) and none of the donors reported any medical conditions at the time of liposuction. CB-derived MSCs were isolated from CB of four healthy newborns (two female, two male) at the Charité University Hospital Berlin, as described elsewhere [22]. Neither mother nor infant suffered from any medical conditions at the time of donation. All procedures were approved by the local medical ethics committees (Charité University Hospital Ethics Committee, registration number EA2/178/13; Cantonal Ethics Committee Zurich, registration number KEK-ZH 2010-0476/0) and written consent was obtained from patients or relatives. All MSCs were cultured in MesenPRO RS medium (Life Technologies, Grand Island, NY, USA, catalog no. 12747-010) containing 10% fetal bovine serum (FBS; Life Technologies, Carlsbad, CA, USA, catalog no. 10270106), 1% penicillin/streptomycin (P/S; Merck Millipore, Burlington, MA, USA, catalog no. A2213), and 2 ng/mL recombinant human fibroblast growth factor-basic (FGF-b; PeproTech, Hamburg, Germany, catalog no. 100-18C) in a humidified atmosphere of 5% carbon dioxide at 37 °C.

### 2.2. EV Isolation

EVs were isolated from MSC-conditioned medium using (i) sequential ultracentrifugation (UC) or (ii) the exoEasy Maxi Kit (Qiagen, Hilden, Germany, catalog no. 76064) according to the manufacturer’s instructions. Briefly, MSCs were expanded to a confluence of about 80% and washed once with Dulbecco’s phosphate-buffered saline (DPBS, Dulbecco’s phosphate-buffered saline; Life Technologies, Bleiswijk, The Netherlands, catalog no. 14190-144). The cells were switched to Dulbecco’s modified eagle medium (DMEM 1X)-GlutaMAX (Life Technologies, Paisley, United Kingdom, catalog no. 21885-025) containing 10% exosome-depleted FBS (Life Technologies, Bleiswijk, The Netherlands, catalog no. A2720803), 1% P/S, and 2 ng/mL FGF-b for 48 h, followed by a transfer to starvation medium (DMEM 1X-GlutaMAX supplemented with 1% P/S and 2 ng/mL FGF-b) for 24 h. For the isolation of EVs using sequential UC, the supernatant of approximately 3 × 10^7^ cells at early passages (passages 5–7) was processed according to the protocol of Beez et al. [23]. For the isolation of EVs using the Qiagen kit, an MSC-conditioned medium of approximately 3 × 10^6^ cells at early passages was collected and centrifuged at 2000× *g* for 15 min at 4 °C (Allegra X-15R Centrifuge, Beckman Coulter, Indianapolis, IN, USA). The supernatant was decanted and filtered using a 0.2 μm syringe filter (Sartorius, Hanover, Germany, catalog no. 16534) to remove any remaining cell debris and large aggregates. Thereafter, 8 mL of the filtered solution were mixed with 8 mL XBP buffer by gently inverting the tube. The mixture was transferred to the exoEasy spin column, centrifuged at 500× *g* for 1 min at room temperature (R.T) and the flow-through was discarded. Then, the bound EVs were washed with 10 mL XWP buffer and centrifuged at 5000× *g* for 5 min to remove residual buffer from the column. To elute EVs, 0.5 mL XE buffer was added and the column was centrifuged at 500× *g* for 5 min to collect the eluate, which was re-applied to the same column and centrifuged at 5000× *g* for 5 min. Final EV preparations were transferred to low-binding tubes (Sarstedt, Numbrecht, Germany, catalog no. 72.706.600) and stored at −80 °C until further use.

### 2.3. Nanoparticle Tracking Analysis (NTA) and Total Protein Analysis

Particle concentration and size distribution of EV preparations were examined using the ZetaView instrument (Particle Metrix, Inning, Germany). Particles were automatically tracked and sized based on Brownian motion and the diffusion coefficient. The NTA measurement conditions were as follows: temperature = 26.6 ± 2.2 °C, viscosity = 0.87  ± 0.04 cP, frames per second = 30, and measurement time = 75 s. Sample videos were analyzed using NTA software (ZetaView, Particle Metrix, Inning, Germany, version 8.04.02). 

Total protein content of EV preparations was determined using the commercially available Bicinchoninic Acid (BCA) Protein Assay Kit with bovine serum albumin as a standard (Thermo Scientific, catalog no. 23227). Briefly, 20 µL of samples or standards were mixed with 200 µL of freshly made BCA working reagent and incubated for 30 min at 50 °C. Absorbance was measured at 560 nm with a Mithras LB940 plate reader (Berthold Technologies, Pforzheim, Germany) and analyzed with MikroWin 2000 software (Mikrotek Laborsysteme, Overath, Germany, version 4.41).

### 2.4. Transmission Electron Microscopy (TEM)

Isolated EV preparations were stained according to the protocol of Théry et al. [24] and morphologically evaluated at the electron microscopy (EM,) facility of the Charité—Universitätsmedizin Berlin. Briefly, 20 µL of MSC-derived EVs were first placed on formvar carbon-coated copper EM grids (Plano, Wetzlar, Germany, catalog no. G2430N) for 20 min. Then, the samples were incubated for 20 min in 4% paraformaldehyde (Electron Microscopy Sciences, Hatfield, PA, USA, catalog no. 15714), followed by 5 min in 1% glutaraldehyde (Serva, Heidelberg, Germany, catalog no. 23114). After several washing steps with water, the samples were stained for 10 min in a freshly prepared solution of 4% uranyl acetate (Serva, Heidelberg, Germany, catalog no. 77870) and 2% methylcellulose (Sigma-Aldrich, St. Louis, MO, USA, catalog no. M-6385). Imaging was performed using the Leo 906 microscope (Carl Zeiss, Oberkochen, Germany), equipped with ImageSP Viewer software (SYS-PROG, Minsk, Belarus, version 1.2.7.11).

### 2.5. Immunofluorescence Staining and Flow Cytometry

Expression of surface molecules was measured as described before [23]. Briefly, 2 µg of MSC-derived EV protein were incubated with 15 µL of 4 μm aldehyde/sulfate latex beads (Thermo Fisher, catalog no. A37304) for 15 min at R.T. The sample volume was filled up to 1 mL with DPBS and incubated for 1 h at R.T with gentle shaking. Thereafter, samples were centrifuged for 10 min at 300× *g*, and after discarding the supernatant, samples were washed once with 1% fetal calf serum in DPBS (flow cytometry buffer). Next, the beads loaded with EVs were incubated with the following fluorescence-conjugated antibodies: anti-CD9/FITC (BioLegend, San Diego, CA, USA, catalog no. 312104), anti-CD63/PE (BioLegend, San Diego, CA, USA, catalog no. 353004), anti-CD73/APC (BioLegend, San Diego, CA, USA, catalog no. 344006), anti-CD81/FITC (BioLegend, San Diego, CA, USA, catalog no. 349504), anti-HLA-ABC/PE (BioLegend, catalog no. 311405), or anti-HLA-DR/APC (BioLegend, San Diego, CA, USA, catalog no. 307610), each at a dilution of 1:25 in flow cytometry buffer. After 30 min at 4 °C, the beads were washed twice with flow cytometry buffer, fixed with flow cytometry buffer supplemented with 0.5% PFA, and stored at 4 °C until measurement using a MACSQuant VYB flow cytometer (Miltenyi Biotec, Bergisch Gladbach, Germany). Beads incubated with antibodies but no EVs served as negative controls, respectively. Analysis was performed using FlowJo software (Tree Star, Ashland, OR, USA, version 10.6.1).

### 2.6. MiRNA Analysis

MiRNA was extracted from 200 µL of isolated EVs using the miRNeasy Mini Kit (Qiagen, Hilden, Germany, catalog no. 74104) according to the manufacturer’s instructions. The RNA quantity and purity were assessed with the Agilent 2100 Bioanalyzer system (Agilent Technologies, Waldbroon, Germany). Reverse transcription (RT) was performed using the miRCURY LNA Universal cDNA Synthesis Kit II (Exiqon-Qiagen, Hilden, Germany, catalog no. 203301). RT thermocycling parameters were as follows: 42 °C for 60 min and 95 °C for 5 min. Quantitative polymerase chain reaction (qPCR) was performed using the miRCURY LNA Universal RT microRNA PCR system (Exiqon-Qiagen, catalog no. 339340) with 752 known human miRNAs and 3 interplate calibrators and 1 spike-in miRNA as an internal control. All primer/probe sets for miRNAs were custom designed by the supplier. Three extraction controls and two cDNA synthesis controls were additionally used as indicated by the provider. Two real-time qPCR amplifications were performed for each RT reaction. Reactions were performed according to the manufacturers’ instructions using a LightCycler 480 II system (Roche, Rotkreuz, Switzerland). QPCR thermocycling conditions were as follows: 95 °C for 10 min, followed by 40 cycles at 95 °C for 10 s and 60 °C for 1 min. Melt curve analysis was performed between 60 and 95 °C at a ramp rate of 0.11 °C/s. After interpolation calibration, the examined miRNAs were classified into three categories:

(i) miRNAs with mean corrected CT (CTcorr) values below 30.00 were considered as detected with certainty,
(ii) miRNAs with mean CTcorr values between 30.00 and 32.99 were considered as detected with uncertainty, and
(iii) miRNAs with mean CTcorr values equal or greater than 33.00 were considered as not detected.

All analyzed miRNAs and their expression values are listed in Supplementary Materials Table S1. The obtained CT values of miRNAs were normalized using the geNorm method, which calculates a normalization factor based on multiple reference miRNAs [25]. In brief, the arithmetic mean of the CT values of miRNAs that were stably expressed across all samples, namely hsa-miR-1260a, hsa-miR-125b-5p, hsa-miR-21-5p, hsa-miR-23a-3p, hsa-miR-24-3p, hsa-miR-221-3p, hsa-let-7i-5p, hsa-miR-199a-3p, and hsa-miR-100-5p, were subtracted from CTcorr values to calculate delta CT (dCT) values for every sample. In order to plot miRNA expression on heatmaps, Z-scores were determined from logarithmically transformed dCT values for each miRNA. The Z-scores were calculated as a numerical measurement of the mean value group with z = (x − μ)/σ, where x is the raw score, μ is the population mean, and σ is the population standard deviation. Finally, heatmaps of miRNAs were created with the gplots package of RStudio (version 1.3.959). 

### 2.7. Literature Search for miRNAs

A systematic literature search was conducted for all miRNAs with a low mean CTcorr value (≤29.99) in both CB- and AT-MSC-derived EVs. Pubmed, Medline, and Scopus were used as search engines with the following search terms: “name of miRNA”, “name of miRNA” AND “heart”, “name of miRNA” AND “fibrosis”, “name of miRNA” AND “cancer”, “name of miRNA” AND “fibroblasts”, “name of miRNA” AND “endothelial cells”, “name of miRNA” AND “angiogenesis”, “name of miRNA” AND “immunomodulation”, “name of miRNA” AND “macrophages”, “name of miRNA” AND “t-cells”, and “name of miRNA” AND “immune cells”. For published miRNA targets, only studies were considered that confirmed miRNA targets by luciferase reporter assays or gain- and loss-of-function experiments. The findings are summarized in Appendix A Table A1, Table A2 and Table A3. 

### 2.8. Statistical Analysis

GraphPad Prism (GraphPad Software, San Diego, CA, USA, versions 6.0 and 8.3.0) was used for performing data analysis and generating graphs. The statistical significance of differences in EV particle concentration, total protein amount, and surface marker expression was determined by the Mann–Whitney test; a *p*-value of less than 0.05 was considered significant. All miRNA data are shown as median with interquartile range, if not indicated otherwise. Data were tested with Shapiro–Wilk test for normal distribution. Statistical differences between two groups with only one variable in paired observations were determined either with the Wilcoxon matched-pairs signed rank test for non-parametric samples or with the unpaired t-test for parametric samples. Results were considered significant with * *p* < 0.05, ** *p* < 0.01, and *** *p* < 0.001.

## 3. Results

### 3.1. Characterization of EVs

**Figure 1:**
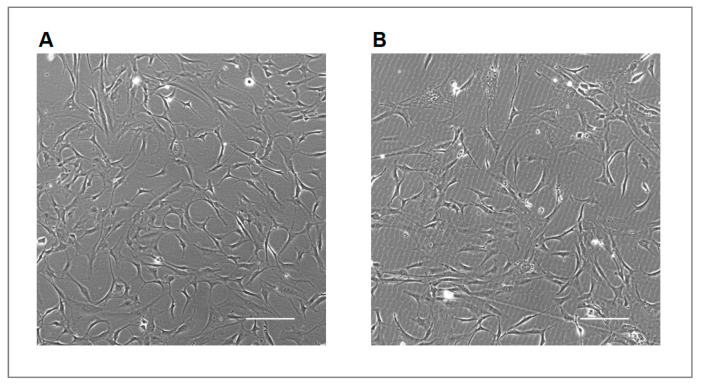
Cord blood (CB)- and adipose tissue mesenchymal stromal cells (AT-MSCs) maintain their spindle-shaped morphology under extracellular vesicles (EV) biogenesis conditions. MSCs were expanded to a confluence of about 80%, washed with Dulbecco’s phosphate-buffered saline and cultivated for 48 h in exosome-depleted medium. Then, the cells were switched to starvation medium for 24 h to derive the conditioned medium for EV isolation. Representative bright-field images of cell morphology of CB-MSCs (**A**) and AT-MSCs (**B**) were taken by phase-contrast microscopy at the time of EV isolation. Bars, 200 µm.

**Figure 2:**
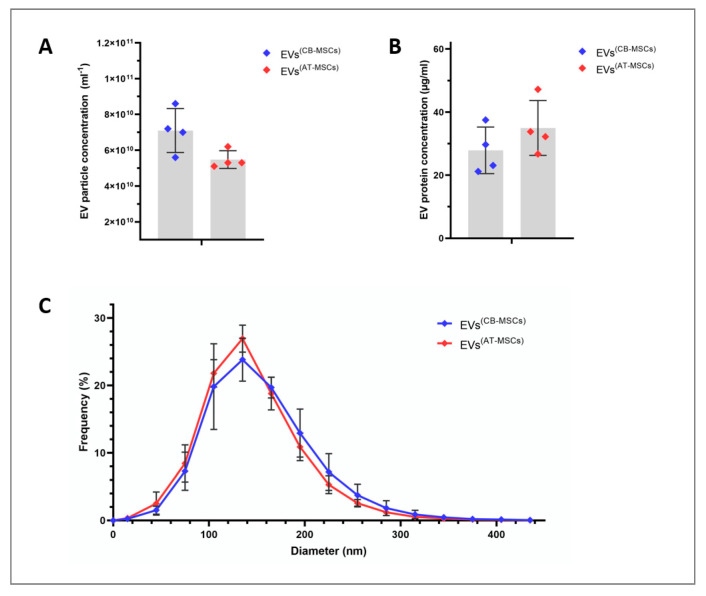
Particle number, protein amount and size distribution of EVs isolated from CB- and AT-MSCs. Particle concentration (**A**) and size distribution (**C**) of EV preparations were measured by nanoparticle tracking analysis. Protein content (**B**) was determined by the bicinchoninic acid assay. In (**A**–**C**), the results are mean values ± standard deviation (SD) obtained from four different donors per cell type.

**Figure 3:**
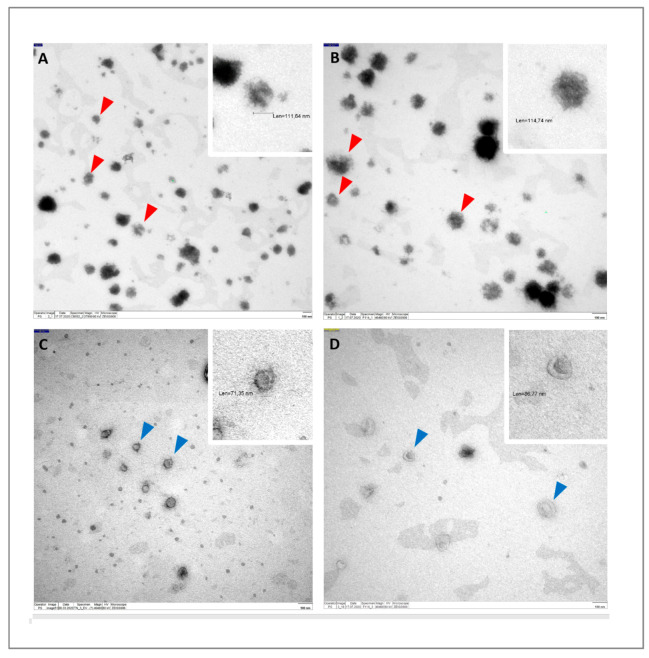
Identification of EV-like structures via transmission electron microscopy. CB- and AT-MSC-derived EVs shown in (**A**,**B**) were isolated using the Qiagen exoEasy Maxi Kit, and CB- and AT-MSC-derived EVs shown in (**C**,**D**) were isolated using sequential ultracentrifugation. All EVs exhibit the expected cup-like shape, an artefact of the fixation method. In (**A**,**B**), EVs are covered with phosphate-rich matter, and red triangles indicate structures in which covered EVs were detected. In (**C**,**D**), exemplary EVs are indicated by blue triangles. In (**A**–**D**), enlarged regions of selected EVs are shown on that top right.

**Figure 4:**
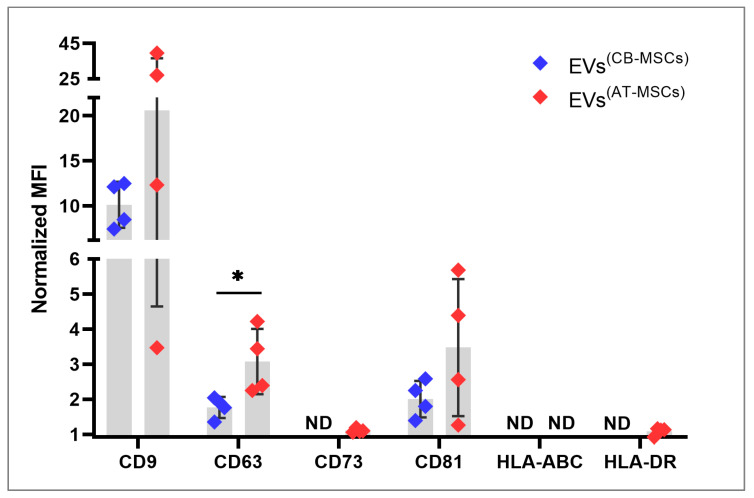
CB- and AT-MSC-derived EVs display a distinct surface marker profile. Detection of the surface marker proteins CD9, CD63, CD73, CD81, HLA-ABC, and HLA-DR using flow cytometry on EV preparations. The data are presented as means ± SD of normalized mean fluorescence intensities (MFIs), which were calculated as the ratio of the geometric MFI of EV samples (beads + EVs + antibodies) to control samples (beads + antibodies). Statistical analysis was performed by the Mann-Whitney test with * *p* < 0.05. ND indicates not detected. EVs from four different donors per cell type were included.

All EVs were harvested from the supernatants of in vitro-cultured CB- and AT-MSCs, which were derived from tissues of four healthy subjects each. Although isolated from different sources, both MSC lines showed a typical spindle-shaped cell morphology under EV biogenesis conditions (Figure 1). The mean number of EV particles obtained was 7.1 ± 1.2 × 10^10^ per mL for CB-MSC-derived EVs and 5.5 ± 0.5 × 10^10^ per mL for AT-MSC-derived EVs (Figure 2A), but this difference was not significant (*p =* 0.057). Similarly, protein concentrations between EVs from CB- and AT-MSCs were not statistically significant (*p =* 0.343), with mean values of 27.9 ± 7.4 and 35.0 ± 8.7 µg/mL protein (Figure 2B). Quantitative analysis of EV diameters demonstrated an asymmetrical distribution, with a mean diameter of 132.7 ± 12.1 nm for EVs from CB-MSCs and a mean diameter of 123.9 ± 6.6 nm for EVs from AT-MSCs (Figure 2C), indicating the presence of exosomes, which are typically 40 to 150 nm in diameter [26]. Furthermore, both EV variants, which were isolated with the Qiagen kit, exhibited typical cup-like shapes as observed by TEM (Figure 3A,B). In comparison, EVs isolated by sequential UC showed a similar shape (Figure 3C,D). However, in contrast to the EVs isolated by UC, the EVs isolated by Qiagen membrane affinity columns were covered by a corona that bound larger amounts of uranyl acetate (Figure 3A,B, red triangles). EVs isolated by sequential UC have not been further examined because this manuscript focuses on EVs isolated by the Qiagen exoEasy Maxi Kit due to its excellent scalability, which is needed for the production of large EV amounts for clinical application. Next, we analyzed the isolated EV preparations for selected membrane proteins that have been associated with EVs in the past. Regardless of the cell source, it was possible to detect on all EV preparations CD9, CD63, and CD81, with CD9 exhibiting the highest normalized mean fluorescence intensities (MFIs) (Figure 4). Interestingly, all of the aforementioned markers tended to have higher values in AT-MSC-derived EVs than in CB-MSC-derived EVs, while only CD63 levels were significantly higher (*p =* 0.029). Figure 4 also shows that CD73 was only detected in EVs from AT-MSCs, but not from CB-MSCs. Since it was hypothesized that MSC-derived EVs do not carry human leukocyte antigens (HLAs) and are therefore less immunogenic [23], we also included HLA-ABC and HLA-DR in the flow cytometry analysis. Our data indicate that EVs from CB-MSCs did not exhibit a signal for HLA-ABC and HLA-DR (Figure 4). For EVs from AT-MSCs, HLA-ABC was also not present, while HLA-DR was detected in small amounts (Figure 4). In sum, these results indicated that the isolated EVs contained exosomes.

### 3.2. MiRNA Profile of CB- and AT-MSC-Derived EVs

**Figure 5:**
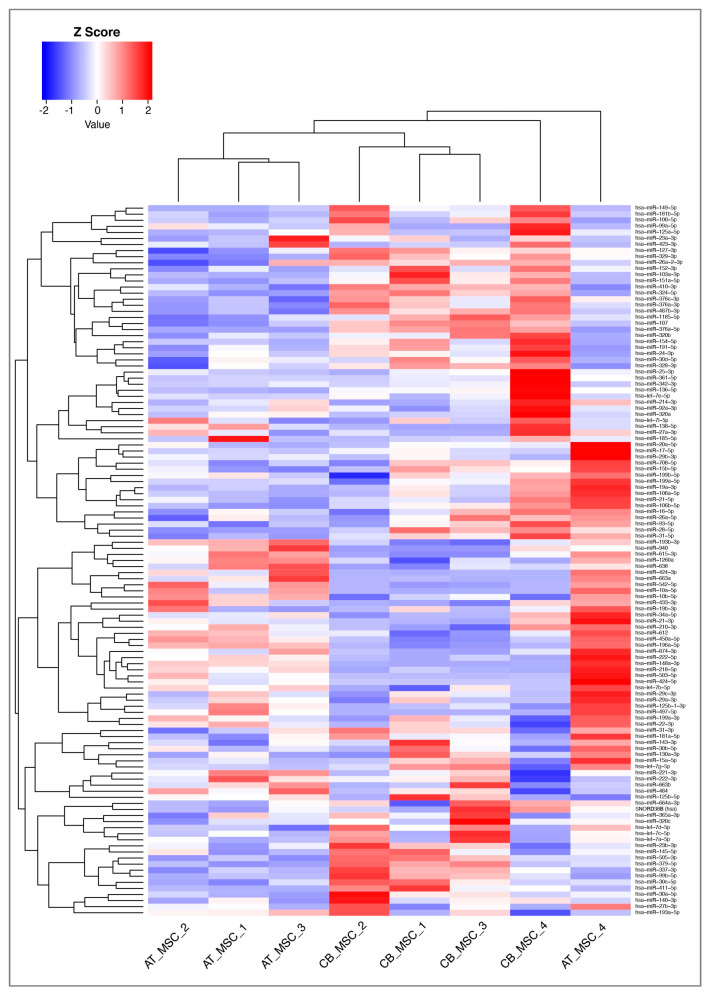
Heatmap and dendrograms of all microRNAs (miRNAs) detected with certainty according to the guidelines of the Qiagen-Exiqon miRCURY LNA Universal RT microRNA PCR system. Sample IDs are shown on the x-axis. Samples with similar miRNA expression are clustered together. The heatmap was generated by RStudio and 2^dCT^ was used for data input. Z-scores of more than zero indicate a higher expression of miRNAs in one sample compared to the others; Z-scores of less than zero indicate the opposite.

**Figure 6:**
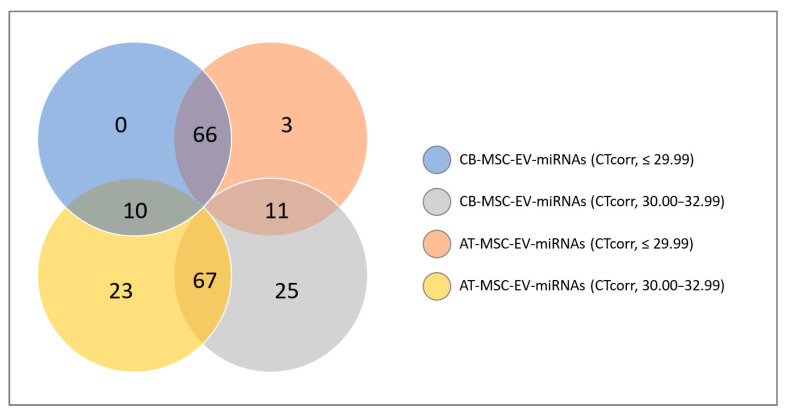
Venn diagram of miRNAs found in CB- and AT-MSC-derived EVs. In total, 752 miRNAs were analyzed and categorized according to mean CTcorr values. High miRNA expression means CTcorr value ≤ 29.99; low miRNA expression means CTcorr value = 30.00–32.99. Five hundred and forty-seven miRNAs were not detected in EVs from CB-MSCs or in EVs from AT-MSCs (mean CTcorr value ≥ 33.00).

**Figure 7:**
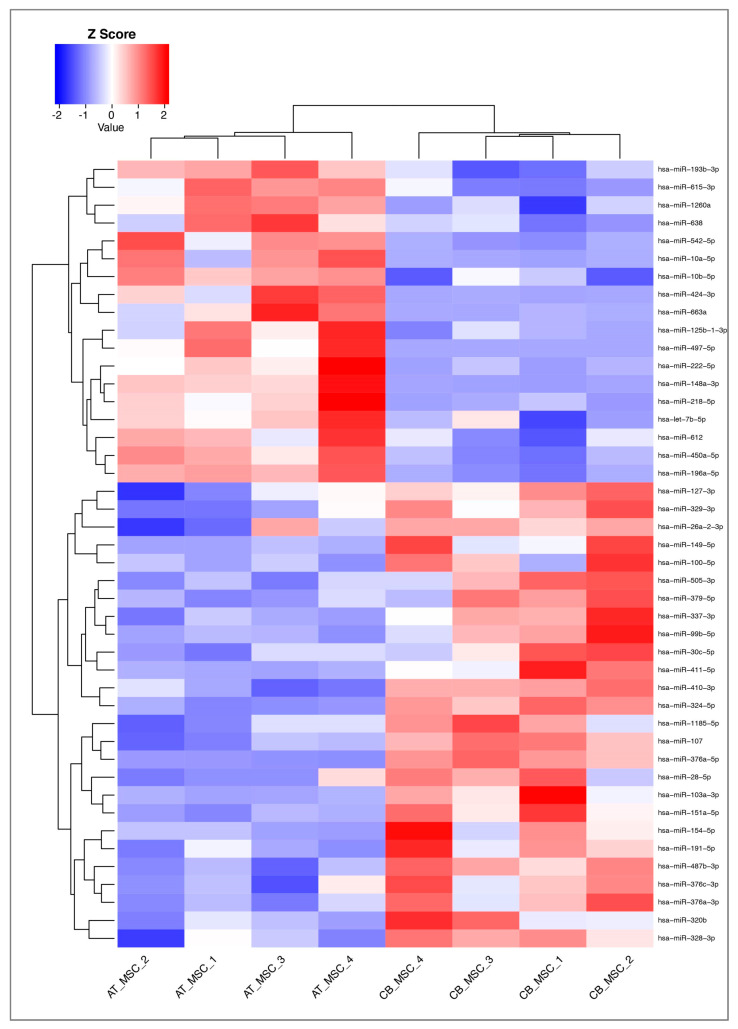
Heatmap and dendrograms of miRNAs that were significantly changed in AT-MSC-derived EVs compared to CB-MSC-derived EVs. Sample IDs are shown on the x-axis. Samples with similar miRNA expression are clustered together. The heatmap was generated by RStudio and 2^dCT^ was used for data input. Z-scores of more than zero indicate a higher expression of miRNAs in one sample compared to the others; Z-scores of less than zero indicate the opposite.

Of the 752 miRNAs examined in this study, 117 were detected with certainty according to the guidelines of the Qiagen-Exiqon miRCURY LNA Universal RT microRNA PCR system. Based on these miRNAs, a heatmap was created (Figure 5). The grouping of donors shows a consistent clustering with only one outlier per group (CB_MSC_4 and AT-MSC_4). Interestingly, the expression profile of EV surface markers for these donors also differed from the other donors in the same group. For further analysis, all miRNAs with mean CTcorr values below 33.00 in at least one group were included. Following this, 205 miRNAs were detected in EV samples, while the majority of miRNAs (547) were not detected (Figure 6). From our analysis, 76 miRNAs were highly expressed in CB-MSC-derived EVs and 80 miRNAs were strongly expressed in AT-MSC-derived EVs with mean CTcorr values of less than 30.00. Intriguingly, among them, 66 miRNAs were found in EVs from both MSC sources. Only 10 were uniquely highly expressed in CB-MSC-derived EVs, namely let-7d-5p, miR-30a-5p, miR-106b-5p, miR-107, miR-136-5p, miR-140-3p, miR-181b-5p, miR-320b, and miR-320c, and miR-342-3p, and 14 were uniquely highly expressed in AT-MSC-derived EVs, namely miR-10b-5p, miR-29b-3p, miR-138-5p, miR-148a-3p, miR-185-5p, miR-210-3p, miR-424-3p, miR-424-5p, miR-433-3p, miR-484, miR-503-5p, miR-663b, miR-874-3p, and miR-940. Furthermore, 100 and 103 miRNAs in CB-MSC-derived EVs and AT-MSC-derived EVs, respectively, which showed mean CTcorr values of 30.00 to 32.99, were considered to be low expressed. To visualize differential miRNA expression profiles, a heatmap of all miRNAs that were significantly different in expression between CB- and AT-MSC-derived EVs was created, showing a clear clustering of CB-MSC-EV-miRNAs and AT-MSC-EV-miRNAs (Figure 7, 44 miRNAs). Overall, the differences in expression after normalization did not exceed a two-fold increase or decrease for almost all miRNAs, except for miR-10b-5p (8.23-fold higher in AT-MSC-derived EVs), miR-103a-3p (3.35-fold higher in CB-MSC-derived EVs), miR-222-5p (8.28-fold higher in AT-MSC-derived EVs), miR-376a-3p (2.45-fold higher in CB-MSC-derived EVs), miR-663a (7.68-fold higher in AT-MSC-derived EVs), and miR-1260a (2.87-fold higher in AT-MSC-derived EVs). Three miRNAs were only found to be highly expressed in AT-MSC-derived EVs, but were absent in CB-MSC-derived EVs, namely miR-148a-3p, miR-424-3p, miR-503-5p. In sum, CB- and AT-MSC-derived EVs are similar in their miRNA composition, with the exception of a small number of miRNAs.

### 3.3. Classification of miRNAs: Tumor Suppressor miRNAs, Oncogenic miRNAs, and Cardioprotective miRNAs

**Figure 8:**
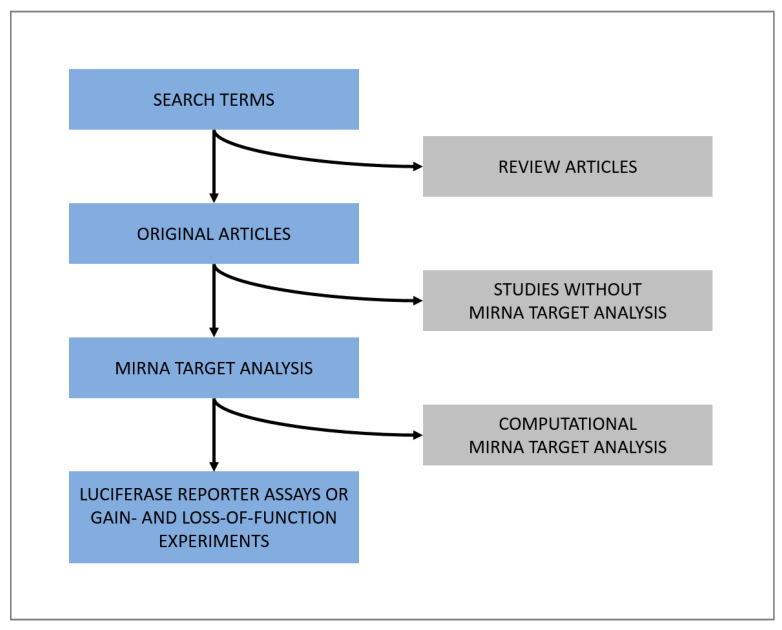
Diagram of literature search rules applied for all miRNAs with a low mean CTcorr value (≤ 29.99) in both CB- and AT-MSC-derived EVs. Search terms were “name of miRNA”, “name of miRNA” AND “heart”, “name of miRNA” AND “cancer”, “name of miRNA” AND “fibrosis”, “name of miRNA” AND “endothelial cells”, “name of miRNA” AND “angiogenesis”, “name of miRNA” AND “immunomodulation”, “name of miRNA” AND “macrophages”, “name of miRNA” AND “t-cells”, and “name of miRNA” AND “immune cells”.

**Figure 9:**
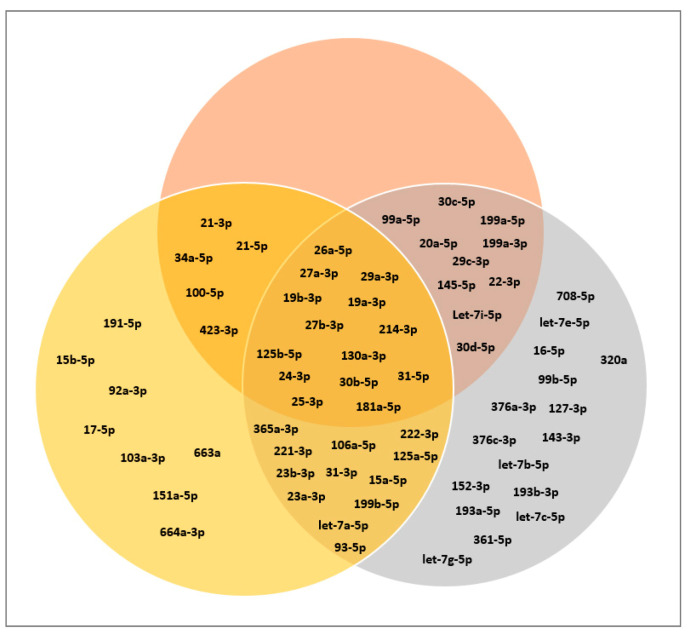
Venn diagram of selected miRNAs based on their function. Gray, tumor suppressor miRNAs; yellow, oncogenic miRNAs; red, cardioprotective miRNAs. With the exception of miR-1260a, all miRNAs with a low mean CTcorr value (≤29.99) in both CB- and AT-MSC-derived EVs were included. MiR-1260a could not be included, as no targets were described in the literature so far. Further details on these miRNAs are given in Table A1, Table A2 and Table A3.

We then conducted a literature research (Figure 8) to group all 66 miRNAs found at high levels in both CB- and AT-MSC-derived EVs based on their function. As indicated in Figure 9, the majority of identified miRNAs have a well-known role as tumor suppressor. We also found many miRNAs, such as miR-103a-3p, miR-151a-5p, and miR-191-5p, which are known oncogenic miRNAs (oncomiRs). Interestingly, we also identified a large number of miRNAs (26) known to act both as oncomiRs and as tumor suppressor. The EV samples examined in this study also showed positive hits for well-known cardioprotective miRNAs, such as miR-21-3p, miR-22-3p, miR-26a-5p, and miR-125b-5p. While having cardioprotective properties, most of them are also associated with oncogenic and tumor suppressor properties. In summary, these data indicate that both CB- and AT-MSC-derived EVs not only transfer a certain set of miRNAs that are involved in one particular mechanism, but rather a multitude of miRNAs that are linked to several biochemical processes, including tumor suppression, tumorigenesis, and cardioprotection.

## 4. Discussion

### 4.1. EV Phenotype

Overall, the EVs analyzed in our study showed the expected proteins to be present in both CB- and AT-MSCs, such as the tetraspanins CD9, CD63, and CD81. The latter was present in significantly lower amounts in EVs from CB-MSCs than in EVs from AT-MSCs, an observation that was not made in other comparative studies before. The phenomenon that EVs from MSCs have only little or no HLAs present on their surface and therefore have a low immunogenicity [23] was confirmed in our study, since HLA-ABC was not found in both CB- and AT-MSC-derived EVs. Furthermore, HLA-DR was not detected in CB-MSC-derived EVs and it was only slightly above the detection level for the flow cytometry assay in AT-MSC-derived EVs. Consequently, the phenotype of the EVs might reflect the low expression of HLA molecules of the parent CB- and AT-MSCs. 

It is known that the isolation method can significantly influence the composition of miRNAs in EV preparations [26,27]. To date, there is a multitude of different EVs isolation protocols available [28], and an ideal isolation method for clinical use remains to be determined. In this study, EVs were isolated using a commercially available EV isolation kit from Qiagen. In contrast to protocols using sequential UC to isolate EVs, this kit is more appropriate for scaling up the production of EVs. Initially, we performed side-to-side comparisons for the isolation of EVs using sequential UC and Qiagen membrane affinity columns. A similar comparison reported by the group of Streanska et al. [29] demonstrated that both methods lead to EVs with encapsulated miRNAs. However, they found differences in EV size and surface protein expression depending on the isolation method. While in their study, they were not able to detect the tetraspanins CD63 and CD81 using the Qiagen kit for EV isolation, we were able to detect tetraspanins such as CD9, CD63 and CD81, considered as typical EV markers. It should be noted, however, that we performed flow cytometry analysis, whereas the others used the Western blot. Furthermore, TEM analysis revealed that EVs isolated by the Qiagen kit were coated with either proteins or nucleic acids. For this experiment, EVs were incubated with uranyl acetate to stain phosphate groups of the lipid membrane. However, the presence of phosphate-rich proteins or nucleic acids in the so-called EV corona can also result in strong staining. We therefore hypothesize that the structures surrounding the EVs are most likely a mixture of proteins and nucleic acids. In line with this, the group of Varga et al. [30] has recently shown that EVs in vivo are also surrounded by a variety of different proteins that are not integrated in their own membrane. Furthermore, Jeppesen et al. [26] were able to separate a protein fraction from a pure vesicle fraction and they demonstrated that different EV isolation methods impact the EV-miRNA composition. Our data suggest that the Qiagen membrane affinity method produces EVs with an intact corona, indicating that miRNAs may also be bound to proteins in the corona. However, it cannot be conclusively determined whether the analyzed miRNAs were encapsulated, bound to co-isolated proteins, or bound within the EV protein corona. Studies that have so far investigated the therapeutic potential of EVs did not purify the EVs in their in vivo models prior to injection. Therefore, regardless of the isolation method, co-purified miRNAs will be injected together with the EV fraction. However, when EVs are used clinically, it is expected that additionally administered miRNAs could also play a role in the cardioprotective mechanism. It is therefore of importance to validate the miRNA profiles for each isolation method before conducting downstream experiments or even clinical studies. A more in depth analysis of the isolated EVs might have answered this question, but would be beyond the scope of this project.

### 4.2. MiRNA Profile

As mentioned above, miRNA analysis of EVs derived from CB- and AT-MSCs showed that a large number of detected miRNAs play an important role in tumor biology. Due to the multiple targets a miRNA can have, it is difficult to predict all possible targets of each miRNA. In this study, we therefore only reviewed targets that were confirmed already by other groups through in vitro assays. Since the PCR array used in our experiment focused on cellular miRNAs, which play a well-known role in cancer biology, it is not surprising that most miRNAs (547 out of 752) were not detected in the EV samples. Our data show a biological variability that is expected from human-derived samples [31]: EV samples derived from both CB- and AT-MSCs contain one outlier in terms of their surface marker configuration and their miRNA profiles (Figure 4 and Figure 7). Due to the small number of donors examined in this study, the effect of donor-specific confounding factors (e.g., gender, age, or race) on the miRNA profiles cannot be determined. Our literature search revealed that most studies focused on the therapeutic aspect of miRNAs of MSC-derived EVs. Only a few studies made the data of their miRNA arrays publicly available [32,33,34]. Additionally, the role of MSC-derived miRNAs in cancer biology has been discussed and investigated by other groups. Even here, however, only few groups made all collected data available for secondary analysis. In the case of AT-MSCs, one group has investigated the role of AT-MSC-derived EVs in the development and treatment of osteoarthritis [32,33]. In both publications, the raw data of the miRNA array were made available by the authors. A side-to-side comparison revealed that 71.0% and 73.3% of the 65 highest expressed miRNAs in both data sets were identical to the miRNAs found in our EV samples. The discrepancy could be explained by the difference in treatment of AT-MSCs at time of isolation and the isolation method itself.

#### 4.2.1. Anti-fibrotic Signaling via Suppression of the TGF-Beta Pathway

MiRNAs were initially examined in the context of cancer biology. Target search was therefore biased and provided a greater number of miRNAs related to cancer than, for instance, to cardioprotection. However, some miRNAs with cardioprotective properties often interfere with proteins that are also regulated in cancer cells. For instance, miRNAs that advantageously modulate fibrosis and activation of fibroblasts usually target either the mRNA of proteins in the TGF-beta/SMAD-axis or promoter and receptor mRNAs that modulate cell cycle activation. Typically, miRNA-mediated suppression of TGF-beta signaling leads to decreased fibrosis in different tissues [35]. Both CB-and AT-MSC-derived EVs contain sets of miRNAs that target TGF-beta receptors directly or downstream signaling proteins such as SMAD proteins. In the context of TGF-beta signaling, SMAD2, 3, and 4 are the downstream promoters that can activate pro-fibrotic gene expression in multiple tissues including the heart [36]. MiR-16-5p (−1.03-fold change, *p =* 0.84), miR-23a-3p (−1.14-fold change, *p =* 0.99), and miR-130a-3p (−1.11-fold change, *p =* 0.75), which showed no difference in relative amounts for the comparison of CB-MSC-derived EVs to AT-MSC-derived EVs, all target the SMAD mRNA directly and exhibit an anti-fibrotic, and in most cancers, a tumor suppressor effect [37,38,39]. At the same time, miR-130a-3p can also act as an oncomiR in esophageal cancer by inhibiting the expression of SMAD4 [40], which incidentally leads to a tumor suppressor effect in hepatoma cells [38]. This dual role of miRNAs in cancer biology is well known and shows the complexity of gene expression regulation via RNA interference [41]. Similarly, while miR-130a-3p suppresses fibrosis in hepatic steatosis by suppressing the TGF-beta receptors 1 and 2 [37], the suppression of TGF-beta receptor 3 by miR-23b-3p and miR-27b-3p in atrial fibroblasts leads to increased fibrosis in the context of atrial fibrillation [42]. This underlines that, similar to the effect of miRNAs in cancer, a dual role of miRNAs and thus potential off-target effects can be hypothesized. It also highlights that adverse effects, such as increased fibrosis, may depend on the presence of miRNA clusters. For the EV samples investigated in the present study, both miR-23b-3p and miR-27b-3p were found with mean CTcorr values of 25.2 ± 1.4 and 25.5 ± 1.1 versus 27.4 ± 0.9 and 27.2 ± 1.1 in CB- and AT-MSC-derived EVs, respectively.

#### 4.2.2. Role of miRNA-Mediated Mammalian Target of Rapamycin (mTOR) Suppression

The miRNA target analysis also revealed that some miRNAs found in CB- and AT-MSC-derived EVs target mTOR or mTOR-associated proteins, including miR-99b/a, miR-100-5p, miR-143-3p, miR-199a-5p/3p, and miR-199b-5p. MTOR is a protein kinase that regulates cell growth, autophagy, and cell survival [43]. Since activation of mTOR plays a crucial role in maintaining growth and inducing metastasis in many cancers, it has been intensively studied as a potential target for cancer therapy [44]. For all of the miRNAs mentioned, overexpression in cancer cell lines led to the induction of apoptosis and autophagy. Interestingly, miR-100-5p can also suppress angiogenesis by preventing cell proliferation in vascular smooth muscle cells, an effect that could counteract a potential cardioprotective effect [45]. Similarly, both miR-143-3p and miR-199a-3p can increase apoptosis during hypoxic or inflammatory injury in kidney and synovial cells, respectively [46,47]. One could therefore postulate that miRNAs that inhibit mTOR signaling are unproblematic in the context of promoting preexisting tumors at the time of EV therapy. However, further studies are needed to elucidate whether MSC-derived EVs suppress mTOR signaling and how this affects the injured heart. There is some evidence that mTOR plays an important role in the activation of cell autophagy in myocardial injuries, which can prevent cell apoptosis and necrosis in the myocardial scar [48]. In the EV samples examined in this work, at least six miRNAs were found that can target mTOR or mTOR signaling related protein mRNAs (Table A1, Table A2 and Table A3). A prolonged exposure to EVs containing these miRNAs may therefore either aggravate myocardial injury by increasing apoptosis in the early stages of myocardial infarction or improve wound healing and remodeling via autophagy.

#### 4.2.3. OncomiRs in MSC-Derived EVs

At least six MSC-EV-miRNAs found in the present study are known oncomiRs, namely miR-24-3p, miR-92a-3p, miR-103a-3p, 151a-5p, miR-191-5p, and miR-423-3p. Remarkably, miR-24-3p and miR-423-3p were also associated with cardioprotective properties. Most of these miRNAs target proteins of the Wnt signaling pathway and/or the phosphatase and tensin homolog deleted from chromosome ten (PTEN) protein (Table A1, Table A2 and Table A3). PTEN is an intracellular membrane-bound phosphatase that hydrolyzes phosphatidylinositol (3,4,5)-trisphosphate to phosphatidylinositol (4,5)-bisphosphate and therefore reduces phosphoinositide-dependent kinase-1- and AKT-mediated activation of cell cycle progression and anti-apoptotic signaling [49]. It is a well-described tumor suppressor and often affected by mutations in various cancers. MiR-103a, for example, targets PTEN in endothelial cells and promotes proliferation and thus angiogenesis [50]. At the same time, miRNA-103a acts as an inhibitor of Wnt signaling in squamous cell carcinoma and promotes cell proliferation [51]. Similarly, the inhibition of Wnt signaling is also promoted by miR-92a and miR-221-3p, which in turn also inhibits PTEN expression in esophageal, gastric, and pancreatic cancer [52,53,54]. While Wnt signaling inhibition and PTEN inhibition are desirable targets for miR-10b-5p, miR-27b-3p, and miR-103a-3p in the context of cardioprotection [50,55,56], this may also promote progression of undetected tumors in recipients of EVs containing miRNAs. 

## 5. Conclusions

The administration of MSC-derived EVs containing miRNAs offers a promising therapeutic approach for cardiovascular disease due to their proposed cardioprotective effects. In the present work, we have isolated EVs from two clinically relevant MSC sources, i.e., CB and AT, using membrane affinity columns and analyzed their miRNA cargo by qRT-PCR. Our data show that EVs from CB- and AT-MSCs are similar in their miRNA composition. Although a large number of miRNAs found in EVs from both MSC sources have been associated with cardioprotective properties, our literature research for known miRNA targets has revealed that they may also play a critical role in the tumor biology of various cancers. Given that EVs and miRNAs have a half-life of less than 24 h, a single administration of EVs may not be sufficient to improve tissue remodeling after a myocardial injury and multiple EV administrations would be required. However, this procedure, in turn, could lead to the accumulation of miRNAs in patients with early-stage cancers that may not have been recognized prior to treatment. Therefore, careful screening of patients for preexisting neoplasms prior to EV administration is important to reduce the risk of potential side effects that could facilitate or even worsen existing tumors. Further reports and functional studies are needed to evaluate both the therapeutic and adverse effects of EVs and their transported miRNAs, depending on the dose and duration of treatment.

## Supplementary Materials

The following are available online at https://www.mdpi.com/2218-273X/10/9/1353/s1, Table S1: Analyzed miRNAs and their expression values.

## Appendix A

**Table A1:** MiRNAs that are known tumor suppressors (TS). Selected miRNAs are also involved in cardioprotection (CP). Targets are given for each miRNA, with no claim to completeness. Pubmed IDs (PMIDs) are given as references when no DOI numbers are available.

MiRNA Function	MiRNA Name	CB-MSC-EV [dCT ± SD]	AT-MSC-EV [dCT ± SD]	Fold Difference	*p*-Value	Confirmed Target GeneGLOBE ID	Cell/Tissue/Cancer Type	MiRNA Cluster	Biological Effect	Reference
TS	miR-127-3p	4.05 ± 0.24	4.98 ± 0.72	–1.9	0.03	BCL6	fibroblasts	—	proliferation inhibition in senescent fibroblasts	doi:10.1371/journal.pone.0080266
						KMT5a	chondrocytes	—	proliferation inhibition in osteoarthritis	doi:10.1016/j.bbrc.2018.06.104
						MMP13	chondrocytes	—	enhances proliferation of chondrocytes in osteoarthritis	doi:10.1111/jcmm.14400
						ITGA6	osteosarcoma	—	tumor suppressor (cell growth, invasion)	doi:10.1002/iub.1710
						KIF3B	squamous cell carcinoma	—	tumor suppressor (cell growth)	doi:10.26355/eurrev_201901_16877
						KIF3B	pancreatic beta cells	—	proliferation inhibition, diabetes	doi:10.18632/aging.101835
TS, CP	miR-30c-5p	4.01 ± 0.5	4.84 ± 0.38	–1.7	0.04	PAI1	breast cancer	—	suppression of vasculogenesis	doi:10.1172/JCI123106
						CTGF	cardiac fibroblasts	miR-133	cardioprotection (anti-fibrotic)	doi:10.1161/CIRCRESAHA.108.182535
						TGFB1, TGFBR2	cardiac fibroblasts	—	suppression of fibrosis	doi:10.1111/jcmm.13548
						CTGF	fibroblasts	—	suppression of cardiac and renal fibrosis	doi:10.1016/j.jdiacomp.2015.12.011
						ADAM19	colorectal carcinoma	—	tumor suppressor (cell proliferation)	doi:10.1371/journal.pone.0120698
						SNAI1	squamous cell carcinoma	—	tumor suppressor (cell proliferation)	doi:10.1016/j.biopha.2017.12.095
						BCL9	prostate cancer	—	tumor suppressor (Wnt signaling suppression, cell proliferation)	doi:10.3892/ol.2016.4161
TS	miR-99b-5p	4.45 ± 0.46	5.45 ± 0.18	–2.01	0.02	mTOR, AKT, IGF1	hepatocytes	—	promotes hepatitis B virus replication	doi:10.1111/cmi.12709
						mTOR, AKT, IGF1	gastric cancer	—	tumor suppressor (cell autophagy)	doi:10.3892/ol.2018.9269
						IGF1	keratinocytes	—	cell proliferation	doi:10.1016/j.biopha.2015.07.013
						PI2K, AKT7, mTOR	cervical cancer	—	tumor suppressor (cell proliferation)	doi:10.1002/jcp.27645
TS	miR-376a-3p	3.76 ± 0.47	5.05 ± 0.51	–2.45	0.01	c-MYC	non-small cell lung carcinoma	—	tumor suppressor (cell proliferation, invasion)	doi:10.1002/cbin.10828
						COA1, PDIA6	giant cell tumor	miR-127-3p	tumor suppressor (cell proliferation, invasion)	doi:10.1016/j.canlet.2017.08.029
						NRP1	breast cancer	—	tumor suppressor (tumor progression)	doi:10.2147/OTT.S173416
						COA1, GLE1, PDIA6	giant cell tumor	miR-127-3p	tumor suppressor (tumor progression)	doi:10.3390/cancers11122019
TS	mir-376c-3p	3.98 ± 0.35	4.85 ± 0.57	–1.81	0.03	HOXB7	squamous cell carcinoma	—	tumor suppressor (cell proliferation)	doi:10.1016/j.biopha.2017.04.050
						BCL2, SYF2	gastric cancer	—	tumor suppressor (cell proliferation)	doi:10.1155/2016/9604257
						CKD1	neuroblastoma cells	—	tumor suppressor (cell proliferation)	doi:10.3892/ol.2018.9431
						HB-EGF	medullary thyroid carcinoma	—	tumor suppressor (cell proliferation)	doi:10.5114/aoms.2019.85244
TS	let-7b-5p	3.03 ± 0.8	1.98 ± 0.36	2.07	0.04	FAS	macrophages	—	inhibits clearance of mycobacterium tuberculosis	doi:10.1093/femsle/fny040
						KIAA1377	squamous cell carcinoma	—	tumor suppressor (cell proliferation, invasion)	doi:10.1002/cbin.11136
						IGF1R	multiple melanoma	—	tumor suppressor (cell proliferation, enhances apoptosis)	doi:10.1093/abbs/gmu089
						CDC25B, CDK1	hepatocellular carcinoma	—	tumor suppressor (cell proliferation, metastasis)	doi:10.1002/jcb.29477
TS	miR-193b-3p	3.16 ± 0.45	2.21 ± 0.16	1.93	0.003	MORC4	breast cancer	—	tumor suppressor (cell proliferation, enhances apoptosis)	doi:10.1002/jcb.27751
						p21-AK2	ovarian carcinoma	—	tumor suppressor (cell autophagy)	doi:10.1016/j.biopha.2017.11.086
						HDAC3	brain	—	suppression of NFkB signaling, reduction of inflammation in brain injury	doi:10.1186/s12974-020-01745-0
						CKD1, AJUBA, HEG1	lung cancer	—	tumor suppressor (cell proliferation, metastasis)	doi:10.1042/BSR20190634
						TGFB1	liver	—	decreases fibrosis	doi:10.1111/jcmm.14210
TS	mir-143-3p	3.28 ± 0.49	3.48 ± 0.51	–1.15	0.6	LIMK1	breast cancer	—	tumor suppressor (tumor progression)	PMID: 28559978
						FOSL2	osteosarcoma	—	tumor suppressor (cell proliferation, invasion, metastasis)	doi:10.1038/s41598-017-18739-3
						BMPR2	bone marrow-derived MSCs	—	promotes cartilage differentiation	doi:10.26355/eurrev_201812_16649
						IGF1R, IGFBP5	synovial cells	—	promotes inflammation and increases apoptosis in RA	doi:10.3892/etm.2018.5907
						BCL2, IGF1R	squamous cell carcinoma	—	tumor suppressor (tumor progression)	doi:10.1016/j.bbrc.2019.08.075
TS, CP	miR-199a-3p	1.92 ± 0.96	1.1 ± 0.45	1.82	0.16	ITGB8	ovarian carcinoma	—	tumor suppressor (chemoresistance)	doi:10.3892/or.2018.6259
						GRP78	non-small cell lung carcinoma	miR-495 (low detection)	tumor suppressor (tumor progression)	doi:10.1016/j.gene.2017.03.032
						DDIT4, ING4	cardiomyocytes	miR-214	cardioprotective (inhibit cardiomyocyte apoptosis during injury)	doi:10.1152/ajpheart.00807.2015
						mTOR	kidney	—	induces injury induced apoptosis	doi:10.1002/jcb.29030
						AXL	osteosarcoma	—	tumor suppressor (tumor progression)	PMID: 25520864
						mTOR	endometrial endometrioid adenocarcinoma	—	tumor suppressor (cell autophagy)	PMID: 31966798
						KL	kidney	—	activation of NFkB signaling in lupus nephritis	doi:10.1016/j.molimm.2018.10.003
						SMAD1	prostate cancer	—	tumor suppressor (cell proliferation, invasion)	doi:10.18632/oncotarget.17191
						mTOR	hepatocellular carcinoma	—	tumor suppressor (chemosensitivity)	doi:10.1186/s13046-019-1512-5
						AGAP2	glioma cells	—	tumor suppressor (tumor progression)	doi:10.18632/aging.102092
						PTGIS	endothelial cells	miR-199a-5p	nitrovasodilatator resistance	doi:10.1161/CIRCULATIONAHA.117.029206
						CD44	hepatocellular carcinoma	—	tumor suppressor (cell proliferation)	doi:10.1016/j.bbrc.2010.10.130
						SOCS7, STAT3	kidney	—	suppress renal fibrosis	doi:10.1038/srep43409
TS, CP	miR-199a-5p	2.63 ± 0.92	2.35 ± 0.65	1.22	0.68831	AA1B	monocytes	—	inhibits differentiation	doi:10.1189/jlb.1A0514-240R
						MAP3K11	non-small cell lung carcinoma	—	tumor suppressor (tumor progression)	doi:10.7150/jca.29426
						SNAI1	papillary thyroid carcinoma	—	tumor suppressor (tumor progression)	doi:10.1016/j.bbrc.2018.02.051
						HIF1A	hemangioma cells	—	tumor suppressor (cell proliferation, autophagy)	doi:10.1177/0394632017749357
						CCR7	bladder cancer	—	tumor suppressor (metastasis)	doi:10.1186/s12894-016-0181-3
						ETS1	breast cancer	—	tumor suppressor (cell invasion)	doi:10.1111/cas.12952
						CLTC	hepatocellular carcinoma	—	tumor suppressor (tumorigenesis)	doi:10.1002/cbf.3252
						PIAS3	cervical cancer	—	tumor suppressor (metastasis, suppresses epithelial–mesenchymal transition)	doi:10.1002/jcb.28631
						ROCK1	colorectal carcinoma	—	tumor suppressor (cell proliferation, metastasis)	doi:10.1177/1533034618775509
						ECE1	spinal cord nerves	—	inhibition of ischemia-reperfusion injury	doi:10.1007/s10571-018-0597-2
						TET2	osteoblasts	—	promote differentiation	doi:10.1016/j.gene.2019.144193
						DDR1	brain	—	protect against ischemia-reperfusion injury	doi:10.1016/j.wneu.2019.07.203
						DRAM1	acute myeloid leukemia	—	tumor suppressor (chemosensitivity)	doi:10.1155/2019/5613417
						CDH1	squamous cell carcinoma	—	tumor suppressor (cell invasion)	doi:10.3892/ol.2016.4602
						MAP4K3	hepatocellular carcinoma	let-7c	tumor suppressor (invasion, metastasis)	doi:10.18632/oncotarget.14623
						ATF6, GRP78	cardiomyocytes	—	downregulation in myocardial hypoxic preconditioning	doi:10.1007/s13105-018-0657-6
						MAP3K11	esophageal cancer	—	tumor suppressor (cell proliferation)	doi:10.18632/oncotarget.6752
						ZEB1	ovarian ectopic endometrial stromal cell	—	inhibition of epithelial–mesenchymal transition	doi:10.1007/s43032-019-00016-5
						HIF1A, OSGIN2	sarcoma	—	tumor suppressor (tumor progression)	doi:10.3892/ol.2016.5320
						PHLPP1	colorectal carcinoma	—	tumor suppressor (chemosensitivity)	doi:10.1517/14728222.2015.1057569
						BIP	kidney	—	protect against ischemia-reperfusion injury	doi:10.1096/fj.201801821R
						WNT2	urothelial cells	—	inhibiting smooth muscle cell proliferation	doi:10.1074/jbc.M114.618694
						MAGT1	gliomal cells	—	tumor suppressor (tumor progression)	doi:10.1002/jcb.28791
						CD44, SIRT1	squamous cell carcinoma	—	tumor suppressor (repress stemness)	doi:10.1080/15384101.2019.1689482
						KL	gastric cancer	—	oncomiR (promotes tumor progression)	doi:10.1186/1471-2407-14-218
						Mrz 08	gliomal cells	—	tumor suppressor (tumor progression)	doi:10.26355/eurrev_201909_18858
						JunB	cardiomyocytes	—	promotes apoptosis in the failing heart)	doi:10.1038/s41598-018-24932-9
						CAV1	lung	—	promotes lung fibrosis	doi:10.1371/journal.pgen.1003291
						NFKB	ovarian carcinoma	—	tumor suppressor (cell proliferation, invasion)	doi:10.3892/ol.2018.9170
						SIRT1, ENOS	endothelial cells	—	promotes migration and tube formation	doi:10.1007/s00705-013-1744-1
						HIF1A	prostate adeno-carcinoma	—	tumor suppressor (tumor progression)	doi:10.18632/oncotarget.18315
TS, CP	miR-99a-5p	4.11 ± 0.87	4.32 ± 0.33	–1.15	0.49	mTOR	urothelial carcinoma	—	tumor suppressor (cell autophagy)	doi:10.2147/OTT.S114276
						HOXA1	smooth muscle cells	—	cardioprotective (inhibits smooth muscle cell proliferation and atherosclerosis)	doi:10.1016/j.lfs.2019.116664
						mTOR	bladder cancer	—	tumor suppressor (cell proliferation)	doi:10.1002/jcb.27318
						NOX4	oral cancer	—	tumor suppressor (cell proliferation, invasion, metastasis)	doi:10.4149/neo_2017_503
						CDC25A	breast cancer	—	tumor suppressor (tumor progression)	doi:10.3390/genes11040369
TS	miR-16-5p	1.76 ± 0.43	1.81 ± 0.33	–1.03	0.84	AKT3	breast cancer	—	tumor suppressor (tumor progression)	doi:10.1042/BSR20191611
						SMAD3	chordoma	—	tumor suppressor (cell proliferation, invasion, metastasis)	doi:10.1038/s41419-018-0738-z
						PIK3R1	fibroblasts	—	inhibits proliferation	doi:10.3390/ijms20051036
						ANXA11	hepatocellular carcinoma	—	tumor suppressor (cell proliferation, metastasis)	doi:10.1186/s13046-019-1188-x
						MYCN	neuroblastoma cells	miR-15a-5p, miR-15b-5p	tumor suppressor (tumor progression)	doi:10.1002/1878-0261.12588
						IGA2	colorectal carcinoma	—	tumor suppressor (cell proliferation, invasion, metastasis)	doi:10.1002/jcp.28747
						SESN1	myoblasts	—	myoblast differentiation and proliferation	doi:10.1038/s41419-018-0403-6
						BACH2	gingival epithelial cells	miR-145-5p	induce apoptosis	PMID: 32509061
						SMAD3	chondrocytes	—	promotes osteoarthritis	doi:10.2174/1381612821666150909094712
						CARM	cervical cancer	—	tumor suppressor (promotes radiosensitivty)	doi:10.1111/pin.12867
						VEGFA	MSCs	—	suppresses osteogenic potential of MSCs	doi:10.18632/aging.103223
						VEGFA	breast cancer	—	tumor suppressor (cell proliferation, invasion, autophagy)	doi:10.18632/oncotarget.20398
						VEGFA	colorectal carcinoma	—	tumor suppressor (cell proliferation, invasion, autophagy)	doi:10.1016/j.omtn.2020.03.006
						EPT1	preadipocytes	—	promotes differentiation	doi:10.1016/j.bbrc.2019.04.179
TS, CP	miR-22-3p	2.52 ± 0.7	1.83 ± 0.31	1.62	0.09	HMGB1	arterial smooth muscle cells	—	inhibits atherosclerosis	doi:10.1159/000480212
						MAPK14	brain	—	prevents Alzheimer’s disease	doi:10.2174/1567202616666191111124516
						WRNIP1	small cell lung cancer	—	tumor suppressor (radiosensitivity)	doi:10.1002/jcb.29032
						AE1	retinoblastoma	—	tumor suppressor (cell proliferation)	doi:10.1016/j.biopha.2018.06.038
						EIF4EBP3	cervical cancer	—	oncomiR (tumuorogenesis	doi:10.7150/ijms.21645
						PTEN	kidney	—	suppresses sepsis-induced kidney injury	doi:10.1042/BSR20200527
						AKT3	Wilm’s tumor	—	tumor suppressor (cell growth)	doi:10.26355/eurrev_202006_21493
						SP1	hepatocellular carcinoma	—	tumor suppressor (cell proliferation, invasion, metastasis)	PMID: 27904693
						SIRT1	peridontal stem cells	—	increases proliferation and differentiation	doi:10.1002/cbin.11271
						PTAFR	cardiac fibroblasts	—	cardioprotective (reduces activation of cardiac fibroblasts)	doi:10.26355/eurrev_202004_20869
						YAP1	non-small cell lung carcinoma	—	tumor suppressor (tumor progression)	doi:10.1111/1759-7714.13280
						DDIT4	glioblastoma	—	tumor suppressor (cell proliferation)	doi:10.1016/j.neulet.2020.134896
						SIRT1	ectopic endometrial cells	—	enhances proliferation and invasion	doi:10.26355/eurrev_202001_20033
						FTO	MSCs	—	promotes osteogenic differentiation	doi:10.1186/s13287-020-01707-6
						NFIB	gastric cancer	—	tumor suppressor (tumor progression)	doi:10.4149/neo_2020_190418N350
TS	miR-152-3p	4.42 ± 0.66	5.16 ± 0.62	–1.9	0.09	SOS1	glioblastoma	—	tumor suppressor (chemosensitivity)	doi:10.2147/OTT.S210732
						p27	chronic myeloid leukemia	—	oncomiR (tumorigenesis)	doi:10.26355/eurrev_201812_16646
						KLF4	prostate cancer	—	tumor suppressor (tumor progression)	doi:10.1002/jcb.28984
						FOXF1	fibroblasts	—	promotes cell proliferation, invasion and extracellular matrix production	doi:10.1016/j.lfs.2019.116779
						CDK8	hepatocellular carcinoma	—	tumor suppressor (cell proliferation)	doi:10.1016/j.prp.2019.03.034
						TMEM97	prostate cancer	—	tumor suppressor (tumor progression)	doi:10.1186/s13148-018-0475-2
						SPIN1	breast cancer	miR-148	tumor suppressor (chemosensitivity)	doi:10.1186/s13046-018-0748-9
						PIK3CA	breast cancer	—	tumor suppressor (tumor progression)	doi:10.3727/096504017x14878536973557
TS, CP	miR-145-5p	1.55 ± 0.65	1.90 ± 0.37	–1.28	0.3	FLT1	trophoblast	—	promote cell proliferation, invasion	doi:10.1016/j.lfs.2019.117008
						KLF4	lung	—	promotes chronic obstructive pulmonary disease	doi:10.1016/j.cbi.2019.01.011
						CD40	cardiomyocytes	—	cardioprotection (in ischemia-reperfusion injury)	doi:10.1007/s11010-017-2982-4
						KLF5	gastric cancer	—	tumor suppressor (tumor progression)	doi:10.1002/jcp.27525
						TAGLN2	bladder cancer	—	tumor suppressor (cell proliferation, invasion, metastasis)	doi:10.3892/ol.2018.9436
						SOX2	breast cancer	—	tumor suppressor (tumor progression)	doi:10.1016/j.jss.2018.11.030
						RHBDD1	colorectal carcinoma	—	tumor suppressor (cell proliferation, invasion, metastasis)	doi:10.1016/j.biocel.2019.105641
						FSCN1	squamous cell carcinoma	—	tumor suppressor (tumor progression)	doi:10.1016/j.ymthe.2018.09.018
						TPT1	prolactinoma	—	tumor suppressor (chemosensitivity)	doi:10.1007/s40618-018-0963-4
						TGFB1	vascular smooth muscle cells	—	inhibits proliferation	doi:10.12659/MSM.910986
						TLR4	melanoma	—	tumor suppressor (cell autophagy)	doi:10.1002/jcb.28388
						SEMA3A	AT-MSCs	—	suppresses osteogenic potential of MSCs	doi:10.1007/s11626-019-00318-7
						AKAP12	prostate cancer	—	tumor suppressor (chemosensitivity)	doi:10.1111/jcmm.13604
						SMAD2/3	hepatocellular carcinoma	—	reduces extracellular matrix production	doi:10.1016/j.bbrc.2019.11.040
						MTDH	squamous cell carcinoma	—	tumor suppressor (tumor progression)	doi:10.1177/1533033819850189
						NRAS	melanoma	—	tumor suppressor (cell proliferation, invasion, metastasis)	doi:10.1002/cam4.1030
						MTDH	non-small cell lung carcinoma	—	tumor suppressor (tumor progression)	doi:10.1096/fj.201701237RR
TS	miR-193a-5p	4.52 ± 0.67	4.35 ± 0.23	1.13	0.82	CDK8	leukemia	—	tumor suppressor (cell proliferation, apoptosis)	doi:10.3892/ijmm.2020.4671
						COL1A1	colorectal carcinoma	—	tumor suppressor (inhibits epithelial–mesenchymal transition)	doi:10.2147/OTT.S255485
						COL1A1	colorectal carcinoma	—	tumor suppressor (inhibits epithelial–mesenchymal transition)	doi:10.3389/fonc.2020.00850
						HOXA1	breast cancer	—	tumor suppressor (tumor progression)	doi:10.18632/aging.103123
						HOXA7	ovarian carcinoma	—	tumor suppressor (cell proliferation, apoptosis)	doi:10.4149/neo_2020_190730N687
						CCNE1	esophageal cancer	—	tumor suppressor (tumor progression)	doi:10.1007/s13402-019-00493-5
						ERBB2	colorectal carcinoma	—	tumor suppressor (tumor progression)	doi:10.2147/CMAR.S234620
						SRSF6	pancreatic cancer	—	oncomiR (metastasis)	PMID: 32064152
						DPEP1	hepatoblastoma	—	tumor suppressor (tumor progression)	doi:10.1038/s41419-019-1943-0
TS, CP	miR-20a-5p	4.83 ± 0.27	4.72 ± 0.93	1.08	0.58	ABCA1	artery smooth muscle cells	—	promotes cell proliferation and migration	doi:10.1002/jbt.22589
						PTEN	endothelial cells	—	pro-angiogenic, inhibits autophagy and apoptosis	doi:10.1038/s41419-020-02745-x
						ERBB2	hepatocellular carcinoma	miR-17-5p	tumor suppressor (metastasis)	doi:10.7150/thno.41365
						TGFBR2	liver	—	anti-fibrotic	doi:10.3389/fonc.2020.00107
						STAT3	endometrial carcinoma	—	tumor suppressor (inhibits epithelial–mesenchymal transition, invasion)	PMID: 31949657
						STAT3	bronchial epithelial cells	—	suppresses apoptosis	doi:10.1016/j.mcp.2019.101499
						SRCIN1	osteoclasts	—	promote proliferation and differentiation	doi:10.1002/cam4.2454
						TGFB1	endothelial cells	—	anti-angiogenic	doi:10.1002/jcp.29111
TS, CP	miR-29c-3p	3.27 ± 0.92	2.48 ± 0.99	1.73	0.28	STAT3	cardiac fibroblasts	—	cardioprotection (inhibits cell proliferation)	doi:10.23736/S0031-0808.20.03975-0
						TNFAIP1	neuroblastoma cells	—	oncomiR (inhibits apoptosis)	doi:10.1007/s11064-020-03096-x
						FOS	lens epithelial cells	—	inhibits epithelial–mesenchymal transition	doi:10.1016/j.biopha.2020.110290
						VEGFA	colorectal carcinoma	—	tumor suppressor (inhibit angiogenesis)	doi:10.1186/s13046-020-01594-y
						TFAP2C	T-cell acute lymphoblastic leukemia	miR-29b-3p	tumor suppressor (cell proliferation)	doi:10.1016/j.bbrc.2020.03.170. Epub 2020
						NFAT	brain	—	inhibit inflammation in Parkinson’s disease	doi:10.1111/gtc.12764
						CCNA2	esophageal cancer	—	tumor suppressor (cell proliferation, migration, and invasion)	doi:10.3389/fbioe.2020.00075
						TRIM31	hepatocellular carcinoma	—	tumor suppressor (tumor progression)	doi:10.3892/or.2020.7469
						FOXP1	ovarian carcinoma	—	tumor suppressor (chemosensitivity)	doi:10.1080/15384101.2019
TS, CP	miR-30d-5p	4.70 ± 0.44	5.43 ± 0.63	1.66	0.09	SIRT1	cardiomyocytes	—	cardioprotection (inhibits hypoxia induced apoptosis)	PMID: 32098921
						SMAD2	ovarian granulosa cells	—	promotes apoptosis	doi:10.3892/etm.2019.8184
						NT5E	prostate cancer	—	tumor suppressor (cell proliferation, migration)	doi:10.1089/cbr.2018.2457
						RUNX2	colon cancer	—	tumor suppressor (tumor progression)	PMID: 29552759
TS	miR-320a	2.51 ± 0.58	2.64 ± 0.20	–1.09	0.60	CXCL9	synovial cells	—	suppress cell proliferation	doi:10.3389/fphys.2020.00441
						SIRT4	ovaries	—	prevent premature ovarian insufficiency	doi:10.1016/j.omtn.2020.05.013
						HIF1A	endometrial carcinoma	—	tumor suppressor (anti-angiogenic)	doi:10.1016/j.yexcr.2020.112113
						SMAD5	bone marrow-derived MSCs	—	promote osteogenic differentiation	doi:10.26355/eurrev_202003_20648
						ANRIL	papillary thyroid carcinoma	—	tumor suppressor (tumorigenesis)	doi:10.1016/j.prp.2020.152856
						LOX1	endothelial cells	—	inhibit apoptosis upon low-density lipoprotein exposure	doi:10.1007/s11010-020-03688-9
						CPEB1	osteosarcoma	—	tumor suppressor (invasion, migration)	doi:10.1002/cam4.2919
						TXNRD1	osteosarcoma	—	tumor suppressor (cell proliferation, migration)	doi:10.1080/15384047.2019.1702405
						FOXM1	hepatocellular carcinoma	—	tumor suppressor (inhibits epithelial–mesenchymal transition, tumor progression)	doi:10.3390/biom10010020
						PBX3	gastric cancer	—	tumor suppressor (tumor progression)	doi:10.4251/wjgo.v11.i10.842
						PKCG	cancer	—	tumor suppressor (cell invasion)	doi:10.1038/s41419-019-1921-6
						MAFB	retina	—	promotes diabetic retinopathy	doi:10.18632/aging.101962
						MAPK	synovial cells	—	promote apoptosis, inhibit proliferation	doi:10.26355/eurrev_201903_17228
						IGFR1	endometrial carcinoma	—	tumor suppressor (tumor progression)	doi:10.3892/ijmm.2019.4051
TS	miR-361-5p	4.82 ± 0.92	5.33 ± 0.31	–1.43	0.34	ITGB1	cervical cancer	—	tumor suppressor (cell proliferation)	doi:10.1007/s43032-019-00008-5
						FOXO1	chondrocytes	—	promotes apoptosis and inhibits cell proliferation	doi:10.1186/s12920-019-0649-6
						SDCBP	gastric cancer	—	tumor suppressor (tumor progression)	doi:10.1097/CAD.0000000000000846
						WT1	hepatocellular carcinoma	—	tumor suppressor (tumorigenesis)	doi:10.26355/eurrev_201910_19277
						ABCA1	vascular smooth muscle cells	—	inhibits proliferation	PMID: 31312370
						CLDN8	retinoblastoma	—	tumor suppressor (cell proliferation, promotes apoptosis)	doi:10.1007/s00381-019-04199-9
						VEGFA	hemangioma cells	—	tumor suppressor (anti-angiogenic)	doi:10.1016/j.bbrc.2019.03.084
						FOXM1	cervical cancer	—	tumor suppressor (tumor progression)	doi:10.1080/21691401.2019.1577883
						FOXM1	osteosarcoma	—	tumor suppressor (tumorigenesis)	doi:10.1002/jcp.28026
						SIRT1	liver	—	promotes hepatosteatosis	doi:10.1016/j.metabol.2018.08.007
						SND1	glioma cells	—	tumor suppressor (invasion, migration)	doi:10.2147/OTT.S171539
						MMP3, MMP9, VEGF	gastric cancer	—	tumor suppressor (inhibits epithelial–mesenchymal transition, tumor progression)	doi:10.1016/j.gene.2018.06.095
						RQCD1	breast cancer	—	tumor suppressor (invasion, migration)	doi:10.17305/bjbms.2018.3399
						ROCK1	papillary thyroid carcinoma	—	tumor suppressor (tumor progression)	doi:10.1016/j.biopha.2018.03.122
						RPL22L1	ovarian carcinoma	—	tumor suppressor (tumorigenesis)	PMID: 31938372
						FOXM1	gastric cancer	—	tumor suppressor (chemoresistance)	doi:10.18632/oncotarget.23513
						FGFR1, MMP1	breast cancer	—	tumor suppressor (cell proliferation, metastasis, metabolism)	doi:10.1186/s13046-017-0630-1
						FOXM1	lung cancer	—	tumor suppressor (tumor progression)	doi:10.4149/neo_2017_406
						TWIST1	glioma cells	—	tumor suppressor (inhibits epithelial–mesenchymal transition)	doi:10.3892/or.2017.5406
TS	miR-708-5p	5.32 ± 0.62	5.41 ± 0.72	–1.06	0.94	PGE2	lung cancer	—	tumor suppressor (tumorigenesis)	doi:10.18632/oncotarget.27614
						CTNNB1	colon cancer	—	tumor suppressor (tumor progression)	doi:10.1016/j.biopha.2020.110292
						TLR4	macrophages	—	immunomodulation of controlling inflammatory factors	doi:10.26355/eurrev_201909_19019
						ZEB1	osteosarcoma	—	tumor suppressor (cell proliferation, invasion)	doi:10.3892/mmr.2019.10013
						URGCP	pancreatic ductal adenocarcinoma	—	tumor suppressor (tumor progression)	doi:10.1016/j.prp.2019.01.026
TS	let-7c-5p	3.67 ± 1.01	3.94 ± 0.38	–1.21	0.43	TGFBR1	kidney	—	chronic kidney disease	doi:10.1155/2020/6960941
						PBX3	squamous cell carcinoma	—	tumor suppressor (tumor progression)	doi:10.1186/s12943-020-01215-4
						CMYC	hepatocellular carcinoma	—	tumor suppressor (cell proliferation)	doi:10.1016/j.bbrc.2019.09.091
						HMGA2	dental pulp stem cells	—	promotes osteogenic differentiation	doi:10.1111/1440-1681.13059
						DMP1MNF	dental pulp stem cells	—	inhibits inflammation	doi:10.12659/MSM.909093
						NAP1L1	hepatocellular carcinoma	—	tumor suppressor (cell proliferation, migration)	doi:10.1016/j.canlet.2018.08.024
TS	let-7e-5p	4.48 ± 1.39	5.18 ± 0.15	–1.63	0.28	CCR7	squamous cell carcinoma	—	tumor suppressor (cell proliferation, metastasis)	doi:10.7150/jca.29536
						FASLG	endothelial progenitors	—	prevents deep vein thrombosis	doi:10.1016/j.thromres.2015.12.020
						RCN1	nasopharyngeal carcinoma	—	tumor suppressor (cell autophagy)	doi:10.1152/ajpcell.00352.2019
TS	let-7g-5p	4.69 ± 1.15	4.60 ± 0.52	1.07	0.84	HMGA2	glioblastoma	—	tumor suppressor (tumor progression)	doi:10.1111/jcmm.14884
						IGF1R	nasopharyngeal carcinoma	—	tumor suppressor (cell migration, invasion)	doi:10.12659/MSM.914555
						PRKCA	mammary cells	—	regulates differentiation	doi:10.1002/jcp.27676
						VSIG4	glioblastoma	—	tumor suppressor (inhibits epithelial–mesenchymal transition)	doi:10.3892/or.2016.5098
TS, CP	let-7i-5p	1.14 ± 0.89	1.39 ± 0.93	–1.19	0.72	GALE	glioblastoma	—	tumor suppressor (cell proliferation, metastasis)	doi:10.2147/CMAR.S221585
						HMGA1	bladder cancer	—	tumor suppressor (cell proliferation, metastasis)	doi:10.1186/s12894-019-0485-1
						CCND2, E2F2	cardiomyocytes	—	cardioprotection (promotes proliferation after injury)	doi:10.1042/CS20181002
						KLK6	colon cancer	—	tumor suppressor (cell proliferation, metastasis)	doi:10.3892/or.2018.6577

**Table A2:** MiRNAs that are known oncomiRs (O). Selected miRNAs are also involved in cardioprotection (CP). Targets are given for each miRNA, with no claim to completeness. Pubmed IDs (PMIDs) are given as references when no doi numbers are available.

MiRNA Function	MiRNA Name	CB-MSC-EV [dCT ± SD]	AT-MSC-EV [dCT ± SD]	Fold Difference	*p*-Value	Confirmed Target GeneGLOBE ID	Cell/Tissue/Cancer Type	MiRNA Cluster	Biological Effect	Reference
O, CP	miR-100-5p	1.11 ± 0.67	2.05 ± 0.28	–1.9	0.05	ANGPT2	hepatocellular carcinoma	—	suppression of angiogenesis	doi:10.1002/path.4804
						p53	pancreatic ductal adenocarcinoma	—	oncomiR (promotes cell growth)	doi:10.1038/s41467-018-03962-x
						mTOR	endometrial carcinoma	miR-199a-3p, miR-199b-5p	tumor suppressor (cell autophagy)	PMID: 31966798
						mTOR	breast cancer	—	tumor suppressor (anti-angiogenic)	doi:10.1007/s13402-017-0335-7
						mTOR	osteosarcoma	—	tumor suppressor (cell autophagy)	doi:10.26355/eurrev_201809_15913
						mTOR	vascular smooth muscle cells	—	suppression of angiogenesis	doi:10.1161/CIRCULATIONAHA.110.000323
O	miR-151a-5p	4.0 ± 0.38	5.09 ± 0.2	–2.14	0.007	CDH1	non-small cell lung carcinoma	—	oncomiR (promotes epithelial–mesenchymal transition, proliferation, invasion)	doi:10.1038/oncsis.2017.66
						p53	nasopharyngeal carcinoma	—	oncomiR (promotes cell proliferation, invasion)	doi:10.1042/BSR20191357
O	miR-103a-3p	2.49 ± 0.64	4.24 ± 0.18	–3.35	0.02	APC/APC2	colorectal carcinoma	miR-1872 (not tested)	oncomiR (activator of Wnt signaling, cell proliferation)	doi:10.1002/jcb.26357
						CDK5	bladder cancer	miR-107	oncomiR (promotes cell proliferation, invasion)	doi:10.1038/emm.2015.39
						CDK6	AT-MSCs	—	inhibit proliferation	doi:10.1038/srep30919
						GPRC5A	prostate cancer	—	oncomiR, tumor suppressor (depending on cancer type)	doi:10.1261/rna.045757.114
						CDH11, NR3C1	squamous cell carcinoma	—	oncomiR (promotes cell proliferation)	doi:10.26355/eurrev_202006_21505
						PTEN	endothelial progenitor cells	—	promotes migration and angiogenesis	doi:10.1016/j.avsg.2019.10.048
						SNRK	glomerular endothelial cells	—	promotes NFkB/p65 activation, renal inflammation and fibrosis	doi:10.1038/s41467-019-11515-z
O	miR-191-5p	3.25 ± 0.42	4.26 ± 0.42	–2.01	0.02	SOX4	breast cancer	—	oncomiR (promotes cell proliferation)	doi:10.1261/rna.060657.117
						EGR1, UBE2D3	hepatocellular carcinoma	—	oncomiR (promotes cell proliferation)	PMID: 31933962
						EGR1	osteosarcoma	—	oncomiR (activates PI3K/AKT pathway, proliferation, invasion)	doi:10.26355/eurrev_201905_17783
						ENOS, MMP1, MMP9	endothelial cells	—	antiangiogenic	doi:10.1096/fj.201601263R
O	miR-92a-3p	3.05 ± 0.75	3.12 ± 0.26	–1.05	0.67	WNT5A	chondrocytes	—	enhance chondrogenesis	doi:10.1186/s13287-018-1004-0
						PTEN	squamous cell carcinoma	—	oncomiR (promotes cell proliferation, metastasis)	doi:10.3892/ijmm.2019.4258
						CDH1	glioma cells	—	oncomiR (promotes tumor progression)	doi:10.3390/ijms17111799
						PTEN	pancreatic cancer	—	oncomiR (promotes cell proliferation, invasion)	doi:10.11817/j.issn.1672-7347.2020.180459
O, CP	miR-423-3p	4.49 ± 0.12	4.47 ± 0.14	1.01	0.83	RAP2C	cardiomyocytes	—	cardioprotection (in ischemic postconditioning secreted by cardiac fibroblast-EVs)	doi:10.1093/cvr/cvy231
						p21CIP1, WAF1	colorectal carcinoma	—	oncomiR (promotes cell growth)	doi:10.1159/000430230
						PANX2	glioma cells	—	oncomiR (promotes tumor progression)	PMID: 29928399
						ADIPOR2	laryngeal cancer	—	oncomiR (promotes tumor progression)	PMID: 25337209
O, CP	miR-21-5p	-0.90 ± 0.52	-0.78 ± 0.82	–1.09	0.98	FASLG	hepatocellular carcinoma	—	oncomiR (chemoresitance)	doi:10.1089/dna.2018.4529
						CCR7	chondrosarcoma	—	tumor suppressor (tumor progression)	doi:10.1080/03008207.2019.1702650
						TIAM1	colon cancer	—	tumor suppressor (cell proliferation, invasion, metastasis)	doi:10.1159/000493457
						PDCD4	breast cancer	—	oncomiR (chemoresitance)	doi:10.4149/neo_2018_181207N930
						BCL2, TLR4	macrophages	—	regulates mycobacterial survival	doi:10.1002/1873-3468.13438
						SET, TAF-IA	lung adenocarcinoma	—	oncomiR (promotes tumor progression)	doi:10.1016/j.lfs.2019.06.014
						RAB11A	neurons	—	neuroprotection during traumatic brain injury	doi:10.12659/MSM.915727
						PTEN, PDCD4	lung	—	anti-apoptotic during ischemia-reperfusion injury	doi:10.1016/j.ejphar.2019.01.022
						SOX7	non-small cell lung carcinoma	—	oncomiR (chemoresitance)	doi:10.2147/OTT.S146423
						TGFB1	non-small cell lung carcinoma	—	oncomiR (promotes cell proliferation)	doi:10.3892/etm.2018.6752
						CHL1	colon adenocarcinoma	—	oncomiR (promotes cell proliferation, invasion)	doi:10.1186/s10020-018-0034-5
						PTEN, PDCD4	lung cancer	—	oncomiR (promotes cell proliferation, metastasis m2 polarization)	doi:10.1186/s13046-019-1027-0
						SMAD7	non-small cell lung carcinoma	—	oncomiR (promotes tumor progression)	doi:10.2147/OTT.S172393
						PI3K	cardiomyocytes	—	cardioprotection (improves contractility)	doi:10.1161/CIRCRESAHA.118.312420
						SPRY1	joints	—	suppresses angiogenesis and matrix degeneration	doi:10.1186/s13075-020-2145-y
						PDCD4	squamous cell carcinoma	—	oncomiR (anti-apoptotic)	doi:10.3892/etm.2019.7970
						MASPIN	endothelial cells	—	suppresses angiogenesis and proliferation	doi:10.1080/09168451.2018.1459179
						CDKN2C	melanoma	—	oncomiR (promotes cell proliferation)	doi:10.1002/2211-5463.12819
						CCL1, TIMP3	neurons	—	inhibits neuropathic pain development	doi:10.1002/jcb.28920
						SMAD7	fibroblasts	—	promote fibrosis in tendon injury	doi:10.1016/j.omtn.2018.11.006
						FASLG	cardiomyocytes	—	cardioprotection (in ischemia-reperfusion injury)	doi:10.1042/BSR20190597
						WWC2	lung adenocarcinoma	—	oncomiR (promotes cell proliferation, metastasis)	doi:10.3233/CBM-201489
						PTEN, mTOR	brain	—	protects against seizure damage	doi:10.1016/j.eplepsyres.2018.05.001
						SMAD7	fibroblasts	—	activation of spinal fibrosis	doi:10.7150/ijbs.24074
						PDCD4	osteosarcoma	—	oncomiR (promotes cell proliferation, metastasis)	doi:10.3892/ijo.2017.4127
						HMSH2	non-small cell lung carcinoma	—	oncomiR (chemoresitance)	doi:10.1159/000481839
						PTEN	smooth muscle cells	—	promotes proliferation and remodeling	doi:10.3390/ijms20040875
						MAPK10	breast cancer	—	oncomiR (promotes tumor progression)	doi:10.1042/BSR20181000
						PTEN	fibroblasts	—	prevents radiation-induced autophagy	doi:10.1038/s41374-019-0323-9
						CADM1	tongue cancer	—	oncomiR (chemoresitance)	doi:10.1007/s00109-016-1417-0
O, CP	miR-34a-5p	3.49 ± 1.45	2.09 ± 0.84	2.65	0.23	NOTCH1	cardiomyocytes	—	cardiotoxic	doi:10.31083/j.rcm.2019.03.545
						BCL2	endothelial cells	—	hypoxia induced autophagy	doi:10.1002/jcb.29207
						ZEB1	cardiomyocytes	—	aggravates hypoxia induced apoptosis	doi:10.1515/hsz-2018-0195
						ACSL1	hepatocytes	—	increases hepatic triglyceride and cholesterol levels	doi:10.3390/ijms20184420
						SIRT1	kidney	—	promotes injury induced fibrosis	doi:10.1038/s41419-018-0527-8
						SIRT1	kidney	—	promotes injury induced fibrosis	doi:10.1016/j.bbrc.2017.12.048
						DLL1	osteosarcoma	—	oncomiR (chemoresitance)	doi:10.1038/srep44218
						AGTR1	osteosarcoma	—	oncomiR (chemoresitance)	doi:10.1186/s12885-016-3002-x
						CD117	osteosarcoma	—	oncomiR (chemoresitance)	doi:10.18632/oncotarget.8546
						PD-L1	ovarian carcinoma	—	oncomiR (chemoresitance)	doi:10.4149/neo_2019_190202N106
						BCL2	ovarian carcinoma	—	oncomiR (promotes cell proliferation)	doi:10.2147/OTT.S142446
O	miR-15b-5p	4.41 ± 0.46	4.88 ± 1.04	–1.38	0.69	AKT3	arteries	—	inhibits ateriogenesis, angiogenesis	doi:10.1161/ATVBAHA.116.308905
						PAQR3	gastric cancer	—	oncomiR (promotes metastasis)	doi:10.3892/or.2017.5673
						AXIN2	hepatocellular carcinoma	—	oncomiR (promotes cell proliferation, invasion)	doi:10.3892/ol.2019.11056
						RECK	prostate cancer	—	oncomiR (tumorigenesis)	doi:10.3892/ol.2019.11056
						SEMA3A	podocytes	—	repressing apoptosis and inflammation in high glucose injury	doi:10.1002/jcp.28691
						PDK4	osteosarcoma	—	oncomiR (promotes cell proliferation)	doi:10.1016/j.bbrc.2018.08.035
						BMPR1A	cardiomyocytes	—	promotes doxorubicin induced injury	doi:10.1007/s12012-018-9495-6
						HPSE2	breast cancer	—	oncomiR (promotes cell proliferation, metastasis)	doi:10.3389/fonc.2020.00108
O	miR-17-5p	5.57 ± 0.15	5.34 ± 0.62	1.10	0.57	BAMBI	nasopharyngeal carcinoma	—	oncomiR (promotes angiogenesis)	doi:10.7150/jca.30757
						ETV1	breast cancer	—	tumor suppressor (cell proliferation)	doi:10.1186/s12885-017-3674-x
						RBL2, E2F4	pancreatic cancer	—	oncomiR (promotes cell proliferation)	doi:10.1016/j.canlet.2017.09.044
						ANKH	fibroblasts	—	increased ostegenesis	doi:10.1016/j.omtn.2019.10.003
						BRCC2	osteosarcoma	—	oncomiR (promotes cell growth)	doi:10.3892/or.2016.4542
						NTN4	breast cancer	—	oncomiR (promotes metastasis, invasion)	PMID: 31933983
						SKSI1	osteosarcoma	—	oncomiR (promotes epithelial–mesenchymal transition)	doi:10.1002/jcb.27832
						SMAD7	fibroblasts	—	promotes liver fibrosis	doi:10.1111/jcmm.14432
						TGFB2	cervical cancer	—	oncomiR (promotes cell proliferation)	doi:10.26355/eurrev_201804_14712
						E2F1	granulosa cells	—	promotes cell proliferation	doi:10.1111/rda.13551
						SMAD5	myoblasts	miR-106b-5p	promotes osteogenic differentiation	doi:10.1016/j.yexcr.2016.07.010
						MFN2	satellite cells	—	modulates mitochondrial function	PMID: 31198013
						P21	nasopharyngeal carcinoma	—	oncomiR (promotes cell proliferation)	doi:10.1002/cam4.863
						HOXB13	prostate cancer	—	oncomiR (promotes tumor progression)	doi:10.1186/s12935-019-0994-8
						P21	astrocytes	—	inhibits apoptosis during hypoxia	doi:10.1186/s12935-019-0994-8
						CMYC	hepatocellular carcinoma	—	tumor suppressor (cell proliferation, invasion, metastasis)	doi:10.1007/s13277-015-4355-5
						VEGFA	endothelial cells	—	mitigates endometriosis	doi:10.1007/s12038-020-00049-y
						TMOD1	gastric cancer	—	oncomiR (tumorigenesis)	doi:10.26355/eurrev_201907_18430
						SMAD7	osteoblasts	—	promotes osteogenic differentiation	doi:10.1038/emm.2014.43
						RUNX3	gastric cancer	—	oncomiR (promotes cell proliferation, metastasis)	doi:10.1016/j.biopha.2020.110246
						SOCS6	gastric cancer	—	oncomiR (promotes cell proliferation)	doi:10.1016/j.febslet.2014.04.036
						PTEN	thyroid cancer	—	oncomiR (promotes cell proliferation)	doi:10.4149/neo_2019_190110N29
						PTEN, GAINT7	hepatocellular carcinoma	—	oncomiR (tumorigenesis)	doi:10.1242/jcs.122895
						PIK3R1	squamous cell carcinoma	—	tumor suppressor (cell autophagy)	doi:10.1007/s10620-012-2400-4
						P21/PTEN	smooth muscle cells	—	promotes hypoxia induced proliferation	doi:10.1186/s12931-018-0902-0
						SMAD7	nasal epithelial cells	—	aggravates inflammatory response	doi:10.1186/s12860-018-0152-5
						TGFB2	gastric cancer	—	oncomiR (promotes cell proliferation)	doi:10.18632/oncotarget.8946
						HBP1	breast cancer	—	oncomiR (promotes metastasis, invasion)	doi:10.1007/s10549-010-0954-4
						SMAD7	hepatic stellate cells	—	activates stellate cells	doi:10.1038/labinvest.2015.58
O, CP	miR-21-3p	5.28 ± 1.27	3.89 ± 1.01	2.26	0.26	SPRY1	fibroblasts	—	promotes wound healing	doi:10.18632/aging.103610
						MAT2B	brain	—	attenuate ischemia-reperfusion injury	doi:10.3325/cmj.2019.60.439
						PTEN	vascular smooth muscle cells	—	promote migration and proliferation (pro-atherogenic)	doi:10.7150/thno.37357
						VEGFA	granulosa cells	—	inhibits autophagy	doi:10.1530/REP-19-0285
						TGS4	retinal pigment epithelial cells	—	modulates apoptosis and inflammation	doi:10.1111/1440-1681.13142
						AKT, CDK2	kidney	—	regulates metabolic alterations in acute kidney injury	doi:10.1155/2019/2821731
						P53	multiple cancers	—	oncomiR (inhibit apoptosis)	doi:10.1016/j.abb.2019.05.026
						PTEN	liver cancer	—	oncomiR (inhibit apoptosis)	doi:10.2147/CMAR.S183328
						HDAC1	epithelium	—	inhibits influenca virus replication	doi:10.3389/fcimb.2018.00175
						SORBS2	cardiomyocytes	—	promoted myocardial dysfunction in sepsis	doi:10.1016/j.yjmcc.2016.03.014
						HDAC1	cardiomyocytes	—	cardioprotection (suppression of myocardial hypertrophy)	doi:10.1093/cvr/cvu254
O	miR-663a	4.43 ± 0.38	1.49 ± 1.06	7.68	0.02	MYL9	osteosarcoma	—	oncomiR (tumorigenesis)	doi:10.1177/0960327120937330
						TGFB1	liver	—	reduces hepatic stellar cell activation	doi:10.1155/2020/3156267
						ZBTB7A	osteosarcoma	—	oncomiR (inhibits apoptosis)	doi:10.1016/j.canlet.2019.01.046
						TGFB1	hepatocellular carcinoma	—	tumor suppressor (cell proliferation, invasion)	doi:10.1186/s12885-018-5016-z
						NFIX	spermatogonial stem cells	—	promote proliferation and inhibit apoptosis	doi:10.1016/j.omtn.2018.05.015
						EMP3	gallbladder cancer	—	oncomiR (tumor progression)	doi:10.1016/j.canlet.2018.05.022
O	miR-664a-3p	4.67 ± 1.55	4.77 ± 0.54	–1.07	0.44	FHL1	lung	—	Progression of chronic obstructive pulmonary disease	doi:10.2147/COPD.S224763
						FOXP3	gastric cancer	—	oncomiR (tumorigenesis)	doi:10.1111/cpr.12567

**Table A3:** MiRNAs that are known for their tumor suppressor and oncogenic potential (TS/O). Selected miRNAs are also involved in cardioprotection (CP). Targets are given for each miRNA, with no claim to completeness. Pubmed IDs (PMIDs) are given as references when no doi numbers are available.

MiRNA Function	MiRNA Name	CB-MSC-EV [dCT ± SD]	AT-MSC-EV [dCT ± SD]	Fold Difference	*p*-Value	Confirmed Targets GeneGLOBE ID	Cell/Tissue/Cancer Type	MiRNA Cluster	Biological Effect	Reference
TS/O	miR-31-3p	4.93 ± 0.56	5.08 ± 0.49	–1.11	0.64	RASA1	colorectal carcinoma	—	oncomiR (promotes cell proliferation, tumor progression)	doi:10.1074/jbc.M112.367763
						SEMA4C	cervical cancer	—	tumor suppressor (chemoresistance)	doi:10.1038/s41598-019-54177-z
						TIAM1	colorectal carcinoma	miR-21	oncomiR (promotes epithelial–mesenchymal transition, invasion)	doi:10.1074/jbc.M110.160069
TS/O	miR-199b-5p	4.39 ± 1.5	3.73 ± 0.43	1.58	0.53	HER2	osteosarcoma	—	oncomiR (promotes tumor progression)	PMID: 30610808
						STON2	papillary thyroid carcinoma	—	tumor suppressor (metastasis, suppresses epithelial–mesenchymal transition)	doi:10.1002/iub.1889
						DYRK1A, NOTCH1, JAG1	—	—	promotes pathological myocardial remodeling	doi:10.1016/j.ncrna.2016.12.002
						KLK10	cervical cancer	—	oncomiR (promotes cell proliferation, metastasis)	doi:10.1016/j.bbrc.2018.05.165
						mTOR	endometrial endometrial adenocarcinoma	miR-100-5p, miR-199a-3p	tumor suppressor (cell autophagy)	PMID: 31966798
						GSK3B	monocytes	—	inhibition of NFkB signaling, anti-inflammatory	doi:10.1007/s10753-018-0799-2
						JAG1	ligamentum flavum cells	—	inhibition of osteogenic differentiation	doi:10.1111/jcmm.13047
						CAV1	non-small cell lung carcinoma	—	oncomiR (promotes cell proliferation)	doi:10.1038/s41419-019-1740-9
						ALK1	breast cancer	—	tumor suppressor (angiogenesis)	doi:10.3389/fgene.2019.01397
						DDR1	breast cancer	—	tumor suppressor (cell proliferation, invasion, metastasis)	doi:10.3892/ol.2018.9255
						BICC1	oral cancer	miR-101-3p (not detected)	tumor suppressor (cell autophagy)	doi:10.1016/j.mcp.2020.101567
						MLK	pancreatic beta cells	—	increases cell proliferation	doi:10.2174/2211536605666160607082214
						JAG1, DDR1	colorectal carcinoma	—	tumor suppressor (cell proliferation, invasion)	doi:10.1002/path.5238
						PODXL, DDR1	acute myeloid leukemia	—	tumor suppressor (cell proliferation)	doi:10.1002/ajh.23129
						HES1	medulloblastoma	—	tumor suppressor (impairs cancer stem cell function)	doi:10.1371/journal.pone.0004998
						ITGA3	squamous cell carcinoma	miR-199a-3p/5p	tumor suppressor (cell proliferation)	doi:10.1111/cas.13298
TS/O	miR-221-3p	1.25 ± 0.67	0.65 ± 0.25	1.51	0.11	AXIN2	-	miR-15b-5p	oncomiR (promotes cell proliferation, invasion)	doi:10.3892/ol.2019.11056
						THBS2	squamous cell carcinoma	—	oncomiR (promotes angiogenesis)	doi:10.1007/s10456-019-09665-1
						SDF1	cartilage	—	prevent cartilage degradation in osteoarthritis	doi:10.1007/s00109-017-1516-6
						VASH1	squamous cell carcinoma	—	oncomiR (promotes metastasis)	doi:10.1038/s41388-018-0511-x
						THBS1	trophoblast	—	promotes invasion and proliferation	doi:10.1016/j.biopha.2018.10.009
						JAK3	macrophages	—	regulates M1 to M2 transition	doi:10.3389/fimmu.2019.03087
						ARF4	epithelial ovarian cancer	—	tumor suppressor (cell proliferation, metastasis)	doi:10.1016/j.bbrc.2017.01.002
						THBS2	squamous cell carcinoma	—	oncomiR (promotes metastasis)	doi:10.1038/s41419-017-0077-5
						MMP22	macrophages	—	prevent low-density lipoprotein-induced oxidative stress	doi:10.1002/jcb.27917
						EIF5A2	medulloblastoma	—	tumor suppressor (cell proliferation, enhances apoptosis)	doi:10.1080/09168451.2018.1553604
						PTEN	gastric cancer	—	oncomiR (promotes tumor progression)	doi:10.3727/096504016 × 14756282819385
						TIMP3	retina	—	promotes microvascular dysfunction	doi:10.1007/s00424-020-02432-y
						PARP1	breast cancer	—	tumor suppressor (tumor progression)	doi:10.18632/oncotarget.21561
						RB1	pancreatic cancer	—	oncomiR (chemoresistance)	doi:10.1007/s13277-016-5445-8
						JNK1, TGFBR1, ETS-1	cardiac fibroblasts	—	cardioprotective (inhibits fibroblast activation)	doi:10.1161/HYPERTENSIONAHA.117.10094
TS/O, CP	miR-25-3p	4.52 ± 0.53	4.8 ± 0.13	–1.21	0.33	BTG2	breast cancer	—	tumor suppressor (cell proliferation)	doi:10.1186/s12943-017-0754-0
						PTEN	retinoblastoma	—	oncomiR (promotes tumor progression)	doi:10.1016/j.biopha.2019.109111
						FBXW7, DKK3	glioma cells	—	oncomiR (promotes cell proliferation, metastasis)	doi:10.3892/etm.2019.7583
						BTG2	breast cancer	—	oncomiR (promotes cell proliferation, metastasis)	doi:10.1155/2019/7024675
						SEMA4C	cervical cancer	—	tumor suppressor (suppresses EMT)	doi:10.1111/cas.13104
						ADAM10	endothelial cells	—	inhibit NFkB Signaling and reduces inflammation	doi:10.3389/fimmu.2019.02205
						EZH2	cardiomyocytes	—	cardioprotective (inhibit cardiomyocyte apoptosis during injury)	doi:10.1080/0886022X.2020.1745236
TS/O	miR-23b-3p	0.73 ± 0.69	1.02 ± 0.26	–1.23	0.307825	SIRT1	lens epithelial cells	—	reduces apoptosis in oxidative stress	doi:10.1002/jcb.29270
						TGFBR3	atrial fibroblasts	miR-27b-3p	promote atrial fibrosis in atrial fibrillation	doi:10.1111/jcmm.14211
						CB1R	gastric cancer	miR-130a-5p (not detected)	tumor suppressor (cell proliferation)	doi:10.2147/OTT.S181706
						ANXA2	pancreatic ductal adenocarcinoma	—	tumor suppressor (cell proliferation)	doi:10.1159/000494468
						ETS1	hepatocytes	—	downregulate Apo(a) expression	doi:10.1002/cbin.10896
						PGC1A	osteosarcoma	—	oncomiR (promotes cell proliferation)	doi:10.1038/s41419-019-1614-1
						EBF3	squamous cell carcinoma	—	oncomiR (promotes cell proliferation, metastasis)	doi:10.1093/abbs/gmy049
						ZEB1	hepatocellular carcinoma	—	tumor suppressor (suppresses epithelial–mesenchymal transition)	doi:10.1016/j.gene.2018.05.061
						CMET	cervical cancer	—	tumor suppressor (cell proliferation, invasion, metastasis)	doi:10.1038/s41598-020-60143-x
						ATG12, HMGB2	gastric cancer	—	tumor suppressor (chemosensitivity)	doi:10.1038/cddis.2015.123
						HS6ST2	chondrocytes	—	enhances matrix degradation in osteoarthritis	doi:10.1038/s41419-018-0729-0
						TGIF1	keratinocytes	—	regulation of keratinocyte differentiation	doi:10.1111/exd.13119
						PTEN	renal cancer	—	oncomiR (promotes cell proliferation)	doi:10.1371/journal.pone.0050203
TS/O, CP	miR-27b-3p	2.97 ± 0.79	2.77 ± 0.5	1.15	0.84	TGFBR3	atrial fibroblasts	miR-27b-3p	promote atrial fibrosis in atrial fibrillation	doi:10.1111/jcmm.14211
						HOXA10	colorectal carcinoma	—	oncomiR (promotes cell invasion, metastasis)	doi:10.1042/BSR20191087
						CBLB, GRB2	breast cancer	—	tumor suppressor (cell proliferation, chemoresistance)	doi:10.1038/s41419-017-0211-4
						WNT3A	atrial fibroblasts	—	cardioprotection (reduces atrial fibrosis during atrial fibrillation)	doi:10.1155/2019/5703764
						MARCH7	endometrial carcinoma	—	tumor suppressor (cell proliferation, invasion, metastasis)	doi:10.1093/abbs/gmz030
						SMAD7	endothelial cells	—	suppresses endothelial cell proliferation and migration in Kawasaki disease	doi:10.1159/000492354
						HIPK2	chondrocytes	—	inhibits apoptosis in rheumatoid arthritis	doi:10.1080/21691401.2019.1607362
						PPARG	thyroid cancer	—	oncomiR (chemoresistance)	doi:10.1111/bcpt.13076
						FZD7	lung cancer	—	tumor suppressor (tumor progression)	PMID: 29028088
						SP7	maxillary sinus membrane stem cells	—	suppress osteogenic differentiation	doi:10.1097/ID.0000000000000637
						LIMK1	colorectal carcinoma	—	tumor suppressor (cell proliferation, invasion, metastasis)	PMID: 31966797
						GSPT1	gastric cancer	—	tumor suppressor (tumor progression)	doi:10.1016/j.biopha.2019.109417
						YAP1	glioma cells	—	tumor suppressor (tumorigenesis)	doi:10.1139/bcb-2019-0300
						ROR1	gastric cancer	—	tumor suppressor (cell proliferation)	doi:10.1186/s13046-015-0253-3
						PPARG	oocytes	—	maturation	doi:10.1016/j.bbrc.2016.09.046
						GSPT1	non-small cell lung carcinoma	—	tumor suppressor (cell proliferation, invasion, metastasis)	doi:10.2147/OTT.S196865
						NRF2	squamous cell carcinoma	—	tumor suppressor (tumor progression)	doi:10.1007/s13577-020-00329-7
						TRAF3	chondrocytes	—	inhibits IL1B-induced injury	doi:10.1016/j.intimp.2019.106052
						NR5A2, CREB1	breast cancer	—	tumor suppressor (chemosensitivity)	doi:10.1038/cddis.2016.361
TS/O, CP	miR-24-3p	0.43 ± 0.62	1.48 ± 0.56	–2.05	0.091	KEAP1	cardiomyocytes	—	cardioprotection (in ischemia-reperfusion injury)	doi:10.1155/2018/7042105
						FGF11	T-cells	—	oncomiR (immune evasion)	doi:10.1002/path.4781
						SOX7	lung cancer	—	oncomiR (promotes metastasis, invasion)	doi:10.1002/jcb.26553
						SOCS6	prostate cancer	—	oncomiR (promotes metastasis, invasion, proliferation)	PMID: 31938287
						p27KIP1	papillary thyroid carcinoma	—	oncomiR (promotes metastasis, invasion, proliferation)	doi:10.26355/eurrev_201907_18327
						BIM	breast cancer	—	oncomiR (chemoresistance)	doi:10.1002/jcb.28568
						RIPK1	cardiomyocytes	—	cardioprotection (in ischemia-reperfusion injury)	doi:10.1159/000495161
						DEDD	bladder cancer	—	oncomiR (promotes tumor progression)	doi:10.3892/or.2016.5326
						PRKCH	Lacrimal adenoid cystic carcinoma	—	tumor suppressor (tumor progression)	doi:10.1371/journal.pone.0158433
						MTT1	hepatocellular carcinoma	—	oncomiR (promotes cell proliferation)	doi:10.1002/cbf.3213
						SMAD5	peridontal stem cells	—	inhibit osteogenic differentiation	doi:10.1002/jcp.27499
						JAB1/CSN5	nasopharyngeal carcinoma	—	tumor suppressor (radiosensitivity)	doi:10.1038/onc.2016.147
						ATG4A	small cell lung cancer	—	tumor suppressor (chemosensitivity)	doi:10.18632/oncotarget.2787
						IGFBP5	intervertebrate discs	—	induces disc degeneration	doi:10.1016/j.lfs.2020.117288
						NOTCH1, DLL1	endothelial cells	—	inhibit angiogenesis after myocardial infarction	doi:10.3390/ijms21051733
						LAMB3	pancreatic ductal adenocarcinoma	—	tumor suppressor (tumor progression)	doi:10.3389/fonc.2019.01499
						CHD5	squamous cell carcinoma	—	oncomiR (promotes cell proliferation, chemoresistance)	doi:10.2217/fon-2016-0179
						FGF11	fibroblasts	—	activation of fibrosis and proliferation in renal fibrosis	doi:10.1002/jcp.29329
TS/O	miR-23a-3p	-0.14 ± 0.54	0.06 ± 0.95	–1.14	0.99	PNRC2	renal cell carcinoma	—	oncomiR (promotes tumor progression)	doi:10.1016/j.biopha.2018.11.065
						KLF3	melanoma	—	oncomiR (promotes tumor progression)	doi:10.1186/s12935-019-0927-6
						FGF2	squamous cell carcinoma	—	tumor suppressor (cell proliferation)	doi:10.1016/j.prp.2018.12.021
						CHD17	hepatocellular carcinoma	—	oncomiR (promotes cell proliferation)	doi:10.1007/s13105-020-00726-4
						SMAD3	chondrocytes	—	promotes osteoarthritis	doi:10.1016/j.bbrc.2016.06.071
						PTEN	gliomal cells	—	oncomiR (promotes cell proliferation)	doi:10.1002/ar.24410
TS/O, CP	miR-130a-3p	4.86 ± 0.83	5.01 ± 0.74	–1.11	0.75	PDE4D	cardiomyocytes	—	cardioprotection (improves cardiac cell proliferation after myocardial infarction)	doi:10.1002/jcp.26327
						SMAD4	esophageal cancer	—	oncomiR (promotes epithelial–mesenchymal transition)	doi:10.1002/cam4.1981
						RAB5B	breast cancer	—	tumor suppressor ( invasion, metastasis)	doi:10.1016/j.bbrc.2018.05.018
						SOX4	non-small cell lung carcinoma	—	tumor suppressor (chemosensitivity)	doi:10.1080/15384047.2017.1385679
						SMAD4	hepatoma cells	—	tumor suppressor (invasion, metastasis)	doi:10.1186/s13046-016-0296-0
						SNON	kidney	—	inhibition of renal fibrosis	doi:10.1016/j.yexmp.2019.104358
						TGFBR1/2	hepatic stellate cells	—	decreases hepatic fibrosis	doi:10.1038/cddis.2017.10
						BACH2	nasopharyngeal carcinoma	—	tumor suppressor (cell autophagy)	doi:10.1042/BSR20160576
TS/O	miR-15a-5p	5.52 ± 2.85	4.42 ± 1.07	2.14	0.73	VEGFA	chondrocytes	—	aggravates osteoarthritis	doi:10.5582/bst.2016.01187
						VEGFA	peritoneal mesothelial cells	—	suppresses inflammation and fibrosis	doi:10.1002/jcp.27660
						WNT3A	endometrial carcinoma	—	tumor suppressor (cell growth)	PMID: 29164582
						CXCL10	chronic myeloid leukemia	—	tumor suppressor (cell autophagy), metastasis	PMID: 28979704
						MYCN	neuroblastoma cells	miR-15b-5p, miR-16-5p	tumor suppressor (tumor progression)	doi:10.1002/1878-0261.12588
						PTHrP	chondrocytes	—	promotes osteoarthritis	doi:10.1155/2019/3904923
						FASN	arteries	—	alleviates atherosclerosis and vascular inflammation	doi:10.1042/BSR20181852
						PHLPP2	gastric cancer	—	oncomiR (chemoresistance)	doi:10.4149/neo_2020_190904N861
						TP53INP1	cervical cancer	—	oncomiR (anti-apoptotic)	doi:10.26355/eurrev_201910_19129
						BDNF	hepatocellular carcinoma	—	tumor suppressor (cell proliferation)	doi:10.1007/s13277-015-4427-6
						TGFB3, VEGF	retinal endothelial cells	—	promote endothelial cell tight junction formation	doi:10.1016/j.visres.2017.07.007
						VEGFA	endometrial mesenchymal stem cells	—	promote endometriosis	PMID: 27608888
						HOXA3	thyroid cancer	—	tumor suppressor (tumor progression)	doi:10.1089/hum.2018.109
TS/O, CP	miR-181a-5p	5.5 ± 0.68	6.54 ± 0.7	–1.09	0.9	PBX1	ligaments	—	promotes osteogenesis	doi:10.7150/thno.44309
						CBLB	esophageal cancer	—	tumor suppressor (chemosensitivity)	doi:10.2147/CMAR.S251264
						E2F7	non-small cell lung carcinoma	—	oncomiR (tumor progression)	doi:10.2147/CMAR.S240964
						ESM1	retina	—	anti-angiogenesis	doi:10.1002/jcp.29733
						SIRT1	cardiomyocytes	—	promotes apoptosis in hypoxic injury	doi:10.1080/09168451.2020.1750943
						KLF17	prostate cancer	—	oncomiR (promotes epithelial–mesenchymal transition)	PMID: 32195032
						PDGFRA	endothelial cells	—	anti-angiogenesis	doi:10.1002/cbf.3472
						AKT3	gastric adenocarcinoma	—	tumor suppressor (cell proliferation, apoptosis)	doi:10.1098/rsob.190095
						p53	cardiomyocytes	—	cardioprotection (reduces high glucose induced apoptosis)	doi:10.1538/expanim.19-0058
						ATG7	hepatocellular carcinoma	—	oncomiR (inhibits autophagy)	doi:10.1002/jcb.29064
TS/O	miR-106a-5p	5.19 ± 0.76	5.42 ± 1.07	–1.17	0.97	HK2	squamous cell carcinoma	—	tumor suppressor (cell proliferation, invasion, metastasis)	doi:10.1007/s11010-020-03840-5
						STAT3	endothelial cells	—	allelviates atherosclerosis and vascular inflammation	doi:10.3892/mmr.2020.11147
						RBM24	prostate cancer	—	oncomiR (tumor progression)	doi:10.2147/OTT.S246274
						TGFBR2	colorectal carcinoma	—	oncomiR (chemoresistance)	PMID: 31949649
						ARHGAP24	ovarian carcinoma	—	oncomiR (cell proliferation, invasion)	doi:10.1016/j.lfs.2020.117296
						TGFBR2	palate	—	promotes cleft palate formation	doi:10.1016/j.yexcr.2019
TS/O	miR-125a-5p	2.3 ± 0.54	2.64 ± 0.22	–1.27	0.27	FUT4	osteosarcoma	—	tumor suppressor (tumor progression)	doi:10.3389/fgene.2020.00672
						LIN28B	ovarian carcinoma	—	tumor suppressor (cell proliferation, metastasis)	doi:10.3892/mmr.2020.11223
						MACC1	hepatocellular carcinoma	miR-34a	tumor suppressor (cell proliferation, metastasis)	doi:10.4149/neo_2020_191019N1062
						FNDC3B	colorectal carcinoma	miR-217	oncomiR (cell proliferation, invasion)	doi:10.2147/OTT.S226520
						HK2	lung	—	inhibits glycolysis and improved pulmonary arterial hypertension	doi:10.18632/aging.103163
						TRAF6	macrophages	—	promotes M2 polarization	doi:10.1007/s10753-020-01231-y
						GALNT7	cervical cancer	—	tumor suppressor (cell proliferation, invasion)	doi:10.1186/s12935-020-01209-8
						VEGFA	trophoblast	—	suppresses migration and proliferation	doi:10.1016/j.bbrc.2020.02.137
						GAB2	breast cancer	—	tumor suppressor (cell proliferation, invasion)	doi:10.3934/mbe.2019347
						SIRT7	non-small cell lung carcinoma	—	tumor suppressor (radioresistance)	doi:10.3233/CBM-190381
						TAZ	ovarian carcinoma	—	tumor suppressor (inhibits epithelial–mesenchymal transition)	doi:10.3233/CBM-190381
TS/O, CP	miR-125b-5p	−1.36 ± 0.51	−1.21 ± 0.36	–1.11	0.58	p53, BAK1	cardiomyocytes	—	cardioprotection (inhibits apoptosis in ischemia-reperfusion injury)	doi:10.7150/thno.28021
						p53, BNIP3	cardiomyocytes	—	cardioprotection (inhibits apoptosis in ischemia-reperfusion injury)	doi:10.1161/CIRCRESAHA.118.312758
						SMAD7	cardiomyocytes	—	cardiotoxic (increase hypoxia induced injury signaling)	doi:10.3892/ijmm.2018.3496
						BAK1, KLF13	cardiomyocytes	—	cardioprotection (inhibits apoptosis in ischemia-reperfusion injury)	doi:10.1016/j.yjmcc.2017.11.003
						EIF5A2	melanoma	—	tumor suppressor (cell proliferation, metastasis)	doi:10.1186/s13046-020-01599-7
						BTG2	lung adenocarcinoma	—	oncomiR (cell proliferation, migration and promotes epithelial–mesenchymal transition)	doi:10.26355/eurrev_202004_20841
						PAK3	prenatal follicles	—	inhibits steroidogenesis	doi:10.1016/j.metabol.2020.154241
						BACE1	neurons	—	attenuate neurotoxicity	doi:10.1016/j.jns.2020.116793
						TRIB2	squamous cell carcinoma	—	tumor suppressor (tumor progression)	doi:10.1042/BSR20193172
						PDK1	cervical cancer	—	tumor suppressor (tumorigenesis)	doi:10.1155/2020/4351671
						NLRC5	cardiomyocytes	—	cardioprotection (inhibits apoptosis in ischemia-reperfusion injury)	doi:10.3892/etm.2019.8309
						STAT3	embryonic stem cells	—	tumor suppressor (tumorigenesis)	doi:10.7150/jca.33696
						TRAF6	skeletal muscle	—	relieves skeletal muscle atrophy	doi:10.21037/atm.2019.08.39
						HK2	bladder cancer	—	tumor suppressor (tumor progression)	doi:10.1007/s13577-019-00285-x
						AKT3	keratinocytes	miR-181b-5p (not tested)	inhibit proliferation	doi:10.1016/j.ejphar.2019.172659
						LIMK1	brain	—	neuroprotection	doi:10.2174/1567202616666190906145936
						TXNRD1	hepatocellular carcinoma	—	tumor suppressor (cell proliferation, invasion, metastasis)	doi:10.1186/s12935-019-0919-6
						TRAF6	chondrocytes	—	anti-inflammatory in the setting of osteoarthritis	doi:10.1038/s41598-019-42601-3
TS/O, CP	miR-19a-3p	3.07 ± 0.62	3.29 ± 1.04	–1.16	0.99	PTEN	brain	—	alleviates ischemia-reperfusion injury-induced apoptosis	doi:10.1016/j.neuroscience.2020.04.020
						IGFBP3	brain	—	alleviates ischemia-reperfusion injury	doi:10.1186/s40659-020-00280-9
						FAS	rectal cancer	—	tumor suppressor (induces apoptosis)	doi:10.1177/1533033820917978
						PIK3IP1	hepatocellular carcinoma	—	tumor suppressor (cell proliferation)	doi:10.7150/jca.37748
						FOXF2	colorectal carcinoma	—	tumor suppressor (inhibits epithelial–mesenchymal transition)	doi:10.3748/wjg.v26.i6.627
						IGFBP3	ovarian carcinoma	—	oncomiR (tumor progression)	doi:10.1002/mc.23113
						SOCS3	synovial cells	—	promote cell proliferation	10.1002/jcb.28442
						PTEN	osteosarcoma	—	oncomiR (chemoresistance)	doi:10.3892/ol.2018.9592
						PFN1	hepatocellular carcinoma	—	oncomiR (tumor progression)	doi:10.1016/j.prp.2018.12.012
						PTEN	hepatocellular carcinoma	—	oncomiR (chemoresistance, metastasis)	doi:10.1016/j.biopha.2018.06.097
						PITX1	gastric cancer	—	oncomiR (tumor progression)	doi:10.1159/000489590
						TSC1	osteoblasts	—	mediates dexamethasone resistance	doi:10.18632/oncotarget.23326
						SMAD2/4	prostate cancer	—	tumor suppressor (invasion, metastasis)	doi:10.3892/or.2017.6096
						TGFBR2	cardiac fibroblasts	miR-19b-3p	cardioprotection: anti-fibrotic	doi:10.1038/srep24747
TS/O, CP	miR-19b-3p	3.29 ± 0.35	3.07 ± 0.81	1.16	0.47	NRP1	gastric cancer	—	tumor suppressor (tumor progression)	doi:10.1186/s12935-020-01257-0
						CCDC6	cholangiosarcoma	—	oncomiR (promotes proliferation, epithelial–mesenchymal transition)	doi:10.1016/j.abb.2020.108367
						HIF1A	endothelial cells	—	anti-angiogenic after hypoxia	doi:10.1096/fj.201902434R
						BACE1	brain	miR-16-5p	prevent amyloid beta induced apoptosis	doi:10.1097/WNR.0000000000001379
						TNFAIP3	endothelial cells	—	pro-inflammatory in the setting of meningitis	doi:10.3390/pathogens8040268
						HOXA9	non-small cell lung carcinoma	—	oncomiR (promotes proliferation, migration, invasion)	doi:10.2147/OTT.S216320
						PTEN	pancreatic cancer	—	oncomiR (cell proliferation)	doi:10.21037/atm.2019.04.61
						GRK6	chondrocytes	—	reduces inflammation and matrix degradation	doi:10.1007/s11010-019-03563-2
						PTEN	muscle cells	—	osteogenic differentiation	doi:10.1002/cbin.11133
TS/O CP	miR-214-3p	2.19 ± 1.04	1.96 ± 0.59	1.17	0.99	PTEN	cardiomyocytes	—	cardioprotection: inhibiting autophagy in sepsis	doi:10.1155/2020/1409038
						ATM	lung	—	reduce radiation induced pulmonary injury	doi:10.1089/ars.2019.7965
						CENPM	hepatocellular carcinoma	—	tumor supressor (tumor progression)	doi:10.1093/jb/mvaa073
						PLAGL2	colorectal carcinoma	—	tumor suppressor (cell proliferation)	doi:10.18632/aging.103233
						LIVIN	colorectal carcinoma	—	tumor supressor (tumor progression)	doi:10.1080/21655979.2020
						IL17	myocardium	—	cardioprotective (anti-fibrotic)	doi:10.3389/fcell.2020.00243
						WNT23	vascular smooth muscle cells	—	inhibits cell proliferation	doi:10.26355/eurrev_202003_20696
						ABCB1, XIAP	retinoblastoma	—	oncomiR (chemoresistance)	doi:10.2147/OTT.S235862
						PSMD10	papillary thyroid carcinoma	—	tumor suppressor (tumor progression)	doi:10.1002/jcp.29557
						TWIST1	endometrial carcinoma	—	tumor suppressor (inhibit epithelial–mesenchymal transition)	doi:10.2147/OTT.S181037
						LHX6	ovarian carcinoma	—	oncomiR (tumorigenesis)	doi:10.3390/cancers11121917
						ST6GAL1	breast cancer	—	oncomiR (cell proliferation, inhibit apoptosis)	doi:10.1007/s10616-019-00352-z
						FOXP3	breast cancer	—	oncomiR (cell proliferation)	doi:10.26355/eurrev_201910_19156
						HDGF	pancreatic cancer	—	tumor suppressor (chemosensitivity)	doi:10.2147/OTT.S222703
						BIRC5	breast cancer	—	tumor suppressor (cell proliferation)	doi:10.26355/eurrev_201909_18856
						NLRC5	myocardium	—	cardioprotection: anti-fibrotic	doi:10.1042/CS20190203
						CTNNB1	preadipocytes	—	promote differentiation	doi:10.3390/ijms20081816
TS/O	miR-222-3p	2.34 ± 1.60	1.61 ± 0.57	1.66	0.50	PUMA	non-small cell lung carcinoma	—	oncomiR (cell proliferation, inhibit apoptosis)	doi:10.1177/1533033820922558
						PDCD10	ovarian carcinoma	—	tumor suppressor (inhibit epithelial–mesenchymal transition)	doi:10.7150/thno.43198
						GILZ	airway epithelial cells	—	ameliorates glucocorticoid induced inhibition of cell repair	doi:10.1080/10799893.2020.1742739
						IGF1	bone marrow-derived MSCs	—	promote osteogenic differentiation	doi:10.1016/j.diabres.2020.108121
						TMP2	renal clear cell carcinoma	—	oncomiR (tumor progression)	doi:10.3233/CBM-190264
						IRF2, INPP4B	acute myeloid leukemia	—	tumor suppressor (cell proliferation)	doi:10.1016/j.mcp.2020.101513
						CDKN1B	squamous cell carcinoma	—	oncomiR (tumorigenesis)	doi:10.1111/jop.12986
						PPP2R2A	large B-cell lymphoma	—	oncomiR (cell proliferation, inhibit apoptosis)	doi:10.1177/1533033819892256
						GAS5, PTEN	colorectal carcinoma	—	oncomiR (promotes cell proliferation, migration, invasion)	doi:10.1016/j.omtn.2019.06.009
						PDE3A	endothelial cells	miR-27a-3p	promote vascular integrity	doi:10.1007/s12035-018-1446-5
						TIMP3	osteosarcoma	—	oncomiR (promote metastasis and invasion)	doi:10.2147/OTT.S175745
						PTEN	papillary thyroid carcinoma	—	oncomiR (inhibit apoptosis)	doi:10.18632/oncotarget.23336
TS/O, CP	miR-26a-5p	3.75 ± 0.54	4.06 ± 0.72	–1.24	0.53	RANBP9	brain	—	inhibit injury induced apoptosis	doi:10.1016/j.acthis.2020.151571
						TLR4	kidney	—	protect against diabetic nephropathy	doi:10.1074/jbc.RA120.012522
						HMGA2	hepatocellular carcinoma	—	tumor suppressor (cell proliferation, promote apoptosis)	doi:10.2147/CMAR.S237752
						CTGF	macrophages	—	modulates TLR signaling upon activation	doi:10.1042/BSR20192598
						CREB1	renal cell carcinoma	miR-27a-3p, miR-221-3p	tumor suppressor (cell proliferation, promote apoptosis)	doi:10.1038/s41598-020-63403-y
						DYRK1A	brain	—	inhibit development Alzheimer’s disease	doi:10.2174/1567202617666200414142637
						WNT5A	gastric cancer	—	tumor suppressor (cell proliferation)	doi:10.2147/OTT.S241199
						ADAM17	cardiomyocytes	—	cardioprotection (inhibit apoptosis)	doi:10.1007/s10863-020-09829-5
						COL10A1	gastric cancer	—	tumor suppressor (cell proliferation, migration, and invasion)	doi:10.26355/eurrev_202002_20170
						HOXA5	osteosarcoma	—	oncomiR (promotes cell proliferation, migration)	doi:10.2147/OTT.S232100
						PTEN	myocardium	—	cardioprotection (inhibits apoptosis in ischemia-reperfusion injury)	doi:10.1590/1414-431 × 20199106
						PTGS2	joints	—	alleviate osteoarthritis	doi:10.1016/j.intimp.2019.105946
						AURKA	hepatocellular carcinoma	—	tumor suppressor (chemosensitivity)	doi:10.1177/1533033819851833
						WNT5A	papillary thyroid carcinoma	—	tumor suppressor (cell proliferation, migration, and invasion)	doi:10.2147/OTT.S205994
						PTEN	myocardium	—	cardioprotection (inhibits apoptosis in ischemia-reperfusion injury)	doi:10.26355/eurrev_201908_18661
						PTEN	synovial cells	—	promote cell proliferation and inhibit apoptosis	doi:10.1042/BSR20182192
TS/O, CP	miR-27a-3p	1.87 ± 0.98	1.80 ± 0.88	1.05	0.96	SLIT2	endothelial cells	—	promotes apoptosis, autophagy during inflammation	doi:10.1016/j.jss.2020.05.102
						SP7	preosteoblasts	—	promotes differentiation	doi:10.3892/mmr.2020.11246
						TAB3	kidney	—	promotes apoptosis during kidney injury	doi:10.1080/09168451.2020.1792760
						PDL1	macrophages	—	oncomiR (promotes immune evasion of breast cancer)	doi:10.1111/jcmm.15367
						BNIP3	pancreatic cancer	—	oncomiR (inhibits apoptosis)	doi:10.3892/ijmm.2020.4632
						SMURF2	lung	—	anti-fibrotic after bleomycin exposure	PMID: 32538751
						TGFBR1	cardiomyocytes	—	cardioprotection (inhibits apoptosis in ischemia-reperfusion injury)	doi:10.1155/2020/2016259
						FBXW7	cervical cancer	—	oncomiR (tumor progression)	doi:10.2147/CMAR.S234897
						ICOS	lung adenocarcinoma	—	tumor suppressor (promotes antitumor immunity)	doi:10.1111/1759-7714.13411
						NOVA	gastric cancer	—	oncomiR (promotes epithelial–mesenchymal transition)	doi:10.3892/mmr.2020.10949
						BNIP3	cardiomyocytes	—	cardioprotection (inhibits apoptosis in ischemia-reperfusion injury)	doi:10.1016/j.omtn.2019.11.017
TS/O, CP	miR-29a-3p	2.50 ± 0.87	1.64 ± 0.66	1.82	0.19	E2F1	ovarian carcinoma	—	oncomiR (promotes epithelial–mesenchymal transition)	doi:10.18632/aging.103388
						PTEN	aorta	—	promotes development of aortic aneurysms	doi:10.1002/jcp.29746
						DRP1	myocardium	—	cardioprotection (prevent myocardial hypertrophy)	doi:10.2174/0929866527666200416144459
						COL4A2	hepatocellular carcinoma	—	tumor suppressor (cell proliferation, migration, and invasion)	doi:10.1039/c9mt00266a
						COL5A1	breast cancer	—	tumor suppressor (cell proliferation, migration)	doi:10.1016/j.lfs.2019.117179
						TNFR1	endothelial cells	—	reduces TNF-alpha injury response	doi:10.1016/j.omtn.2019.10.014
TS/O, CP	miR-30b-5p	5.04 ± 1.10	4.56 ± 0.62	1.40	0.62	KIF18A	prostate cancer	—	oncomiR (radioresistance)	doi:10.1089/cbr.2019.3538.
						MYBL2	medulloblastoma	—	tumor suppressor (cell proliferation, promotes apoptosis)	doi:10.1136/jim-2020-001354
						CAMK2D	dermal papilla cells	—	inhibits proliferation	doi:10.1186/s12864-020-06799-1
						ASPP2	breast cancer	—	oncomiR (cell proliferation, migration, and invasion)	doi:10.1155/2020/7907269
						PTAFR	myocardium	—	cardioprotection: anti-fibrotic	doi:10.26355/eurrev_202004_20869
						PPARGC1A	Huh-7 cells	—	regulate lipid metabolism	doi:10.1186/s12944-020-01261-3
						CTNNB1	cardiomyocytes	—	cardiotoxic (increased apoptosis during myocardial injury)	doi:10.23736/S00264806.20.06565-9
						AVEN	cardiomyocytes	—	cardiotoxic (increased apoptosis during myocardial injury)	doi:10.1186/s11658-019-0187-4
TS/O, CP	miR-31-5p	1.42 ± 0.46	2.24 ± 0.74	–1.76	0.15	YAP	colorectal carcinoma	—	tumor suppressor (cell proliferation, metastasis, chemosensitivity)	doi:10.1016/j.yexcr.2020.112176
						FLOT1	renal clear cell carcinoma	—	tumor suppressor (cell proliferation, promote apoptosis)	doi:10.2147/OTT.S254634
						HOXA7	trophoblast	—	inhibit proliferation	doi:10.1111/jog.14344
						PEX5	hepatocellular carcinoma	—	oncomiR (radioresistance)	doi:10.7150/thno.42371
						TNS1	colon adenocarcinoma	—	oncomiR (tumor progression)	doi:10.18632/aging.103096
						PKCG	cardiomyocytes	—	cardioprotective, inhibit cardiomyocyte hypertrophy	doi:10.26355/eurrev_202002_20351
						PAN3	cardiomyocytes	—	cardioprotective: attenuates doxorubicin induced cardiotoxicity	doi:10.1016/j.yjmcc.2020.02.009
						ETBR, VEGFA	endothelial cells	—	anti-angiogenic	doi:10.1016/j.lfs.2020.117306
						MEGEA3	hepatocellular carcinoma	—	oncomiR (chemoresistance, cell proliferation)	doi:10.1016/j.omtn.2019.10.035
						LATS2	colorectal carcinoma	—	oncomiR (chemoresistance)	doi:10.3390/cancers11101576
						MLH1	renal cell carcinoma	—	oncomiR (chemoresistance)	doi:10.1002/ijc.32543
						VEGFA	gliomal cells	—	tumor suppressor (anti-angiogenic)	doi:10.1002/ijc.32483
TS/O	miR-365a-3p	3.99 ± 0.84	4.34 ± 0.67	–1.27	0.43	ABCC4	gastric cancer	—	tumor suppressor (tumor progression)	doi:10.2147/OTT.S245557
						ADAM10	colorectal carcinoma	—	tumor suppressor (cell proliferation, migration)	doi:10.7150/jca.42731
						CREL	pancreatic cancer	—	tumor suppressor (tumor progression)	doi:10.1016/j.canlet.2019.03.025
						TET1	hepatocellular carcinoma	—	tumor suppressor (tumor progression, invasion)	doi:10.4149/neo_2018_171119N752
						USP33	lung cancer	—	oncomiR (tumorigenesis)	doi:10.1186/s12935-018-0563-6
TS/O	miR-93-5p	5.04 ± 0.59	5.43 ± 0.62	–1.31	0.39	MAP3K2	hepatocellular carcinoma	—	oncomiR (tumor progression)	doi:10.1038/s41388-020-01401-0
						RGMB	squamous cell carcinoma	—	oncomiR (migration and invasion)	doi:10.7150/jca.43854
						FOXA1	colorectal carcinoma	—	oncomiR (radioresistance)	doi:10.1186/s13046-019-1507-2
						AHNAK	gastric cancer	—	oncomiR (promotes epithelial–mesenchymal transition)	doi:10.1186/s12935-019-1092-7
						PD-L1	colorectal carcinoma	—	tumor suppressor (tumor progression)	doi:10.1002/cbin.11323
						FOXK2	cervical cancer	—	oncomiR (tumor progression)	doi:10.1007/s43032-020-00140-7
						CASC2	chondrocytes	—	inhibits apoptosis in osteoarthritis	doi:10.1186/s12891-019-3025-y
						MMP2	gliomal cells	—	tumor suppressor (cell proliferation, migration)	doi:10.26355/eurrev_201911_19446
TS/O	let-7a-5p	2.46 ± 0.96	2.79 ± 0.46	–1.26	0.40	SMAD2	chondrocytes	—	promotes hypertrophic differentiation	doi:10.1152/ajpcell.00039.2020
						SAMD2	lens epithelial cells	—	inhibits proliferation, migration and invasion	PMID: 32345785
						DUSP7	breast cancer	—	tumor suppressor (chemoresistance)	doi:10.2147/CMAR.S238513
						BCLXL	lung cancer	—	tumor suppressor (cell autophagy)	doi:10.1016/j.omto.2019.08.010
						BCL2L1	lung cancer	—	tumor suppressor (induce apoptosis)	doi:10.3389/fonc.2019.00808
						EGFR	breast cancer	—	oncomiR (chemoresistance)	doi:10.1002/iub.2075
						HMGA2	kidney	—	promotes diabetic nephropathy	doi:10.3892/mmr.2019.10057
