# Supplementary material for: MiRNA Profiles of Extracellular Vesicles Secreted by Mesenchymal Stromal Cells—Can They Predict Potential Off-Target Effects?
Source: Biomolecules. 2020 Sep 22;10(9):1353. doi: 10.3390/biom10091353 (PMC7565205; doi:10.3390/biom10091353)
Supplement: Supplementary file 1 [file biomolecules-10-01353-s001.pdf]

Table S1. Analyzed miRNAs and their expression values. EV samples isolated from CB-MSCs are indicated as CB_MSC_1-4 and EV samples isolated from AT-MSCs are indicated as AT_MSC_1-4.

MiRNA ID	Reference	CTcorr value							
		CB_MSC_1	CB_MSC_2	CB_MSC_3	CB_MSC_4	AT_MSC_1	AT_MSC_2	AT_MSC_3	AT_MSC_4
hsa-miR-7-5p	YP00205877	32,23	35,00	33,98	35,00	35,00	34,47	35,00	34,05
hsa-miR-217	YP00204010	35,00	35,00	35,00	35,00	35,00	35,00	35,00	35,00
hsa-miR-337-5p	YP00204391	30,34	34,02	31,99	31,72	32,92	32,71	33,96	34,19
hsa-miR-328-3p	YP00204364	29,41	32,04	30,74	31,64	31,42	31,66	32,45	32,65
hsa-miR-374b-3p	YP00205917	35,00	35,00	35,00	35,00	35,00	35,00	35,00	35,00
hsa-miR-143-3p	YP00205992	25,62	28,80	27,53	29,06	28,71	26,62	28,54	27,87
hsa-miR-623	YP00204019	35,00	35,00	35,00	35,00	35,00	35,00	35,00	35,00
hsa-miR-520c-3p	YP00204497	35,00	35,00	35,00	35,00	35,00	35,00	35,00	35,00
hsa-miR-557	YP00204346	35,00	35,00	35,00	35,00	35,00	35,00	35,00	35,00
hsa-miR-218-5p	YP00206034	31,02	35,00	33,11	34,61	31,70	29,71	31,85	30,39
hsa-miR-136-5p	YP00204779	27,71	30,00	28,71	28,37	30,71	28,95	31,77	29,52
hsa-miR-127-5p	YP00204161	30,89	34,28	32,79	32,68	32,70	31,88	34,48	33,38
hsa-miR-140-5p	YP00204540	30,00	29,87	31,03	33,13	32,07	30,88	33,38	31,52
hsa-miR-31-3p	YP00204079	27,77	29,75	29,02	31,07	29,74	28,83	30,05	29,52
hsa-miR-20b-3p	YP00204569	35,00	35,00	35,00	35,00	35,00	35,00	35,00	35,00
hsa-miR-325	YP00204464	35,00	35,00	35,00	35,00	35,00	35,00	35,00	35,00
hsa-miR-509-3-5p	YP00204503	35,00	35,00	35,00	35,00	35,00	35,00	35,00	35,00
hsa-miR-210-3p	YP00204333	29,61	31,33	32,66	29,99	29,31	28,49	30,74	29,37
hsa-miR-199b-5p	YP00204152	26,58	31,90	28,18	28,77	28,22	26,99	29,44	28,08
hsa-miR-194-5p	YP00204080	32,90	35,00	35,00	35,00	34,46	32,64	35,00	35,00
hsa-let-7g-5p	YP00204565	26,94	30,01	28,05	31,64	29,38	27,88	30,21	28,74
hsa-miR-203a-3p	YP00205914	35,00	35,00	35,00	35,00	35,00	34,39	35,00	35,00
hsa-miR-181a-3p	YP00204110	34,04	34,44	35,00	35,00	35,00	33,38	35,00	33,94
hsa-miR-137	YP00206062	29,14	30,98	29,84	33,53	32,22	30,92	33,54	31,54
hsa-miR-551b-3p	YP00204067	33,65	35,00	35,00	34,15	35,00	35,00	35,00	35,00
hsa-miR-524-3p	YP00204030	35,00	35,00	35,00	35,00	35,00	35,00	35,00	35,00
hsa-miR-486-5p	YP00204001	31,83	35,00	32,02	32,10	34,38	35,00	35,00	35,00
hsa-miR-329-3p	YP00206052	31,35	33,19	33,08	33,46	35,00	33,56	35,00	33,68
hsa-miR-487b-3p	YP00204489	30,89	32,74	31,82	32,67	33,11	32,20	35,00	33,44
hsa-miR-138-5p	YP00206078	30,78	31,35	31,89	27,59	27,39	26,57	29,77	29,51
hsa-miR-191-5p	YP00204306	26,13	28,68	27,99	28,09	28,23	27,77	29,47	29,38
hsa-miR-378a-3p	YP00205946	33,63	35,00	35,00	35,00	35,00	30,53	32,25	33,56
hsa-miR-103a-3p	YP00204063	24,68	28,44	27,04	27,69	28,97	27,21	29,61	28,98
hsa-miR-890	YP00205859	35,00	35,00	35,00	35,00	35,00	35,00	35,00	35,00
hsa-miR-423-5p	YP00205624	28,86	32,39	30,40	29,91	29,79	29,68	30,06	31,42
hsa-miR-221-3p	YP00204532	23,94	26,49	24,91	27,52	24,95	24,01	25,72	25,75
SNORD38B (hsa)	YP00203901	29,21	30,21	26,93	28,55	31,98	28,68	29,51	28,99
hsa-miR-301b	YP00204390	34,24	35,00	35,00	35,00	35,00	35,00	35,00	35,00
SNORD49A (hsa)	YP00203904	32,53	33,90	30,57	32,28	33,34	30,14	30,87	31,16
hsa-miR-550a-5p	YP00204638	34,63	35,00	35,00	35,00	35,00	35,00	35,00	35,00
U6 snRNA (hsa, mmu)	YP00203907	35,00	35,00	33,10	31,92	35,00	32,63	30,40	32,12
hsa-miR-532-5p	YP00204221	31,65	33,73	34,40	35,00	33,69	31,91	33,75	33,60

hsa-miR-99a-5p	YP00204521	27,85	28,92	29,09	28,46	28,77	27,01	29,76	29,54
hsa-miR-16-5p	YP00205702	24,88	27,60	25,76	26,67	26,26	25,30	27,18	26,30
hsa-miR-98-5p	YP00204640	30,88	33,14	31,80	35,00	32,97	31,81	33,88	33,32
hsa-miR-185-5p	YP00206037	29,03	31,81	30,10	29,92	28,35	29,33	31,57	30,47
hsa-miR-25-3p	YP00204361	27,70	30,32	28,82	29,10	29,42	27,82	30,18	29,58
hsa-miR-765	YP00204071	35,00	35,00	35,00	35,00	35,00	35,00	35,00	35,00
hsa-miR-24-3p	YP00204260	23,69	25,76	25,28	24,90	25,29	25,04	26,57	26,82
hsa-miR-369-5p	YP00206014	33,24	35,00	33,37	33,76	34,42	33,59	35,00	35,00
hsa-miR-425-5p	YP00204337	29,36	31,21	30,76	31,12	30,90	29,93	30,69	31,69
hsa-miR-590-5p	YP00204222	31,83	33,86	34,27	35,00	33,77	32,65	35,00	32,44
hsa-miR-760	YP00204549	35,00	35,00	35,00	35,00	35,00	35,00	35,00	35,00
hsa-miR-574-3p	YP00206011	27,57	30,35	29,47	35,00	31,25	28,83	31,38	30,69
hsa-miR-130b-3p	YP00204317	30,81	32,98	30,79	31,71	32,12	30,92	32,94	31,46
hsa-miR-30c-5p	YP00204783	26,65	28,87	28,44	29,96	29,84	28,10	29,78	29,44
hsa-miR-133b	YP00206058	33,82	35,00	35,00	35,00	35,00	35,00	35,00	35,00
hsa-miR-524-5p	YP00204135	35,00	35,00	35,00	35,00	35,00	35,00	35,00	35,00
hsa-miR-23a-3p	YP00204772	22,48	25,17	24,85	24,82	24,87	23,98	23,94	25,27
hsa-miR-193b-3p	YP00204226	26,47	28,13	27,87	28,05	26,76	25,40	27,23	27,25
hsa-miR-501-5p	YP00204648	33,72	33,65	34,43	34,02	34,25	31,81	33,89	33,51
hsa-miR-518c-5p	YP00204280	35,00	35,00	35,00	35,00	35,00	35,00	35,00	35,00
hsa-miR-130a-3p	YP00204658	26,97	31,15	28,72	30,46	29,23	28,74	30,87	29,02
hsa-miR-933	YP00205996	35,00	35,00	35,00	35,00	35,00	35,00	35,00	35,00
hsa-miR-379-5p	YP00205658	30,63	32,59	31,69	34,09	34,05	31,97	34,46	33,40
hsa-miR-452-5p	YP00204301	31,48	33,36	34,38	33,78	33,00	32,76	34,65	34,00
hsa-miR-589-5p	YP00205675	35,00	35,00	35,00	35,00	35,00	35,00	35,00	35,00
hsa-miR-141-3p	YP00204504	35,00	35,00	35,00	35,00	35,00	35,00	35,00	35,00
hsa-miR-342-3p	YP00205625	28,84	31,61	29,87	28,91	30,88	29,71	30,97	31,47
hsa-miR-668-3p	YP00204206	35,00	35,00	35,00	35,00	35,00	35,00	35,00	35,00
hsa-miR-934	YP02119292	35,00	35,00	35,00	35,00	35,00	35,00	35,00	35,00
hsa-miR-101-3p	YP00204786	29,68	33,75	31,96	32,16	32,10	31,37	33,41	32,32
hsa-miR-539-5p	YP00205656	33,77	35,00	32,35	35,00	35,00	34,01	35,00	35,00
hsa-miR-331-3p	YP00206046	29,56	31,35	30,40	34,51	32,50	30,66	32,78	32,31
hsa-miR-499a-5p	YP00205935	35,00	35,00	35,00	35,00	35,00	35,00	35,00	35,00
hsa-miR-196a-5p	YP00204386	35,00	35,00	35,00	35,00	32,33	31,01	33,23	32,37
hsa-miR-888-5p	YP00206024	35,00	35,00	35,00	35,00	35,00	35,00	35,00	35,00
hsa-miR-330-3p	YP00204017	32,05	35,00	33,97	35,00	35,00	35,00	35,00	35,00
hsa-miR-570-3p	YP00204694	35,00	32,97	35,00	35,00	32,11	35,00	35,00	35,00
hsa-miR-518c-3p	YP00204554	35,00	35,00	35,00	35,00	35,00	35,00	35,00	35,00
hsa-miR-200a-3p	YP00204707	35,00	35,00	35,00	35,00	35,00	35,00	35,00	35,00
hsa-miR-188-5p	YP00204239	33,84	35,00	35,00	35,00	33,63	32,90	35,00	34,31
hsa-miR-26a-5p	YP00206023	26,79	29,77	27,47	28,83	28,24	28,11	29,46	28,25
hsa-miR-99b-5p	YP00205983	27,39	29,20	28,70	30,37	29,83	28,62	30,59	30,59
hsa-miR-431-5p	YP00204737	30,76	32,75	32,71	33,48	32,45	31,61	33,60	33,73
hsa-miR-23b-3p	YP00204790	23,55	25,38	24,83	27,03	25,95	23,98	26,13	25,85
hsa-miR-367-3p	YP00204784	35,00	35,00	35,00	35,00	35,00	35,00	35,00	35,00
hsa-miR-505-3p	YP00204214	30,48	32,69	32,08	33,93	33,28	32,62	35,00	33,51

hsa-miR-18a-5p	YP00204207	30,53	32,81	31,82	32,05	33,11	32,49	34,29	31,27
hsa-miR-92a-3p	YP00204258	26,86	28,37	27,48	27,36	27,80	26,42	27,99	28,07
hsa-miR-500a	YP00204794	32,87	35,00	33,90	35,00	35,00	35,00	35,00	35,00
hsa-miR-887-3p	YP00204472	32,00	33,64	33,40	34,56	33,05	31,82	35,00	31,63
hsa-miR-491-3p	YP00205977	35,00	35,00	35,00	30,87	35,00	35,00	35,00	35,00
hsa-miR-423-3p	YP00204488	27,55	29,86	28,78	29,64	29,09	27,62	29,52	29,47
hsa-miR-126-3p	YP00204227	34,44	34,18	34,23	35,00	35,00	32,95	35,00	35,00
hsa-miR-421	YP00204603	35,00	35,00	35,00	35,00	35,00	35,00	35,00	35,00
hsa-miR-376b-3p	YP00204218	29,83	33,36	32,02	32,00	33,01	32,02	33,89	32,44
hsa-miR-302c-3p	YP00204403	35,00	35,00	35,00	35,00	35,00	35,00	35,00	35,00
hsa-miR-625-3p	YP00204647	35,00	33,84	33,06	35,00	34,22	31,23	35,00	33,99
hsa-miR-339-5p	YP00206007	29,68	32,24	31,08	32,70	30,80	29,99	30,64	31,36
hsa-miR-873-5p	YP00204175	35,00	35,00	35,00	35,00	35,00	35,00	35,00	35,00
hsa-miR-323a-3p	YP00204278	30,74	32,66	31,61	30,81	32,96	32,38	35,00	35,00
hsa-miR-181d-5p	YP00204789	33,74	35,00	34,68	35,00	35,00	35,00	35,00	35,00
hsa-miR-125a-5p	YP00204339	25,76	27,38	26,96	26,96	27,34	25,98	27,65	27,42
hsa-miR-129-5p	YP00204534	35,00	35,00	35,00	35,00	35,00	35,00	35,00	35,00
hsa-miR-492	YP00204053	35,00	35,00	35,00	35,00	35,00	35,00	35,00	35,00
hsa-miR-20a-5p	YP00204292	27,63	30,51	28,93	30,14	29,91	27,68	30,73	28,38
hsa-miR-374b-5p	YP00204608	29,86	32,12	30,44	32,99	32,00	30,55	32,54	31,74
hsa-miR-302d-3p	YP00204311	35,00	35,00	35,00	35,00	35,00	35,00	35,00	35,00
hsa-miR-346	YP00206009	35,00	35,00	35,00	35,00	35,00	35,00	35,00	35,00
hsa-miR-151a-3p	YP00204576	29,06	31,07	30,68	32,08	30,95	30,49	32,10	30,26
hsa-miR-493-3p	YP00204557	30,46	35,00	33,03	34,18	33,31	31,92	34,66	33,42
hsa-miR-122-5p	YP00205664	35,00	35,00	35,00	35,00	35,00	35,00	35,00	35,00
hsa-miR-99a-3p	YP00204520	33,19	34,29	34,32	35,00	35,00	32,39	35,00	33,88
hsa-miR-361-5p	YP00206054	28,14	30,76	29,45	28,79	29,92	28,75	30,66	29,80
hsa-miR-202-3p	YP00205990	35,00	35,00	35,00	35,00	35,00	35,00	35,00	35,00
hsa-miR-125b-5p	YP00205713	21,04	24,32	22,69	24,41	23,21	21,75	23,78	24,24
hsa-miR-503-5p	YP00204334	33,70	34,37	35,00	34,08	30,85	27,73	30,84	28,49
hsa-miR-204-5p	YP00206072	34,07	35,00	33,97	35,00	35,00	35,00	35,00	35,00
hsa-miR-30d-5p	YP00206047	27,44	30,50	29,16	29,59	29,42	29,43	30,32	30,38
hsa-miR-301a-3p	YP00205601	30,87	33,81	32,45	35,00	35,00	34,03	35,00	33,65
hsa-miR-362-5p	YP00204618	34,57	35,00	34,50	35,00	35,00	33,64	35,00	33,33
hsa-miR-30b-3p	YP00206003	35,00	35,00	35,00	35,00	35,00	35,00	35,00	35,00
hsa-miR-654-5p	YP00204439	32,25	35,00	35,00	35,00	35,00	33,78	35,00	34,29
hsa-miR-545-3p	YP00206087	33,45	35,00	35,00	35,00	35,00	35,00	35,00	35,00
hsa-miR-29b-2-5p	YP00204208	34,35	35,00	34,26	35,00	35,00	35,00	35,00	35,00
hsa-miR-491-5p	YP00204695	30,39	33,39	33,11	35,00	33,02	32,75	32,41	32,66
hsa-miR-92b-3p	YP00204384	30,12	32,78	31,12	32,10	30,40	30,24	33,24	32,15
hsa-miR-665	YP00204710	32,70	34,48	35,00	33,03	31,94	31,97	33,15	32,27
hsa-miR-506-3p	YP00204539	35,00	35,00	35,00	35,00	35,00	35,00	35,00	35,00
hsa-miR-363-3p	YP00204726	35,00	35,00	35,00	35,00	35,00	35,00	35,00	35,00
hsa-miR-132-3p	YP00206035	27,57	31,79	31,05	30,72	31,19	29,42	30,87	31,05
hsa-miR-651-5p	YP00205864	35,00	35,00	35,00	35,00	35,00	35,00	35,00	35,00
hsa-miR-628-3p	YP00206057	33,40	35,00	35,00	35,00	35,00	35,00	35,00	35,00

hsa-miR-432-5p	YP00204776	30,30	31,87	31,53	30,77	32,22	33,03	33,38	35,00
hsa-miR-154-3p	YP00204096	32,70	35,00	35,00	35,00	35,00	32,61	35,00	35,00
hsa-miR-27a-3p	YP00206038	25,56	27,18	26,81	25,80	26,36	24,21	28,28	26,15
hsa-miR-376c-3p	YP00204442	27,06	29,10	28,67	28,95	29,19	28,11	30,80	29,11
hsa-miR-940	YP00204094	29,41	31,42	30,66	29,53	28,44	27,60	28,55	29,44
hsa-miR-22-5p	YP00204255	30,95	33,30	32,69	35,00	33,32	29,84	32,03	31,18
hsa-miR-224-5p	YP00204641	31,60	32,03	33,49	35,00	33,05	30,57	31,31	31,77
hsa-miR-885-5p	YP00204473	35,00	35,00	35,00	35,00	35,00	35,00	35,00	35,00
hsa-miR-320a	YP00206042	25,79	28,17	26,99	26,98	27,02	25,99	27,70	27,65
hsa-miR-18b-5p	YP00204084	30,89	35,00	32,31	35,00	33,33	31,80	33,42	32,80
hsa-miR-187-3p	YP00204018	35,00	35,00	35,00	35,00	35,00	35,00	35,00	35,00
hsa-miR-516b-5p	YP00204558	35,00	35,00	35,00	35,00	35,00	35,00	35,00	35,00
hsa-miR-302c-5p	YP00205940	35,00	35,00	35,00	35,00	35,00	35,00	35,00	35,00
hsa-miR-548b-3p	YP00204485	35,00	35,00	35,00	35,00	35,00	35,00	35,00	35,00
hsa-miR-186-5p	YP00206053	31,90	35,00	33,10	32,60	33,12	33,72	34,54	34,23
hsa-miR-199a-5p	YP00204494	25,39	29,23	26,66	27,11	27,26	25,80	27,85	26,29
hsa-miR-155-5p	YP00204308	29,57	32,05	31,09	35,00	32,96	31,88	31,90	31,71
hsa-miR-107	YP00204468	27,37	29,92	28,56	29,91	30,39	29,26	30,54	30,27
hsa-miR-302b-3p	YP00204773	35,00	35,00	35,00	35,00	35,00	35,00	35,00	35,00
hsa-miR-662	YP00204180	35,00	35,00	35,00	35,00	35,00	35,00	35,00	35,00
hsa-miR-519d-3p	YP00204062	35,00	35,00	35,00	35,00	35,00	35,00	35,00	35,00
hsa-miR-485-3p	YP00206055	30,80	32,84	33,29	34,65	33,02	31,18	33,44	32,63
hsa-miR-200b-3p	YP00206071	35,00	35,00	35,00	35,00	35,00	35,00	35,00	35,00
hsa-miR-337-3p	YP00205938	29,62	31,33	30,80	32,45	32,13	32,05	33,23	33,06
hsa-miR-494-3p	YP00204579	32,62	34,12	32,59	35,00	32,97	33,56	34,41	34,14
hsa-miR-371a-3p	YP00204299	35,00	35,00	35,00	35,00	35,00	35,00	35,00	35,00
hsa-miR-637	YP00205679	35,00	35,00	35,00	35,00	35,00	35,00	35,00	35,00
hsa-miR-144-3p	YP00204754	35,00	35,00	35,00	35,00	35,00	35,00	35,00	35,00
hsa-miR-16-1-3p	YP00206012	34,42	35,00	33,69	35,00	35,00	32,53	35,00	33,59
hsa-miR-631	YP00204312	35,00	35,00	35,00	35,00	35,00	35,00	35,00	35,00
hsa-miR-34c-5p	YP00205659	33,25	34,45	35,00	35,00	33,93	32,08	34,61	32,79
hsa-miR-211-5p	YP00204009	35,00	35,00	35,00	35,00	35,00	35,00	35,00	35,00
hsa-miR-454-3p	YP00205663	31,78	35,00	33,35	35,00	35,00	33,49	35,00	34,40
hsa-let-7f-5p	YP00204359	29,85	31,12	29,69	35,00	34,12	30,17	33,27	30,40
hsa-miR-30e-5p	YP00204714	29,35	31,60	29,89	35,00	33,11	30,20	33,04	30,90
hsa-miR-34a-5p	YP00204486	26,50	30,54	27,78	27,02	27,17	25,89	27,26	25,84
hsa-miR-663a	YP00204284	26,93	29,70	29,00	29,98	26,27	26,00	25,73	25,79
hsa-miR-518e-3p	YP00204051	35,00	35,00	35,00	35,00	35,00	35,00	35,00	35,00
hsa-miR-29b-3p	YP00204679	28,37	33,16	30,79	35,00	31,13	28,89	31,11	28,43
hsa-miR-658	YP00204522	35,00	35,00	35,00	35,00	35,00	35,00	35,00	35,00
hsa-miR-572	YP00204696	34,47	33,17	35,00	35,00	33,30	33,68	32,33	33,34
hsa-miR-802	YP00205980	35,00	35,00	35,00	35,00	35,00	35,00	35,00	35,00
hsa-miR-521	YP00205890	35,00	35,00	35,00	35,00	35,00	35,00	35,00	35,00
hsa-miR-433-3p	YP00204036	30,12	32,24	32,47	30,88	30,05	27,94	31,74	30,15
hsa-miR-660-5p	YP00205911	30,93	33,38	32,51	33,69	33,12	31,53	34,63	32,41
hsa-let-7c-5p	YP00204767	27,48	28,34	26,81	29,93	28,19	27,31	29,60	28,49

hsa-miR-28-5p	YP00204322	29,21	32,45	30,72	31,63	32,11	30,85	32,80	31,58
hsa-miR-324-5p	YP00204057	29,53	31,95	31,16	32,03	32,81	30,94	33,41	32,98
hsa-miR-219a-5p	YP00204780	33,27	35,00	34,50	35,00	35,00	35,00	35,00	35,00
hsa-miR-19b-3p	YP00204450	25,87	28,86	27,80	28,49	28,19	25,54	29,13	27,22
hsa-miR-526b-5p	YP00204259	35,00	35,00	35,00	35,00	35,00	35,00	35,00	35,00
hsa-miR-215-5p	YP00204598	35,00	35,00	35,00	35,00	35,00	35,00	35,00	34,65
hsa-miR-30b-5p	YP00204765	26,82	30,56	28,92	31,75	29,80	27,55	30,03	28,69
hsa-miR-184	YP00204601	35,00	35,00	35,00	35,00	35,00	35,00	35,00	35,00
hsa-miR-422a	YP00204164	35,00	35,00	35,00	35,00	35,00	35,00	35,00	35,00
hsa-miR-199a-3p	YP00204536	23,92	27,51	25,70	28,44	25,80	24,02	26,82	25,44
hsa-miR-335-5p	YP02119293	30,82	29,54	32,44	34,44	32,87	32,34	35,00	35,00
hsa-miR-519a-3p	YP00205919	35,00	35,00	35,00	35,00	35,00	35,00	35,00	35,00
hsa-miR-21-5p	YP00204230	22,20	24,61	23,78	23,68	24,13	22,23	25,29	23,04
hsa-miR-129-2-3p	YP00206067	34,41	35,00	35,00	35,00	35,00	34,09	35,00	35,00
hsa-miR-26b-5p	YP00204172	28,82	32,64	30,55	31,59	31,26	31,51	32,96	30,99
hsa-miR-214-3p	YP00204510	25,87	28,29	26,40	26,06	26,63	25,85	26,85	26,33
hsa-miR-32-5p	YP00204792	31,39	34,31	33,49	35,00	35,00	32,81	34,06	31,99
hsa-miR-324-3p	YP00204303	29,75	32,91	31,46	31,98	31,47	30,95	30,99	32,12
hsa-miR-488-3p	YP00204469	35,00	35,00	35,00	35,00	35,00	35,00	35,00	35,00
hsa-miR-371a-5p	YP00204493	35,00	35,00	35,00	35,00	35,00	35,00	35,00	35,00
hsa-miR-455-5p	YP00204363	32,20	33,10	34,48	32,06	35,00	30,93	33,65	31,35
hsa-miR-891a-5p	YP00204220	35,00	35,00	35,00	35,00	35,00	35,00	35,00	35,00
hsa-miR-549a	YP00205975	35,00	35,00	35,00	35,00	35,00	35,00	35,00	33,99
hsa-miR-205-5p	YP00204487	35,00	35,00	35,00	35,00	35,00	35,00	35,00	35,00
hsa-miR-518b	YP00204405	35,00	35,00	35,00	35,00	35,00	35,00	35,00	35,00
hsa-miR-19a-3p	YP00205862	25,82	28,99	27,70	27,66	28,02	26,95	29,32	26,68
hsa-miR-150-5p	YP00204660	35,00	35,00	35,00	35,00	35,00	35,00	35,00	34,44
hsa-miR-15a-5p	YP00204066	26,80	30,36	27,78	35,00	29,04	28,06	30,58	27,81
hsa-let-7d-3p	YP00205627	30,99	33,07	31,40	34,46	32,23	29,99	32,69	32,27
hsa-miR-608	YP00204286	35,00	35,00	35,00	35,00	35,00	35,00	35,00	35,00
hsa-miR-671-5p	YP00205649	31,80	33,68	32,78	35,00	35,00	32,12	34,66	33,42
hsa-miR-497-5p	YP00204354	32,73	35,00	33,93	35,00	32,19	31,66	33,83	32,28
hsa-miR-877-5p	YP00205626	34,37	35,00	32,95	35,00	33,73	33,57	35,00	35,00
hsa-miR-187-5p	YP00205920	35,00	35,00	35,00	35,00	35,00	35,00	35,00	35,00
hsa-miR-10b-5p	YP00205637	28,75	35,00	29,51	35,00	29,34	27,51	29,81	29,38
hsa-let-7i-5p	YP00204394	23,72	27,47	25,75	25,48	25,88	23,41	27,82	26,26
hsa-miR-202-5p	YP00205616	35,00	35,00	35,00	35,00	35,00	35,00	35,00	35,00
hsa-miR-652-3p	YP00204387	31,05	33,27	31,90	33,55	32,43	35,00	33,11	33,37
hsa-miR-126-5p	YP00206010	35,00	35,00	35,00	35,00	35,00	35,00	35,00	35,00
hsa-miR-30e-3p	YP00204410	30,66	31,76	32,73	35,00	35,00	31,73	32,40	32,48
hsa-miR-181c-5p	YP00204683	32,75	35,00	35,00	35,00	35,00	34,00	35,00	35,00
hsa-miR-9-3p	YP00204620	35,00	35,00	35,00	35,00	35,00	35,00	35,00	35,00
hsa-miR-548c-3p	YP00204697	35,00	35,00	35,00	35,00	35,00	35,00	35,00	35,00
hsa-miR-152-3p	YP00204294	26,62	30,07	29,06	29,07	29,21	28,99	30,73	29,52
hsa-miR-93-5p	YP00204715	28,08	31,11	29,12	29,72	30,74	28,46	30,73	29,60
hsa-miR-365a-3p	YP00204622	27,13	28,92	27,37	30,42	28,25	28,41	29,44	29,08

hsa-miR-29c-3p	YP00204729	25,37	29,77	27,05	28,75	26,65	26,68	28,20	26,20
hsa-miR-372-3p	YP00204137	35,00	35,00	35,00	35,00	35,00	35,00	35,00	35,00
hsa-miR-133a-3p	YP00204788	32,74	35,00	35,00	35,00	35,00	35,00	35,00	35,00
hsa-miR-124-3p	YP00206026	34,49	35,00	35,00	35,00	35,00	35,00	35,00	35,00
hsa-miR-190a-5p	YP00204763	33,80	35,00	34,24	35,00	34,45	33,22	35,00	34,47
hsa-miR-302a-3p	YP00206059	35,00	35,00	35,00	35,00	35,00	34,22	35,00	35,00
hsa-miR-595	YP00204070	35,00	35,00	35,00	35,00	35,00	35,00	35,00	35,00
hsa-miR-602	YP00204316	35,00	35,00	35,00	35,00	35,00	35,00	35,00	35,00
hsa-miR-223-3p	YP00205986	35,00	35,00	35,00	35,00	35,00	35,00	35,00	35,00
hsa-miR-627-5p	YP00205979	35,00	35,00	35,00	35,00	35,00	35,00	35,00	35,00
hsa-miR-34b-3p	YP00204005	35,00	35,00	35,00	35,00	35,00	33,93	35,00	35,00
hsa-miR-410-3p	YP00204042	30,67	32,75	31,95	33,02	33,34	31,46	35,00	34,31
hsa-miR-17-5p	YP02119304	28,31	30,69	29,80	30,95	30,13	28,76	30,95	29,32
hsa-miR-376a-3p	YP00204508	26,84	28,65	28,64	28,78	29,29	28,48	30,84	29,42
hsa-miR-514a-3p	YP00205931	35,00	35,00	35,00	35,00	35,00	35,00	35,00	35,00
hsa-miR-512-5p	YP00204134	35,00	35,00	35,00	35,00	35,00	35,00	35,00	35,00
hsa-miR-449a	YP00204481	35,00	35,00	35,00	35,00	35,00	35,00	35,00	35,00
hsa-miR-498	YP00204150	35,00	35,00	35,00	35,00	35,00	35,00	35,00	35,00
hsa-miR-148b-3p	YP00204047	28,54	32,73	30,46	33,66	31,33	30,04	33,86	31,35
hsa-miR-127-3p	YP00204048	26,94	29,08	28,56	29,50	29,69	29,05	29,73	29,27
hsa-miR-598-3p	YP00204320	32,58	35,00	35,00	35,00	35,00	35,00	35,00	35,00
hsa-miR-96-5p	YP00204417	35,00	35,00	35,00	35,00	35,00	35,00	35,00	35,00
hsa-let-7d-5p	YP00204124	28,56	30,23	29,26	31,42	30,39	28,97	31,80	30,15
hsa-miR-135b-5p	YP00204130	35,00	35,00	35,00	35,00	35,00	35,00	35,00	35,00
hsa-miR-495-3p	YP00206015	29,60	31,38	30,52	33,09	31,08	31,47	32,94	31,31
hsa-miR-299-5p	YP00204544	30,17	31,64	31,77	33,72	32,38	31,95	33,66	31,78
hsa-miR-34c-3p	YP00204373	35,00	35,00	33,91	32,17	33,94	31,75	35,00	31,87
hsa-miR-596	YP00204002	35,00	35,00	35,00	35,00	35,00	34,25	35,00	35,00
hsa-miR-744-5p	YP00204663	29,88	32,10	31,37	35,00	32,81	31,97	32,71	32,55
hsa-miR-145-5p	YP00204483	24,03	26,35	25,95	27,73	26,87	24,63	27,31	26,60
hsa-miR-622	YP00205615	35,00	33,06	35,00	35,00	35,00	35,00	35,00	35,00
hsa-miR-516a-5p	YP00204471	35,00	35,00	35,00	35,00	35,00	35,00	35,00	35,00
hsa-let-7a-5p	YP00205727	26,17	27,10	25,70	28,73	27,84	25,56	28,29	27,28
hsa-miR-96-3p	YP00205899	35,00	35,00	35,00	35,00	35,00	35,00	35,00	35,00
hsa-miR-185-3p	YP00205710	35,00	34,23	35,00	34,58	35,00	35,00	35,00	35,00
hsa-miR-615-3p	YP00204453	33,44	34,31	34,57	32,81	30,90	30,61	31,86	31,43
hsa-miR-128-3p	YP00205995	31,21	34,44	35,00	35,00	33,16	32,01	35,00	33,98
hsa-miR-766-3p	YP00204499	33,57	35,00	34,02	35,00	35,00	32,14	35,00	35,00
hsa-miR-206	YP00206073	35,00	35,00	35,00	35,00	35,00	35,00	35,00	35,00
hsa-miR-298	YP00204115	35,00	35,00	35,00	35,00	35,00	35,00	35,00	35,00
hsa-miR-193a-5p	YP00204665	27,77	29,08	28,44	30,67	28,84	27,44	29,36	29,57
hsa-miR-449b-5p	YP00204751	35,00	35,00	35,00	35,00	35,00	35,00	35,00	35,00
hsa-miR-520d-5p	YP00204684	35,00	35,00	35,00	35,00	35,00	35,00	35,00	35,00
hsa-miR-192-5p	YP00204099	32,79	35,00	33,73	34,01	35,00	35,00	35,00	35,00
hsa-miR-29a-3p	YP00204698	24,51	28,90	26,70	27,78	26,11	25,47	27,10	25,69
hsa-miR-18a-3p	YP00204523	32,21	35,00	33,83	35,00	35,00	33,78	35,00	34,37

hsa-miR-383-5p	YP00205904	35,00	35,00	35,00	35,00	35,00	35,00	35,00	35,00
hsa-miR-9-5p	YP00204513	35,00	35,00	35,00	35,00	35,00	35,00	35,00	35,00
hsa-miR-142-5p	YP00204722	35,00	35,00	35,00	35,00	35,00	35,00	35,00	34,57
hsa-miR-363-5p	YP00204173	35,00	35,00	35,00	35,00	35,00	35,00	35,00	35,00
hsa-miR-147b	YP00204368	35,00	35,00	35,00	35,00	35,00	35,00	35,00	35,00
hsa-miR-197-3p	YP00204380	29,77	32,01	31,26	30,39	30,75	30,17	31,39	31,08
hsa-miR-597-5p	YP00204769	35,00	35,00	35,00	35,00	35,00	35,00	35,00	35,00
hsa-miR-326	YP00204512	32,32	33,73	33,56	32,12	32,06	31,01	33,67	31,88
hsa-miR-15b-5p	YP00204243	26,92	30,30	28,52	29,79	30,05	27,70	31,13	28,47
hsa-miR-105-5p	YP00204389	35,00	35,00	35,00	35,00	35,00	35,00	35,00	35,00
hsa-miR-196b-5p	YP00204555	35,00	35,00	35,00	35,00	33,24	30,72	35,00	31,75
hsa-miR-296-5p	YP00204436	31,66	33,67	32,74	34,63	33,39	31,54	33,13	32,22
hsa-miR-20b-5p	YP00204755	35,00	35,00	35,00	35,00	35,00	35,00	35,00	35,00
hsa-miR-147a	YP00204398	35,00	35,00	35,00	35,00	35,00	35,00	35,00	35,00
hsa-miR-198	YP00204341	31,63	31,10	35,00	34,41	30,45	29,80	32,32	29,87
hsa-miR-375	YP00204362	35,00	35,00	35,00	35,00	35,00	35,00	35,00	35,00
hsa-miR-517a-3p	YP00206019	35,00	35,00	35,00	35,00	35,00	35,00	35,00	35,00
hsa-miR-361-3p	YP00204008	32,46	35,00	35,00	35,00	34,69	33,25	35,00	35,00
hsa-miR-21-3p	YP00204302	28,78	30,68	30,71	28,82	29,05	27,02	29,93	27,36
hsa-miR-373-3p	YP00204604	35,00	35,00	35,00	35,00	35,00	35,00	35,00	35,00
hsa-miR-518f-3p	YP00204140	35,00	35,00	35,00	35,00	35,00	35,00	35,00	35,00
hsa-miR-222-3p	YP00204551	24,61	27,07	25,51	30,05	25,41	24,99	26,76	27,11
hsa-miR-617	YP00204564	35,00	35,00	35,00	35,00	35,00	35,00	35,00	35,00
hsa-miR-154-5p	YP00204518	29,24	31,96	31,30	31,14	31,75	30,31	32,73	32,43
hsa-miR-708-5p	YP00204490	27,97	31,52	29,18	30,49	30,72	28,56	30,82	29,36
hsa-let-7b-5p	YP00204750	27,13	28,32	26,43	28,10	26,85	25,20	27,28	26,38
hsa-miR-95-3p	YP00204288	35,00	35,00	35,00	35,00	35,00	35,00	35,00	35,00
hsa-miR-517c-3p	YP00204177	35,00	35,00	35,00	32,49	35,00	35,00	35,00	35,00
hsa-miR-151a-5p	YP00204007	26,61	29,64	28,53	29,09	29,90	28,25	30,15	29,88
hsa-miR-502-5p	YP00204449	34,42	35,00	35,00	32,42	33,73	35,00	35,00	35,00
hsa-miR-345-5p	YP00206006	30,69	31,60	32,40	30,97	32,97	31,91	31,34	30,39
hsa-miR-509-3p	YP00204458	35,00	35,00	35,00	35,00	35,00	35,00	35,00	35,00
hsa-miR-134-5p	YP00205989	29,85	31,14	31,07	31,52	31,31	29,90	32,12	31,91
hsa-miR-382-5p	YP00204169	28,29	31,07	31,71	32,17	32,26	29,42	32,66	32,43
hsa-miR-490-3p	YP00205999	35,00	35,00	35,00	35,00	35,00	35,00	35,00	35,00
hsa-miR-200c-3p	YP00204482	35,00	35,00	35,00	35,00	35,00	35,00	35,00	35,00
hsa-miR-30a-5p	YP00205695	28,55	29,60	29,68	31,64	31,43	29,34	32,65	30,78
hsa-miR-181b-5p	YP00204530	28,88	29,75	29,74	29,54	31,19	28,95	31,46	30,85
hsa-miR-33a-5p	YP00205690	29,70	33,31	30,61	34,28	32,00	30,00	34,14	29,91
hsa-miR-195-5p	YP00205869	35,00	35,00	35,00	35,00	34,07	32,98	35,00	33,59
hsa-miR-874-3p	YP00204761	29,93	31,21	31,75	30,59	30,07	27,95	29,27	28,30
hsa-miR-135a-5p	YP00204762	35,00	35,00	35,00	35,00	35,00	35,00	35,00	35,00
hsa-miR-26a-2-3p	YP00204676	32,87	35,00	33,98	35,00	35,00	33,80	35,00	35,00
hsa-miR-146b-5p	YP00204553	31,42	35,00	32,52	35,00	32,79	32,29	33,76	32,84
hsa-miR-412-3p	YP00204460	34,64	35,00	35,00	35,00	35,00	35,00	35,00	35,00
hsa-miR-1-3p	YP00204344	35,00	35,00	35,00	35,00	35,00	35,00	35,00	35,00

hsa-miR-299-3p	YP00204702	33,53	35,00	35,00	35,00	35,00	34,06	34,37	35,00
hsa-miR-142-3p	YP00204291	35,00	35,00	35,00	35,00	35,00	34,55	35,00	34,16
hsa-miR-338-3p	YP00204719	35,00	35,00	35,00	35,00	35,00	35,00	35,00	35,00
hsa-miR-584-5p	YP00204568	35,00	35,00	26,72	35,00	35,00	35,00	35,00	35,00
hsa-miR-377-3p	YP00204733	30,81	33,16	31,51	33,88	31,39	30,94	32,30	30,97
hsa-miR-216a-5p	YP00204167	35,00	35,00	35,00	35,00	35,00	34,48	35,00	35,00
hsa-miR-424-5p	YP00204736	29,18	30,80	29,74	32,18	27,91	24,88	27,25	24,71
hsa-miR-921	YP00204281	35,00	35,00	35,00	35,00	35,00	35,00	35,00	35,00
hsa-miR-513a-5p	YP00205900	35,00	35,00	35,00	35,00	35,00	35,00	35,00	35,00
hsa-miR-140-3p	YP00204304	29,21	29,85	29,93	30,89	30,22	30,08	32,49	31,18
hsa-miR-181a-5p	YP00206081	26,40	27,78	27,48	29,66	29,43	26,99	28,35	26,99
hsa-miR-10a-5p	YP00204778	35,00	35,00	35,00	35,00	33,18	28,78	31,12	30,39
hsa-miR-106a-5p	YP00204563	27,83	31,29	29,86	29,66	30,47	28,78	31,49	28,76
hsa-miR-182-5p	YP00206070	35,00	35,00	35,00	35,00	35,00	35,00	35,00	35,00
hsa-miR-370-3p	YP00204011	33,54	35,00	33,99	33,02	33,44	35,00	35,00	31,43
hsa-miR-576-5p	YP00206064	35,00	35,00	35,00	32,97	35,00	35,00	35,00	35,00
hsa-miR-425-3p	YP00204038	31,61	35,00	33,31	33,62	34,07	31,67	34,26	34,20
hsa-miR-450a-5p	YP00206085	33,07	34,05	33,82	34,05	32,13	30,54	33,23	32,10
hsa-miR-411-5p	YP00204531	28,85	31,52	31,72	32,62	33,77	31,96	34,66	33,74
hsa-miR-216b-5p	YP00204289	35,00	35,00	35,00	35,00	35,00	35,00	35,00	35,00
hsa-miR-106b-5p	YP00205884	27,92	30,64	29,05	29,61	30,89	28,77	31,77	28,85
hsa-miR-22-3p	YP00204606	24,88	27,79	26,49	28,82	26,26	25,07	27,44	26,37
hsa-miR-510-5p	YP00204349	35,00	35,00	35,00	35,00	35,00	35,00	35,00	35,00
hsa-miR-212-3p	YP00204170	31,76	35,00	34,63	35,00	35,00	34,43	35,00	35,00
hsa-miR-525-5p	YP00204355	35,00	35,00	35,00	35,00	35,00	35,00	35,00	35,00
hsa-miR-542-5p	YP00204198	33,44	35,00	34,50	35,00	33,39	30,71	33,11	32,82
hsa-miR-576-3p	YP00204195	35,00	35,00	35,00	35,00	35,00	35,00	35,00	35,00
hsa-miR-583	YP00204021	35,00	35,00	35,00	35,00	35,00	35,00	35,00	35,00
hsa-miR-483-3p	YP00204012	35,00	34,65	35,00	33,06	35,00	32,99	33,75	35,00
hsa-miR-582-5p	YP00204254	35,00	35,00	35,00	33,95	35,00	35,00	35,00	35,00
hsa-miR-183-5p	YP00206030	35,00	35,00	35,00	35,00	35,00	35,00	35,00	35,00
hsa-miR-33b-5p	YP00205860	35,00	35,00	35,00	35,00	35,00	33,91	35,00	35,00
hsa-miR-193a-3p	YP00204591	33,17	35,00	33,40	35,00	34,31	35,00	35,00	33,37
hsa-miR-153-3p	YP00204338	35,00	35,00	35,00	35,00	35,00	35,00	35,00	35,00
hsa-let-7e-5p	YP00205711	29,16	29,66	28,90	28,07	29,87	28,10	30,52	30,06
hsa-miR-409-3p	YP00204358	29,64	31,65	31,66	31,60	31,67	30,75	32,64	32,03
hsa-miR-100-5p	YP00205689	25,07	25,83	25,36	26,09	26,72	24,97	27,04	27,28
hsa-miR-629-5p	YP00204370	33,81	35,00	35,00	35,00	35,00	35,00	35,00	35,00
hsa-miR-484	YP00205636	28,40	31,01	29,57	31,66	30,03	28,27	30,23	30,60
hsa-miR-429	YP00205901	35,00	35,00	35,00	35,00	35,00	35,00	35,00	35,00
hsa-miR-30c-2-3p	YP00205632	35,00	35,00	35,00	35,00	35,00	35,00	35,00	35,00
hsa-miR-518a-3p	YP00206069	35,00	35,00	35,00	35,00	35,00	35,00	35,00	35,00
hsa-miR-340-5p	YP00206068	33,58	34,40	34,53	35,00	35,00	35,00	35,00	35,00
hsa-miR-508-3p	YP00204480	35,00	35,00	35,00	35,00	35,00	35,00	35,00	35,00
hsa-miR-381-3p	YP00205887	31,12	33,38	31,84	32,10	33,19	31,44	33,09	32,31
hsa-miR-148a-3p	YP00205867	35,00	35,00	35,00	35,00	30,32	28,81	31,14	29,56

hsa-miR-146a-5p	YP00204688	32,33	32,39	35,00	35,00	35,00	32,41	34,62	31,85
hsa-miR-139-5p	YP00205874	35,00	35,00	35,00	35,00	35,00	35,00	35,00	35,00
hsa-miR-373-5p	YP00204703	35,00	35,00	35,00	35,00	35,00	33,61	35,00	33,68
hsa-miR-149-5p	YP00204321	31,91	32,86	33,32	32,90	35,00	33,51	35,00	35,00
hsa-miR-642a	YP00204793	35,00	35,00	35,00	35,00	35,00	35,00	35,00	35,00
hsa-miR-31-5p	YP00204236	24,41	27,19	25,80	26,14	26,76	26,05	27,84	26,12
hsa-miR-451a	YP02119305	35,00	35,00	31,60	35,00	35,00	35,00	35,00	35,00
hsa-miR-620	YP00204637	35,00	35,00	35,00	35,00	35,00	35,00	35,00	35,00
hsa-miR-27b-3p	YP00205915	26,81	27,27	26,97	28,69	27,31	25,94	28,60	27,04
hsa-miR-523-3p	YP00204452	35,00	35,00	35,00	35,00	35,00	35,00	35,00	35,00
hsa-miR-374a-5p	YP00204758	29,02	31,39	30,12	31,43	31,33	30,93	33,62	30,30
hsa-miR-92a-1-5p	YP00204560	34,88	35,00	35,00	35,00	35,00	35,00	35,00	35,00
hsa-miR-219a-1-3p	YP00205880	35,00	35,00	35,00	35,00	35,00	35,00	35,00	35,00
hsa-miR-1913	YP00204673	34,66	34,32	32,66	35,00	33,92	32,76	33,72	30,90
hsa-miR-1245a	YP00204229	35,00	35,00	35,00	35,00	35,00	35,00	35,00	35,00
hsa-miR-522-3p	YP00205913	35,00	35,00	35,00	35,00	35,00	35,00	35,00	35,00
hsa-miR-571	YP00206065	35,00	35,00	35,00	35,00	35,00	35,00	35,00	35,00
hsa-miR-323a-5p	YP00206080	35,00	35,00	35,00	35,00	35,00	35,00	35,00	35,00
hsa-miR-592	YP00204375	35,00	35,00	35,00	35,00	35,00	35,00	35,00	35,00
hsa-miR-487a-3p	YP00204381	33,71	34,73	34,08	34,19	35,00	35,00	34,11	35,00
hsa-miR-1249	YP00204122	35,00	35,00	34,04	35,00	35,00	35,00	34,95	35,00
hsa-miR-25-5p	YP00204031	35,00	35,00	35,00	35,00	35,00	35,00	35,00	35,00
hsa-miR-922	YP00204478	35,00	35,00	35,00	35,00	35,00	35,00	35,00	35,00
hsa-miR-124-5p	YP00204266	35,00	35,00	35,00	35,00	35,00	35,00	35,00	35,00
hsa-miR-1264	YP00204056	35,00	35,00	35,00	35,00	35,00	35,00	35,00	35,00
hsa-miR-504-5p	YP00204396	35,00	35,00	35,00	35,00	35,00	35,00	35,00	35,00
hsa-miR-138-1-3p	YP00205881	35,00	35,00	35,00	33,27	34,02	32,58	35,00	35,00
hsa-miR-502-3p	YP00204043	34,16	35,00	32,43	35,00	33,79	33,75	35,00	33,34
hsa-miR-490-5p	YP00206077	35,00	35,00	35,00	35,00	35,00	35,00	35,00	35,00
hsa-miR-567	YP00204165	27,56	33,34	31,97	35,00	30,96	31,98	29,08	35,00
hsa-miR-18b-3p	YP00204089	35,00	35,00	35,00	35,00	35,00	35,00	35,00	35,00
hsa-miR-125a-3p	YP00204446	33,09	35,00	32,97	33,34	33,80	32,40	35,00	34,73
hsa-miR-653-5p	YP00204669	35,00	35,00	35,00	35,00	35,00	35,00	35,00	35,00
hsa-miR-891b	YP00204109	35,00	35,00	35,00	35,00	35,00	35,00	35,00	35,00
hsa-miR-144-5p	YP00204670	35,00	35,00	35,00	35,00	35,00	35,00	35,00	35,00
hsa-miR-1538	YP00204615	34,53	35,00	35,00	34,59	33,88	33,04	35,00	32,98
hsa-miR-384	YP00205879	35,00	35,00	35,00	35,00	35,00	35,00	35,00	35,00
hsa-miR-196b-3p	YP00206018	35,00	35,00	35,00	35,00	35,00	34,36	35,00	34,40
hsa-miR-649	YP00204146	35,00	35,00	35,00	35,00	35,00	35,00	35,00	35,00
hsa-miR-143-5p	YP00204570	31,03	32,71	31,24	35,00	34,07	31,35	33,37	32,31
hsa-miR-1207-5p	YP00204693	32,90	34,26	33,14	34,03	33,22	32,78	33,96	32,03
hsa-miR-943	YP00204545	35,00	35,00	35,00	35,00	35,00	35,00	35,00	35,00
hsa-miR-675-3p	YP00204732	35,00	35,00	35,00	35,00	32,73	35,00	35,00	35,00
hsa-miR-200b-5p	YP00204144	35,00	35,00	35,00	35,00	33,66	34,43	35,00	33,88
hsa-miR-519e-5p	YP00204029	35,00	35,00	35,00	35,00	35,00	35,00	35,00	35,00
hsa-miR-942-5p	YP00204440	35,00	35,00	35,00	35,00	35,00	35,00	35,00	35,00

hsa-miR-450b-3p	YP00205683	35,00	35,00	35,00	35,00	35,00	35,00	35,00	35,00
hsa-miR-553	YP00204612	35,00	35,00	35,00	35,00	35,00	35,00	35,00	35,00
hsa-miR-605-5p	YP00205594	35,00	35,00	35,00	35,00	35,00	35,00	35,00	35,00
hsa-miR-24-2-5p	YP00204187	30,91	34,05	32,21	35,00	34,75	33,16	34,87	33,08
hsa-miR-23a-5p	YP00205631	35,00	35,00	35,00	35,00	35,00	33,93	33,75	33,80
hsa-miR-27b-5p	YP00204219	35,00	35,00	35,00	35,00	35,00	35,00	35,00	35,00
hsa-miR-759	YP00206000	31,59	35,00	35,00	34,48	35,00	35,00	35,00	35,00
hsa-miR-770-5p	YP00204197	35,00	35,00	34,80	35,00	35,00	35,00	35,00	35,00
hsa-miR-585-3p	YP00205701	35,00	35,00	25,89	33,62	35,00	35,00	35,00	31,46
hsa-miR-376a-5p	YP00205988	30,05	32,55	31,00	32,33	34,04	32,51	35,00	34,80
hsa-miR-507	YP00205662	35,00	35,00	35,00	35,00	35,00	35,00	35,00	35,00
hsa-miR-520b	YP00205939	35,00	35,00	35,00	35,00	35,00	35,00	35,00	35,00
hsa-miR-302f	YP00205634	35,00	35,00	35,00	35,00	35,00	35,00	35,00	35,00
hsa-miR-28-3p	YP00204119	30,86	31,86	31,62	34,02	32,40	31,89	33,15	32,84
hsa-miR-875-5p	YP00205978	35,00	35,00	35,00	35,00	35,00	35,00	35,00	35,00
hsa-miR-219a-2-3p	YP00204674	35,00	35,00	35,00	35,00	35,00	35,00	35,00	35,00
hsa-miR-1183	YP00204176	35,00	35,00	35,00	35,00	35,00	35,00	35,00	35,00
hsa-miR-758-3p	YP00204353	35,00	35,00	35,00	35,00	35,00	35,00	35,00	35,00
hsa-miR-1244	YP00204245	35,00	35,00	35,00	35,00	35,00	35,00	35,00	35,00
hsa-miR-566	YP00204282	34,27	35,00	34,47	35,00	35,00	34,17	35,00	34,08
hsa-miR-1256	YP00204059	33,35	35,00	32,71	35,00	35,00	35,00	35,00	35,00
hsa-miR-516a-3p	YP00204305	35,00	35,00	35,00	35,00	35,00	35,00	35,00	35,00
hsa-miR-548c-5p	YP00205882	35,00	35,00	34,29	35,00	35,00	34,78	35,00	33,85
hsa-miR-496	YP00204441	33,68	35,00	34,83	33,62	35,00	35,00	35,00	35,00
hsa-miR-876-3p	YP00205927	35,00	35,00	35,00	35,00	35,00	35,00	35,00	35,00
hsa-miR-532-3p	YP00204003	31,74	33,42	31,73	34,10	32,83	32,32	33,14	34,01
hsa-miR-654-3p	YP00204054	31,76	32,60	31,53	35,00	32,76	32,12	29,57	33,04
hsa-miR-659-3p	YP00204199	35,00	35,00	35,00	35,00	35,00	35,00	35,00	35,00
hsa-miR-135b-3p	YP00204427	35,00	35,00	35,00	35,00	35,00	35,00	35,00	35,00
hsa-miR-641	YP00204411	35,00	35,00	35,00	35,00	35,00	35,00	35,00	35,00
hsa-miR-2113	YP00204686	35,00	35,00	35,00	35,00	35,00	35,00	35,00	34,67
hsa-miR-1254	YP00204685	33,54	33,81	34,23	35,00	34,08	33,23	34,82	33,44
hsa-miR-661	YP00204657	34,90	35,00	33,98	35,00	33,73	34,17	35,00	33,19
hsa-miR-892a	YP00204092	35,00	35,00	35,00	35,00	35,00	35,00	35,00	35,00
hsa-miR-10b-3p	YP00205909	35,00	35,00	35,00	35,00	35,00	35,00	35,00	35,00
hsa-miR-122-3p	YP00204437	35,00	35,00	35,00	35,00	35,00	35,00	35,00	35,00
hsa-miR-100-3p	YP00204535	34,65	34,85	34,75	35,00	35,00	35,00	35,00	35,00
hsa-miR-769-3p	YP00204104	35,00	35,00	35,00	35,00	35,00	35,00	35,00	35,00
hsa-miR-300	YP00204756	35,00	35,00	35,00	35,00	35,00	35,00	35,00	34,31
hsa-miR-518e-5p	YP00206040	35,00	35,00	35,00	35,00	35,00	35,00	35,00	35,00
hsa-miR-489-3p	YP00204395	35,00	35,00	35,00	35,00	35,00	35,00	35,00	35,00
hsa-miR-937-3p	YP00204060	35,00	35,00	35,00	35,00	35,00	35,00	35,00	35,00
hsa-miR-381-5p	YP00206075	35,00	35,00	35,00	34,28	35,00	35,00	35,00	35,00
hsa-miR-640	YP00204533	35,00	35,00	35,00	35,00	35,00	35,00	35,00	34,85
hsa-miR-148b-5p	YP00204735	35,00	35,00	35,00	35,00	35,00	35,00	35,00	35,00
hsa-miR-29c-5p	YP00204132	35,00	35,00	35,00	35,00	35,00	35,00	35,00	34,83

hsa-miR-499a-3p	YP00204438	35,00	35,00	35,00	35,00	35,00	35,00	35,00	35,00
hsa-let-7f-1-3p	YP00204323	31,17	33,23	32,01	34,82	32,93	31,15	34,14	32,94
hsa-miR-382-3p	YP00206066	32,71	34,12	35,00	34,46	35,00	34,24	35,00	33,72
hsa-miR-609	YP00204613	35,00	35,00	35,00	35,00	35,00	35,00	35,00	35,00
hsa-miR-10a-3p	YP00205688	35,00	35,00	35,00	35,00	35,00	35,00	35,00	35,00
hsa-miR-106a-3p	YP00204443	35,00	35,00	35,00	35,00	35,00	35,00	35,00	35,00
hsa-let-7e-3p	YP00205301	32,99	34,19	33,76	35,00	35,00	33,72	34,25	35,00
hsa-miR-580-3p	YP00204505	35,00	35,00	35,00	35,00	35,00	35,00	35,00	35,00
hsa-miR-761	YP00205475	35,00	35,00	35,00	35,00	35,00	35,00	35,00	35,00
hsa-miR-643	YP00204275	35,00	35,00	34,32	35,00	35,00	35,00	35,00	35,00
hsa-miR-618	YP00205674	34,60	35,00	35,00	35,00	35,00	35,00	35,00	35,00
hsa-miR-221-5p	YP00204032	32,71	34,60	31,66	35,00	32,04	31,17	33,89	31,86
hsa-miR-513b-5p	YP00205620	35,00	35,00	35,00	35,00	35,00	35,00	35,00	35,00
hsa-miR-411-3p	YP00204434	35,00	35,00	33,86	35,00	35,00	35,00	35,00	35,00
hsa-miR-19a-5p	YP00205604	35,00	35,00	35,00	35,00	35,00	35,00	35,00	35,00
hsa-miR-338-5p	YP00204114	35,00	35,00	35,00	35,00	35,00	35,00	35,00	35,00
hsa-miR-1914-3p	YP00204644	35,00	35,00	35,00	35,00	35,00	35,00	35,00	35,00
hsa-miR-323b-5p	YP00204796	35,00	35,00	35,00	35,00	35,00	35,00	35,00	35,00
hsa-miR-548i	YP00204103	35,00	35,00	35,00	35,00	35,00	35,00	35,00	35,00
hsa-miR-541-3p	YP00204276	35,00	35,00	35,00	35,00	35,00	35,00	35,00	35,00
hsa-miR-1272	YP00205863	35,00	35,00	35,00	33,17	35,00	35,00	35,00	35,00
hsa-miR-1205	YP00204143	35,00	35,00	35,00	35,00	35,00	35,00	35,00	35,00
hsa-miR-544a	YP00204646	35,00	35,00	35,00	35,00	35,00	35,00	35,00	35,00
hsa-miR-431-3p	YP00204567	33,97	34,30	31,76	33,06	34,65	34,85	35,00	34,45
hsa-miR-621	YP00204307	35,00	35,00	35,00	35,00	35,00	35,00	35,00	35,00
hsa-miR-556-5p	YP00204240	35,00	35,00	35,00	35,00	35,00	35,00	35,00	35,00
hsa-miR-1267	YP00204543	35,00	35,00	35,00	35,00	35,00	35,00	35,00	35,00
hsa-miR-141-5p	YP00206088	35,00	35,00	35,00	35,00	35,00	35,00	35,00	35,00
hsa-miR-1269a	YP00204455	35,00	35,00	35,00	35,00	35,00	35,00	35,00	35,00
hsa-miR-501-3p	YP00204178	32,96	35,00	33,18	35,00	34,87	33,43	33,93	33,78
hsa-miR-15b-3p	YP00205898	33,23	35,00	34,95	35,00	35,00	34,26	35,00	34,83
hsa-miR-146b-3p	YP00204374	33,73	35,00	35,00	35,00	35,00	35,00	35,00	35,00
hsa-miR-222-5p	YP00204314	33,38	33,67	31,93	35,00	30,56	29,74	31,64	29,79
hsa-miR-601	YP00204426	35,00	35,00	35,00	35,00	35,00	35,00	35,00	35,00
hsa-miR-924	YP02119297	35,00	35,00	35,00	35,00	35,00	35,00	35,00	35,00
hsa-miR-29a-5p	YP00204430	29,46	32,18	30,82	32,18	30,93	30,43	32,38	31,01
hsa-let-7a-2-3p	YP00206045	34,04	33,27	32,87	35,00	35,00	32,80	34,54	34,93
hsa-miR-520f-3p	YP00204283	35,00	35,00	35,00	35,00	35,00	35,00	35,00	35,00
hsa-miR-101-5p	YP00204379	35,00	35,00	35,00	35,00	35,00	35,00	35,00	35,00
hsa-miR-520a-3p	YP00204074	35,00	35,00	35,00	35,00	35,00	35,00	35,00	35,00
hsa-miR-548m	YP00205929	35,00	35,00	35,00	35,00	35,00	35,00	35,00	35,00
hsa-miR-517-5p	YP00204336	35,00	35,00	35,00	35,00	35,00	35,00	35,00	35,00
hsa-miR-448	YP00204643	35,00	35,00	35,00	35,00	35,00	35,00	35,00	35,00
hsa-miR-1296-5p	YP00206020	32,00	34,07	32,84	35,00	33,13	32,37	33,30	33,26
hsa-miR-1537-3p	YP00205994	35,00	35,00	35,00	35,00	35,00	35,00	35,00	35,00
hsa-miR-920	YP00204248	35,00	35,00	35,00	35,00	35,00	35,00	35,00	35,00

hsa-miR-1247-5p	YP00204770	35,00	35,00	35,00	35,00	35,00	35,00	35,00	35,00
hsa-miR-19b-2-5p	YP00205897	35,00	35,00	35,00	35,00	35,00	35,00	35,00	35,00
hsa-miR-558	YP00205670	35,00	35,00	35,00	35,00	35,00	35,00	35,00	35,00
hsa-miR-106b-3p	YP00204020	34,02	34,97	33,34	35,00	35,00	33,62	35,00	35,00
hsa-miR-1258	YP00205709	35,00	35,00	35,00	35,00	35,00	35,00	35,00	35,00
hsa-miR-619-3p	YP00204120	35,00	35,00	35,00	35,00	35,00	35,00	35,00	35,00
hsa-miR-208a-3p	YP00205619	35,00	35,00	33,46	35,00	35,00	35,00	35,00	35,00
hsa-miR-17-3p	YP00206008	33,17	35,00	32,46	35,00	35,00	34,24	35,00	34,12
hsa-miR-136-3p	YP00205503	29,78	33,07	31,00	31,30	32,27	31,45	34,53	32,16
hsa-miR-877-3p	YP00204263	35,00	35,00	35,00	35,00	35,00	35,00	35,00	35,00
hsa-miR-935	YP00205579	35,00	35,00	35,00	35,00	35,00	35,00	35,00	35,00
hsa-miR-224-3p	YP00204629	31,31	32,34	34,32	32,11	32,99	32,09	32,94	32,57
hsa-miR-624-3p	YP00205684	35,00	35,00	35,00	35,00	35,00	35,00	35,00	35,00
hsa-miR-767-5p	YP00204238	35,00	35,00	35,00	35,00	35,00	35,00	35,00	35,00
hsa-miR-559	YP00205883	35,00	35,00	35,00	35,00	35,00	35,00	35,00	35,00
hsa-miR-449b-3p	YP00204631	35,00	35,00	35,00	35,00	35,00	35,00	35,00	35,00
hsa-miR-205-3p	YP00205602	35,00	35,00	35,00	35,00	35,00	35,00	35,00	35,00
hsa-miR-604	YP00204075	35,00	35,00	35,00	35,00	35,00	35,00	35,00	35,00
hsa-miR-130b-5p	YP00204456	35,00	35,00	34,34	35,00	35,00	35,00	35,00	35,00
hsa-miR-149-3p	YP00204093	35,00	35,00	35,00	35,00	35,00	35,00	35,00	35,00
hsa-miR-1271-5p	YP00204351	33,22	35,00	32,94	35,00	35,00	34,49	34,64	33,73
hsa-miR-520h	YP00206050	35,00	35,00	35,00	35,00	35,00	35,00	35,00	35,00
hsa-miR-769-5p	YP00204270	32,78	34,17	32,95	35,00	35,00	33,05	35,00	34,82
hsa-miR-612	YP00204068	34,65	35,00	35,00	35,00	33,66	32,13	35,00	33,48
hsa-miR-1237-3p	YP00204069	35,00	35,00	35,00	35,00	35,00	35,00	35,00	34,48
hsa-miR-1908-5p	YP00204587	35,00	35,00	34,73	35,00	35,00	35,00	35,00	35,00
hsa-miR-1260a	YP00205892	20,56	20,40	19,28	20,94	18,63	17,84	19,38	19,20
hsa-miR-182-3p	YP00204098	35,00	35,00	35,00	35,00	35,00	35,00	35,00	35,00
hsa-miR-365b-5p	YP00204654	35,00	35,00	35,00	35,00	35,00	35,00	35,00	35,00
hsa-miR-508-5p	YP00204077	35,00	35,00	35,00	35,00	35,00	35,00	35,00	35,00
hsa-miR-671-3p	YP00204024	33,88	35,00	35,00	35,00	33,80	33,73	35,00	35,00
hsa-miR-941	YP00204574	34,94	35,00	35,00	35,00	35,00	34,39	35,00	35,00
hsa-miR-23b-5p	YP00205873	35,00	35,00	35,00	35,00	34,33	35,00	35,00	35,00
hsa-miR-591	YP00205872	35,00	35,00	35,00	35,00	35,00	35,00	35,00	35,00
hsa-miR-26b-3p	YP00204117	35,00	35,00	35,00	35,00	35,00	35,00	35,00	35,00
hsa-miR-519b-3p	YP00204332	35,00	35,00	35,00	35,00	35,00	35,00	35,00	35,00
hsa-miR-30d-3p	YP00204023	32,78	32,47	34,08	35,00	35,00	32,97	35,00	35,00
hsa-miR-518d-5p	YP00204461	35,00	35,00	35,00	35,00	35,00	35,00	35,00	35,00
hsa-miR-212-5p	YP00205401	34,23	35,00	34,76	35,00	35,00	35,00	35,00	35,00
hsa-miR-520e	YP00204013	35,00	35,00	35,00	35,00	35,00	35,00	35,00	35,00
hsa-miR-646	YP00204546	35,00	35,00	35,00	35,00	35,00	35,00	35,00	35,00
hsa-miR-519e-3p	YP00204491	35,00	35,00	35,00	35,00	35,00	35,00	35,00	35,00
hsa-miR-626	YP00204712	35,00	35,00	35,00	35,00	35,00	35,00	35,00	35,00
hsa-miR-26a-1-3p	YP00204184	35,00	35,00	35,00	35,00	35,00	35,00	35,00	35,00
hsa-miR-190b	YP00206031	35,00	35,00	35,00	35,00	35,00	35,00	35,00	35,00
hsa-miR-1471	YP00204623	35,00	35,00	35,00	35,00	35,00	35,00	35,00	35,00

hsa-miR-548l	YP00205891	35,00	35,00	35,00	35,00	35,00	35,00	35,00	35,00
hsa-miR-586	YP00204205	35,00	35,00	35,00	35,00	35,00	35,00	35,00	35,00
hsa-miR-103b	YP00204740	35,00	35,00	35,00	35,00	35,00	35,00	35,00	35,00
hsa-miR-488-5p	YP00205981	35,00	35,00	35,00	35,00	35,00	35,00	35,00	35,00
hsa-miR-129-1-3p	YP00206074	35,00	35,00	35,00	35,00	35,00	35,00	35,00	35,00
hsa-miR-192-3p	YP00204272	35,00	35,00	35,00	35,00	35,00	35,00	35,00	35,00
hsa-miR-632	YP00205600	35,00	35,00	35,00	35,00	35,00	35,00	35,00	34,88
hsa-miR-181a-2-3p	YP00204142	35,00	34,34	35,00	35,00	35,00	34,01	35,00	35,00
hsa-miR-1909-3p	YP00205697	35,00	35,00	35,00	35,00	35,00	35,00	35,00	35,00
hsa-miR-573	YP00204216	35,00	35,00	35,00	35,00	35,00	35,00	35,00	35,00
hsa-miR-302d-5p	YP00204033	35,00	35,00	35,00	35,00	35,00	35,00	35,00	35,00
hsa-miR-194-3p	YP00204204	35,00	35,00	34,85	35,00	34,25	33,71	34,72	33,07
hsa-miR-302b-5p	YP00205676	35,00	35,00	35,00	35,00	35,00	35,00	35,00	35,00
hsa-miR-551b-5p	YP00204630	35,00	35,00	35,00	35,00	35,00	35,00	35,00	35,00
hsa-miR-635	YP00205936	35,00	35,00	35,00	35,00	35,00	35,00	35,00	35,00
hsa-miR-518d-3p	YP00204628	35,00	35,00	35,00	35,00	35,00	35,00	35,00	35,00
hsa-miR-569	YP00205666	35,00	35,00	35,00	35,00	35,00	35,00	35,00	35,00
hsa-miR-125b-1-3p	YP00204400	30,93	33,36	31,69	33,96	31,00	30,72	32,33	31,07
hsa-miR-218-2-3p	YP00205639	35,00	35,00	35,00	35,00	35,00	35,00	35,00	35,00
hsa-miR-519c-3p	YP00204016	35,00	35,00	35,00	35,00	35,00	35,00	35,00	35,00
hsa-miR-554	YP00204556	35,00	35,00	35,00	35,00	35,00	35,00	35,00	35,00
hsa-miR-938	YP00205912	34,85	35,00	34,90	35,00	35,00	35,00	35,00	35,00
hsa-miR-1243	YP00204211	35,00	35,00	35,00	35,00	35,00	35,00	35,00	35,00
hsa-miR-708-3p	YP00204681	33,39	34,78	35,00	35,00	35,00	33,77	34,89	35,00
hsa-miR-1185-5p	YP00205926	32,07	35,00	32,92	34,29	35,00	33,97	35,00	34,63
hsa-miR-512-3p	YP00204382	35,00	35,00	35,00	35,00	35,00	35,00	35,00	35,00
hsa-miR-587	YP00205876	35,00	35,00	35,00	35,00	35,00	35,00	35,00	35,00
hsa-miR-603	YP00204112	35,00	35,00	35,00	35,00	35,00	35,00	35,00	35,00
hsa-miR-1184	YP00204330	35,00	35,00	35,00	35,00	35,00	35,00	35,00	35,00
hsa-miR-20a-3p	YP00204052	35,00	35,00	35,00	35,00	35,00	35,00	35,00	35,00
hsa-miR-588	YP00205885	35,00	35,00	35,00	35,00	35,00	35,00	35,00	35,00
hsa-miR-455-3p	YP00204035	30,56	31,35	30,62	30,41	32,01	29,45	30,96	30,83
hsa-miR-582-3p	YP00204072	35,00	35,00	35,00	35,00	35,00	35,00	35,00	35,00
hsa-miR-409-5p	YP00204014	31,37	34,19	32,89	35,00	33,79	32,80	35,00	35,00
hsa-miR-452-3p	YP00204201	35,00	35,00	35,00	35,00	35,00	35,00	35,00	35,00
hsa-miR-19b-1-5p	YP00204310	34,74	35,00	34,33	35,00	35,00	34,23	35,00	35,00
hsa-miR-610	YP00204492	35,00	35,00	35,00	35,00	35,00	35,00	35,00	35,00
hsa-miR-511-5p	YP00204046	35,00	35,00	35,00	35,00	35,00	35,00	35,00	35,00
hsa-miR-200c-5p	YP02119294	35,00	35,00	35,00	35,00	35,00	35,00	35,00	35,00
hsa-let-7a-3p	YP00206084	35,00	35,00	35,00	35,00	35,00	35,00	35,00	35,00
hsa-miR-135a-3p	YP00204022	35,00	35,00	35,00	35,00	35,00	35,00	35,00	35,00
hsa-miR-520a-5p	YP00204340	35,00	35,00	35,00	35,00	35,00	35,00	35,00	35,00
hsa-miR-1468-5p	YP00204577	35,00	35,00	35,00	35,00	35,00	35,00	35,00	35,00
hsa-miR-628-5p	YP00205893	35,00	35,00	35,00	35,00	35,00	35,00	35,00	35,00
hsa-miR-552-3p	YP00206032	35,00	35,00	35,00	35,00	35,00	35,00	35,00	35,00
hsa-miR-145-3p	YP00204192	31,02	33,50	32,36	35,00	34,76	32,40	35,00	32,98

hsa-miR-378a-5p	YP00204347	35,00	35,00	35,00	35,00	35,00	35,00	34,60	35,00
hsa-miR-7-1-3p	YP00205888	32,94	35,00	33,31	35,00	35,00	33,84	35,00	35,00
hsa-miR-181c-3p	YP00204213	33,97	35,00	34,71	35,00	35,00	35,00	35,00	35,00
hsa-miR-195-3p	YP00204128	35,00	35,00	35,00	35,00	35,00	34,82	35,00	35,00
hsa-miR-578	YP00204269	35,00	35,00	35,00	35,00	35,00	35,00	35,00	35,00
hsa-miR-505-5p	YP00205657	35,00	35,00	35,00	35,00	35,00	35,00	35,00	35,00
hsa-miR-875-3p	YP00204231	35,00	35,00	35,00	35,00	35,00	35,00	35,00	35,00
hsa-miR-450b-5p	YP00205607	34,82	35,00	34,65	35,00	35,00	34,78	35,00	35,00
hsa-miR-876-5p	YP00204527	35,00	35,00	35,00	35,00	35,00	35,00	35,00	35,00
hsa-miR-362-3p	YP00205612	34,02	35,00	35,00	34,62	34,64	33,20	34,26	33,11
hsa-miR-624-5p	YP00204625	35,00	35,00	35,00	35,00	35,00	33,26	35,00	35,00
hsa-miR-27a-5p	YP00206021	34,42	31,44	33,17	35,00	33,21	32,18	33,41	28,16
hsa-miR-744-3p	YP00206082	34,43	35,00	35,00	35,00	35,00	35,00	35,00	35,00
hsa-miR-139-3p	YP00205661	32,05	33,33	32,28	34,43	31,87	31,29	34,12	32,11
hsa-miR-138-2-3p	YP00204477	35,00	35,00	35,00	35,00	35,00	35,00	35,00	35,00
hsa-miR-655-3p	YP00205645	34,56	35,00	31,75	35,00	35,00	33,84	35,00	32,81
hsa-miR-99b-3p	YP00204064	33,03	34,06	33,67	34,64	33,98	33,87	34,09	35,00
hsa-miR-581	YP00205696	35,00	35,00	35,00	35,00	35,00	35,00	35,00	35,00
hsa-miR-191-3p	YP00204196	35,00	35,00	35,00	35,00	35,00	35,00	35,00	35,00
hsa-miR-32-3p	YP00205648	35,00	35,00	35,00	35,00	35,00	35,00	35,00	35,00
hsa-miR-1204	YP00204123	35,00	35,00	35,00	35,00	35,00	35,00	35,00	35,00
hsa-miR-548j-5p	YP00205861	35,00	35,00	35,00	35,00	35,00	35,00	35,00	35,00
hsa-miR-555	YP00204377	35,00	35,00	35,00	35,00	35,00	35,00	35,00	35,00
hsa-miR-1224-3p	YP00204045	35,00	35,00	35,00	35,00	35,00	35,00	35,00	35,00
hsa-miR-1539	YP00204682	35,00	35,00	35,00	35,00	35,00	35,00	35,00	35,00
hsa-miR-663b	YP00205985	28,54	31,37	27,69	33,66	28,90	28,29	29,14	30,38
hsa-miR-1248	YP00204253	35,00	35,00	35,00	35,00	35,00	35,00	35,00	35,00
hsa-miR-889-3p	YP00204113	32,02	35,00	34,64	33,42	34,80	35,00	35,00	35,00
hsa-miR-1227-3p	YP00204200	35,00	35,00	34,83	35,00	33,55	33,15	35,00	33,31
hsa-miR-548h-5p	YP00204777	35,00	35,00	35,00	35,00	35,00	35,00	35,00	35,00
hsa-miR-1255b-5p	YP00205878	35,00	35,00	35,00	35,00	35,00	35,00	35,00	35,00
hsa-miR-330-5p	YP00204372	35,00	35,00	35,00	35,00	35,00	35,00	35,00	35,00
hsa-miR-1238-3p	YP00204004	35,00	35,00	35,00	35,00	35,00	35,00	35,00	35,00
hsa-miR-188-3p	YP02119296	35,00	35,00	35,00	35,00	35,00	35,00	35,00	35,00
hsa-miR-589-3p	YP00206048	35,00	35,00	35,00	35,00	35,00	35,00	35,00	35,00
hsa-miR-125b-2-3p	YP00204295	33,16	35,00	33,28	35,00	34,95	33,42	35,00	34,09
hsa-miR-16-2-3p	YP00204309	35,00	35,00	33,26	35,00	35,00	34,68	34,81	33,86
hsa-miR-515-5p	YP00204431	35,00	35,00	35,00	35,00	35,00	35,00	35,00	35,00
hsa-miR-340-3p	YP00204250	31,06	31,04	34,11	35,00	35,00	35,00	28,48	31,40
hsa-miR-513a-3p	YP00204597	35,00	35,00	35,00	35,00	35,00	35,00	35,00	35,00
hsa-miR-34a-3p	YP00206061	31,49	33,94	31,16	32,18	32,06	30,37	32,10	30,97
hsa-miR-342-5p	YP00204516	35,00	35,00	35,00	35,00	35,00	35,00	35,00	35,00
hsa-miR-639	YP02119298	35,00	35,00	35,00	35,00	35,00	35,00	35,00	35,00
hsa-let-7i-3p	YP00204247	33,01	35,00	31,21	35,00	35,00	32,37	35,00	33,63
hsa-miR-543	YP00204447	30,79	32,41	32,33	33,96	32,32	31,32	32,97	32,91
hsa-miR-645	YP00206076	35,00	35,00	35,00	35,00	34,87	34,34	35,00	35,00

hsa-miR-548d-5p	YP00205907	35,00	35,00	35,00	35,00	35,00	35,00	35,00	35,00
hsa-miR-33a-3p	YP00204086	35,00	35,00	35,00	35,00	35,00	35,00	35,00	35,00
hsa-miR-664a-3p	YP00204725	29,99	29,46	27,83	29,26	29,89	28,20	29,67	29,12
hsa-miR-379-3p	YP00204345	33,39	35,00	33,71	35,00	35,00	33,76	35,00	35,00
hsa-miR-556-3p	YP00205717	35,00	35,00	35,00	35,00	35,00	35,00	35,00	35,00
hsa-miR-614	YP00204273	35,00	35,00	35,00	35,00	35,00	35,00	35,00	35,00
hsa-miR-616-5p	YP00205611	35,00	35,00	35,00	35,00	35,00	35,00	35,00	35,00
hsa-miR-93-3p	YP00204470	33,09	35,00	34,02	35,00	35,00	33,55	35,00	34,17
hsa-miR-1972	YP00205605	33,56	34,00	33,47	34,50	33,10	32,48	33,62	30,95
hsa-miR-616-3p	YP00204116	35,00	35,00	35,00	35,00	35,00	35,00	35,00	35,00
hsa-miR-369-3p	YP00206028	34,14	35,00	32,84	32,49	35,00	33,99	35,00	33,70
hsa-miR-2110	YP00204328	33,32	33,83	31,59	33,42	32,24	31,85	33,47	32,32
hsa-miR-548a-3p	YP00205650	35,00	35,00	35,00	35,00	35,00	35,00	35,00	34,93
hsa-miR-634	YP00204228	35,00	35,00	35,00	35,00	35,00	35,00	35,00	35,00
hsa-miR-320c	YP00205706	28,77	30,50	28,28	30,66	30,02	29,59	30,70	30,07
hsa-miR-636	YP00204298	35,00	35,00	35,00	35,00	35,00	35,00	35,00	35,00
hsa-miR-606	YP00205629	35,00	35,00	35,00	35,00	35,00	35,00	35,00	35,00
hsa-miR-208b-3p	YP00204636	35,00	35,00	35,00	35,00	35,00	35,00	35,00	35,00
hsa-miR-367-5p	YP00205652	35,00	35,00	35,00	35,00	35,00	35,00	35,00	35,00
hsa-miR-520d-3p	YP00204040	35,00	35,00	35,00	35,00	35,00	35,00	35,00	35,00
hsa-miR-1265	YP00204082	35,00	35,00	35,00	35,00	35,00	35,00	35,00	35,00
hsa-miR-1203	YP00204414	35,00	35,00	35,00	35,00	35,00	35,00	35,00	35,00
hsa-miR-548k	YP00205906	35,00	35,00	35,00	35,00	35,00	35,00	35,00	35,00
hsa-miR-548a-5p	YP00204416	35,00	35,00	35,00	35,00	35,00	35,00	35,00	35,00
hsa-miR-1253	YP00204525	35,00	35,00	35,00	35,00	35,00	35,00	35,00	35,00
hsa-miR-615-5p	YP00204445	35,00	35,00	35,00	35,00	35,00	35,00	35,00	35,00
hsa-miR-607	YP00204649	35,00	35,00	35,00	35,00	35,00	35,00	35,00	35,00
hsa-miR-1208	YP00205937	35,00	35,00	35,00	35,00	35,00	35,00	35,00	35,00
hsa-miR-302e	YP00205858	35,00	35,00	35,00	35,00	35,00	35,00	35,00	35,00
hsa-miR-1206	YP00204147	35,00	35,00	35,00	35,00	35,00	35,00	35,00	35,00
hsa-miR-1270	YP00204025	35,00	35,00	35,00	35,00	35,00	35,00	35,00	35,00
hsa-miR-525-3p	YP00204111	35,00	35,00	35,00	35,00	35,00	35,00	35,00	34,91
hsa-miR-1200	YP00204044	35,00	35,00	35,00	35,00	35,00	35,00	35,00	35,00
hsa-miR-1911-5p	YP00204595	35,00	35,00	35,00	35,00	35,00	35,00	35,00	35,00
hsa-miR-33b-3p	YP00204462	35,00	35,00	35,00	35,00	35,00	35,00	35,00	35,00
hsa-miR-223-5p	YP00204529	35,00	35,00	35,00	35,00	35,00	35,00	35,00	35,00
hsa-miR-34b-5p	YP00204424	31,48	33,62	34,44	35,00	34,44	34,55	34,28	33,70
hsa-miR-888-3p	YP00205934	35,00	35,00	35,00	35,00	35,00	35,00	35,00	35,00
hsa-miR-424-3p	YP00205918	33,62	35,00	33,32	35,00	31,16	28,44	29,54	29,39
hsa-miR-339-3p	YP00204160	33,31	35,00	33,85	35,00	34,11	34,17	35,00	35,00
hsa-miR-380-5p	YP00205903	35,00	35,00	35,00	35,00	34,70	35,00	35,00	33,08
hsa-miR-647	YP00204027	35,00	35,00	35,00	35,00	35,00	35,00	35,00	35,00
hsa-miR-518f-5p	YP00204671	35,00	35,00	26,60	35,00	35,00	35,00	35,00	31,32
hsa-miR-92b-5p	YP00204415	35,00	35,00	35,00	35,00	35,00	34,98	35,00	35,00
hsa-miR-551a	YP00204331	35,00	35,00	35,00	35,00	35,00	35,00	35,00	35,00
hsa-miR-146a-3p	YP00204401	35,00	35,00	35,00	35,00	35,00	35,00	35,00	35,00

hsa-miR-218-1-3p	YP00204691	35,00	35,00	34,18	35,00	35,00	35,00	35,00	35,00
hsa-miR-593-5p	YP00204550	35,00	35,00	35,00	35,00	35,00	35,00	35,00	35,00
hsa-miR-561-3p	YP00205895	35,00	35,00	35,00	35,00	35,00	35,00	35,00	35,00
hsa-miR-767-3p	YP02119295	31,73	35,00	35,00	35,00	35,00	35,00	31,23	35,00
hsa-miR-526b-3p	YP00205984	35,00	35,00	35,00	35,00	35,00	35,00	35,00	35,00
hsa-miR-24-1-5p	YP00204357	34,32	35,00	34,80	35,00	35,00	35,00	35,00	35,00
hsa-let-7b-3p	YP00205653	30,99	33,17	30,89	33,41	31,46	30,60	32,25	31,65
hsa-miR-193b-5p	YP00204065	33,99	35,00	35,00	35,00	34,62	33,48	34,23	34,46
hsa-miR-335-3p	YP00205613	35,00	31,01	32,63	32,12	33,95	33,37	35,00	35,00
hsa-miR-541-5p	YP00204118	35,00	35,00	35,00	35,00	35,00	35,00	35,00	35,00
hsa-miR-30c-1-3p	YP00205707	35,00	35,00	35,00	35,00	35,00	35,00	35,00	35,00
hsa-miR-629-3p	YP00204210	35,00	35,00	34,68	35,00	35,00	35,00	35,00	35,00
hsa-miR-377-5p	YP00205428	34,15	34,49	35,00	35,00	35,00	35,00	35,00	35,00
hsa-miR-630	YP00204392	35,00	35,00	35,00	35,00	35,00	35,00	35,00	35,00
hsa-miR-548d-3p	YP00205608	34,92	35,00	35,00	35,00	35,00	34,91	35,00	35,00
hsa-miR-885-3p	YP00204136	30,29	33,75	35,00	31,41	34,78	32,74	32,03	30,59
hsa-miR-320d	YP00205667	31,45	32,31	29,38	32,60	31,98	31,57	32,12	32,67
hsa-miR-2053	YP00204605	35,00	35,00	35,00	35,00	35,00	35,00	35,00	35,00
hsa-miR-675-5p	YP00205687	35,00	35,00	35,00	35,00	32,96	35,00	35,00	35,00
hsa-miR-1252-5p	YP00204701	35,00	35,00	35,00	35,00	35,00	35,00	35,00	35,00
hsa-miR-548e-3p	YP00206005	35,00	35,00	35,00	35,00	35,00	35,00	35,00	35,00
hsa-miR-1914-5p	YP00204672	34,86	35,00	35,00	35,00	35,00	34,40	35,00	33,71
hsa-miR-513c-5p	YP00205928	34,53	35,00	26,23	31,08	35,00	29,85	33,18	30,51
hsa-miR-331-5p	YP00204423	35,00	35,00	34,72	35,00	35,00	35,00	35,00	35,00
hsa-miR-1182	YP00204466	35,00	35,00	35,00	35,00	35,00	35,00	35,00	35,00
hsa-miR-611	YP00204041	35,00	35,00	35,00	35,00	35,00	35,00	35,00	35,00
hsa-miR-1181	YP00204050	35,00	35,00	35,00	35,00	34,99	33,68	35,00	34,05
hsa-miR-638	YP00204234	32,35	33,14	30,74	32,04	29,81	29,88	30,32	30,86
hsa-miR-515-3p	YP00204651	35,00	35,00	35,00	35,00	35,00	35,00	35,00	35,00
hsa-miR-650	YP00204233	35,00	35,00	35,00	35,00	35,00	35,00	35,00	33,91
hsa-miR-1178-3p	YP00205870	35,00	35,00	35,00	35,00	35,00	35,00	35,00	35,00
hsa-miR-600	YP00204076	35,00	35,00	35,00	35,00	35,00	35,00	35,00	35,00
hsa-miR-599	YP00205908	35,00	35,00	35,00	35,00	35,00	35,00	35,00	35,00
hsa-miR-520g-3p	YP00204055	35,00	35,00	35,00	35,00	35,00	35,00	35,00	35,00
hsa-miR-564	YP00205925	35,00	35,00	35,00	35,00	35,00	35,00	35,00	35,00
hsa-miR-132-5p	YP00204552	32,56	35,00	34,03	33,45	35,00	35,00	35,00	35,00
hsa-miR-577	YP00205998	35,00	35,00	35,00	35,00	35,00	35,00	35,00	35,00
hsa-miR-1911-3p	YP00204599	35,00	35,00	31,70	35,00	33,03	32,37	32,59	33,25
hsa-let-7f-2-3p	YP00204095	31,76	34,41	32,41	35,00	35,00	32,49	35,00	32,40
hsa-miR-155-3p	YP00204000	34,80	35,00	35,00	35,00	35,00	35,00	35,00	35,00
hsa-miR-105-3p	YP00204193	35,00	35,00	35,00	35,00	35,00	35,00	35,00	35,00
hsa-miR-486-3p	YP00204107	35,00	35,00	35,00	35,00	35,00	35,00	35,00	35,00
hsa-miR-320b	YP02119299	28,69	30,94	29,11	30,02	30,24	29,66	31,22	31,14
hsa-miR-296-3p	YP00204393	35,00	35,00	35,00	35,00	35,00	35,00	35,00	35,00
hsa-miR-7-2-3p	YP00204581	35,00	35,00	35,00	35,00	35,00	35,00	35,00	35,00
hsa-miR-550a*	YP00204795	34,08	34,64	35,00	35,00	35,00	35,00	35,00	35,00

hsa-miR-380-3p	YP00204586	35,00	35,00	34,76	35,00	35,00	35,00	35,00	35,00
hsa-miR-593-3p	YP00204083	35,00	35,00	35,00	35,00	35,00	35,00	35,00	35,00
hsa-miR-1912	YP00205866	35,00	35,00	35,00	35,00	35,00	35,00	35,00	35,00
hsa-miR-493-5p	YP00204166	31,95	33,41	31,45	35,00	34,18	32,74	35,00	34,19
hsa-miR-432-3p	YP00205894	34,39	35,00	35,00	35,00	35,00	35,00	35,00	35,00
hsa-miR-454-5p	YP00204279	35,00	35,00	35,00	35,00	35,00	35,00	35,00	35,00
hsa-miR-936	YP00204185	34,94	35,00	35,00	35,00	33,75	35,00	35,00	35,00
hsa-miR-30a-3p	YP00204457	31,10	32,28	32,75	35,00	34,05	32,39	34,68	34,53
hsa-let-7g-3p	YP00205865	33,01	35,00	34,72	35,00	35,00	32,71	34,80	32,82
hsa-miR-214-5p	YP00204575	28,90	33,34	30,40	33,26	32,07	30,35	32,60	30,47
hsa-miR-183-3p	YP00205673	35,00	35,00	35,00	35,00	35,00	35,00	35,00	35,00
hsa-miR-1179	YP00204624	34,69	34,63	35,00	35,00	35,00	35,00	35,00	35,00
hsa-miR-562	YP00205941	35,00	35,00	35,00	35,00	35,00	35,00	35,00	35,00
hsa-miR-579-3p	YP00204627	35,00	35,00	35,00	35,00	35,00	35,00	35,00	35,00
hsa-miR-590-3p	YP00205448	34,01	35,00	34,03	35,00	35,00	35,00	35,00	35,00
hsa-miR-130a-5p	YP00204329	35,00	35,00	35,00	35,00	35,00	35,00	35,00	35,00
hsa-miR-563	YP00205618	35,00	35,00	35,00	35,00	35,00	35,00	35,00	35,00
hsa-miR-200a-5p	YP00206063	35,00	35,00	35,00	35,00	35,00	35,00	35,00	35,00
hsa-miR-483-5p	YP00205693	35,00	35,00	35,00	35,00	35,00	35,00	35,00	35,00
hsa-miR-15a-3p	YP00204435	34,43	35,00	34,28	35,00	35,00	35,00	35,00	35,00
hsa-miR-944	YP00205700	35,00	35,00	35,00	35,00	35,00	35,00	35,00	35,00
hsa-miR-92a-2-5p	YP00204277	35,00	35,00	35,00	35,00	35,00	35,00	35,00	35,00
hsa-miR-548n	YP00205705	35,00	35,00	35,00	35,00	35,00	35,00	35,00	35,00
